# RecF protein targeting to post-replication (daughter strand) gaps II: RecF interaction with replisomes

**DOI:** 10.1093/nar/gkad310

**Published:** 2023-05-01

**Authors:** Camille Henry, Gurleen Kaur, Megan E Cherry, Sarah S Henrikus, Nina J Bonde, Nischal Sharma, Hope A Beyer, Elizabeth A Wood, Sindhu Chitteni-Pattu, Antoine M van Oijen, Andrew Robinson, Michael M Cox

**Affiliations:** Department of Biochemistry, University of Wisconsin-Madison, Madison, WI53706-1544, USA; Molecular Horizons Institute and School of Chemistry and Molecular Bioscience, University of Wollongong, Wollongong, Australia; Illawarra Health and Medical Research Institute, Wollongong, Australia; Molecular Horizons Institute and School of Chemistry and Molecular Bioscience, University of Wollongong, Wollongong, Australia; Illawarra Health and Medical Research Institute, Wollongong, Australia; Molecular Horizons Institute and School of Chemistry and Molecular Bioscience, University of Wollongong, Wollongong, Australia; Illawarra Health and Medical Research Institute, Wollongong, Australia; Department of Biochemistry, University of Wisconsin-Madison, Madison, WI53706-1544, USA; Molecular Horizons Institute and School of Chemistry and Molecular Bioscience, University of Wollongong, Wollongong, Australia; Illawarra Health and Medical Research Institute, Wollongong, Australia; Department of Biochemistry, University of Wisconsin-Madison, Madison, WI53706-1544, USA; Department of Biochemistry, University of Wisconsin-Madison, Madison, WI53706-1544, USA; Department of Biochemistry, University of Wisconsin-Madison, Madison, WI53706-1544, USA; Molecular Horizons Institute and School of Chemistry and Molecular Bioscience, University of Wollongong, Wollongong, Australia; Illawarra Health and Medical Research Institute, Wollongong, Australia; Molecular Horizons Institute and School of Chemistry and Molecular Bioscience, University of Wollongong, Wollongong, Australia; Illawarra Health and Medical Research Institute, Wollongong, Australia; Department of Biochemistry, University of Wisconsin-Madison, Madison, WI53706-1544, USA

## Abstract

The bacterial RecF, RecO, and RecR proteins are an epistasis group involved in loading RecA protein into post-replication gaps. However, the targeting mechanism that brings these proteins to appropriate gaps is unclear. Here, we propose that targeting may involve a direct interaction between RecF and DnaN. *In vivo*, RecF is commonly found at the replication fork. Over-expression of RecF, but not RecO or a RecF ATPase mutant, is extremely toxic to cells. We provide evidence that the molecular basis of the toxicity lies in replisome destabilization. RecF over-expression leads to loss of genomic replisomes, increased recombination associated with post-replication gaps, increased plasmid loss, and SOS induction. Using three different methods, we document direct interactions of RecF with the DnaN β-clamp and DnaG primase that may underlie the replisome effects. In a single-molecule rolling-circle replication system *in vitro*, physiological levels of RecF protein trigger post-replication gap formation. We suggest that the RecF interactions, particularly with DnaN, reflect a functional link between post-replication gap creation and gap processing by RecA. RecF’s varied interactions may begin to explain how the RecFOR system is targeted to rare lesion-containing post-replication gaps, avoiding the potentially deleterious RecA loading onto thousands of other gaps created during replication.

## Graphical Abstract

**Graphical Abstract:**
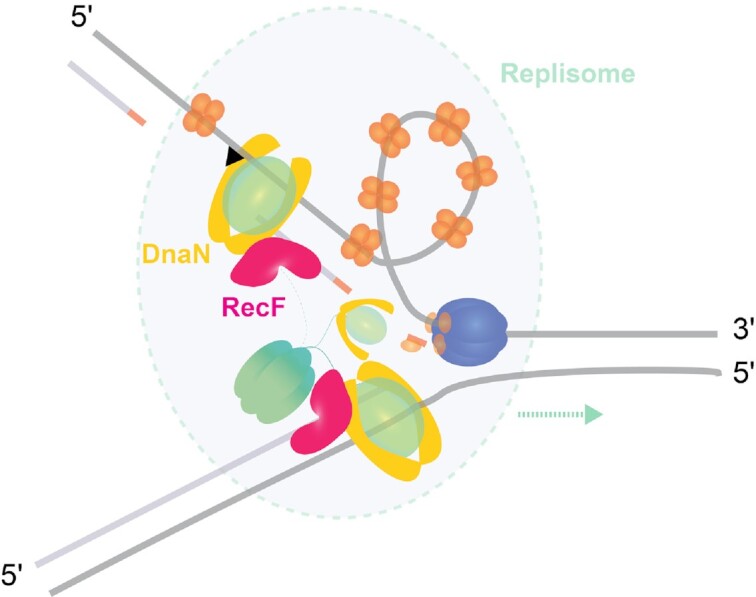
RecF interacts with DnaN.

## INTRODUCTION

The accurate replication of genetic information is an essential process allowing cell proliferation and genome stability. The replisome is a multienzyme complex formed by more than ten components. In bacteria, DNA replication starts from the origin, proceeds bidirectionally and ends at the terminus site (1,2). During replication, cells experience endogenous or exogenous stresses causing DNA damage. Encounters of the replication machinery with unrepaired DNA damage can lead to replication stalling or collapse (3,4). In some cases, encounters with template lesions do not halt replisome progress. Instead, the replisome can bypass the lesion and be reprimed downstream, leading to the formation of a lesion-containing post-replication gap (5–7). Single-strand DNA post-replication gaps are formed frequently, perhaps several times per replication cycle under normal growth conditions (8,9). Despite decades of work, the formation and repair of post-replication gaps remains one of the least understood processes in DNA metabolism. Once formed, the gap will usually contain a lesion and accurate repair requires an undamaged complementary strand. Post replication gaps can be filled by three different mechanisms: (i) homologous recombination (10–12), (ii) RecA-independent template switching (13–16) and (iii) translesion DNA synthesis (11,17,18). RecA-dependent homologous recombination predominates.

To repair a lesion-containing post-replication gap, the RecA protein must find that gap and distinguish it from other gaps that occur normally during replication and do not require repair. Research over the last 5 decades has implicated the RecF, RecO and RecR proteins in two relevant functions: (a) targeting repair specifically to lesion-containing post-replication gaps and (b) loading RecA onto single-stranded DNA (ssDNA) within those gaps through the displacement of the single-stranded DNA binding protein SSB (19–22). The grouping of *recF*, *recO* and *recR* genes into an epistasis group has been substantiated by a range of genetic observations (23–32). However, a stable complex containing all three proteins has not been observed. For this reason, the term RecFOR will be used here only when referring to the system or pathway. The loading function is embedded in the RecO and RecR proteins, which form a complex that is necessary and sufficient for loading RecA protein onto SSB-coated ssDNA *in vitro* and *in vivo* (20,21,33–35). RecO but not RecF binds directly to the C-terminus of SSB and RecOR loads RecA protein at more or less random sites within a ssDNA gap (10,33). The targeting function we wish to explore in the current study appears to be centered on RecF.

RecF is a member of the ATP-binding cassette proteins (ABC) and harbors the Walker A, Walker B and the Signature domains characteristic to this superfamily (36,37). Structurally, RecF is similar to structural maintenance of chromosome proteins (SMC) and notably to the head domain of Rad50, a eukaryotic ABC protein involved in double strand break repair (36,37). RecF forms a dimer in which two ATP molecules are located at the interface created between the Walker A and signature domains of two opposite monomers. *In vitro*, RecF protein binds single strand (ss) (38) and double strand (ds) DNA. The binding to DNA is highly ATP dependent (37,39–41). RecF exhibits a weak DNA-dependent ATPase activity governing its dissociation from the dsDNA (42). Therefore, RecF binding to dsDNA can be enhanced if ATP hydrolysis is blocked, either by using the non-hydrolyzable ATP analogue ATPγS or a RecF mutant protein lacking ATPase function such as RecF_K36R_ (42).

RecF also makes a complex with RecR (41,43–45). Both RecO and RecF compete for RecR binding (45). A more stable RecF-dsDNA complex is formed when the RecF dimer is stabilized through interaction with RecR protein to form the RecFR complex, although in that case an increase in ATPase activity is also observed revealing faster recycling (40,41,43). RecF or RecFR will also bind to ssDNA (37,38,40,41,43).

The most prominent hypothesis proposed to date for RecF targeting to post-replication gaps envisions specific binding of RecF and/or RecFR to the ends of gaps (12,20,46–49). This hypothesis has become unworkable for at least two reasons. First, strong binding by RecF to gap ends would presumably be problematic, as there would be no way to distinguish the occasional lesion-containing post-replication gaps where RecF activity is needed and the much more plentiful gaps generated by lagging strand DNA synthesis, along with much less common gaps produced by mismatch repair and other processes. Unneeded and potentially deleterious DNA joint molecules linking the sister chromosomes could be generated behind the replication fork. Second and importantly, RecF exhibits no strong binding preference for a DNA end or a ds/ss junction at the end of a gap (40,42,50), a status established systematically by the accompanying manuscript (40).

The mechanism by which the RecFOR system is productively targeted to lesion-containing post-replication gaps is thus unclear. If RecF is involved in targeting but does not bind specifically to gap ends, the targeting function must be found in other RecF interactions. An interaction with one or more proteins found at or near post-replication gaps becomes an important possibility. The most obvious interaction candidates are SSB and the replisome. RecF does not interact with SSB (10, 21, 33, this work).

In addition, the promiscuous RecA loading function of RecOR seen *in vitro* must somehow be constrained in the cell so that RecA filaments are not loaded into gaps that do not require repair. Constraining RecOR-mediated loading of RecA protein means blocking RecO interactions with RecR, SSB, or both. RecF can enhance the RecOR-mediated loading of RecA onto SSB-coated ssDNA (46,47,50), but to date this has only been observed under two conditions *in vitro* where the interaction of RecO protein with ssDNA-bound SSB is blocked. As described further in the Discussion, the details of experiments that detected a RecF stimulation of RecOR function might reflect a RecR handoff between RecF and RecO.

Recent studies have begun to phenotypically distinguish the RecF and RecO proteins that might reflect the RecF (targeting) and RecO (RecA loading) division of labor within the RecFOR system. Resistance to particular DNA damaging agents is more dependent on RecF than RecO and vice versa (51–53). After DNA damage or replication fork stress, only RecO and RecR are essential for RecA foci formation in *Bacillus subtilis* (51). Similarly, after DNA damage, RecO and RecR, but not RecF, are required for nucleoid compaction observed in *E. coli* (53). In contrast, of the RecFOR proteins, only the RecF protein is toxic to cells when over-expressed (54–56). This represents a key and dramatic distinction between RecO and RecF. Moreover, RecF’s deleterious effect is suppressed by RecOR co-expression, suggesting a compensatory effect (55).

Another factor distinguishing RecF from RecO is a growing literature, based on both experimentation and speculation, linking RecF protein to a function at the replisome (44,52,57–60). The recent ability to visualize single-molecules in living cells has demonstrated that RecF and RecO do not colocalize and exhibit very different spatiotemporal behavior (52). Whereas RecO is generally found at sites distal to the replisome, RecF often colocalizes with the replication fork (52). RecF is required for rapid resumption of DNA synthesis after cells are UV irradiated and prevents extensive DNA degradation from occurring (58,61,62). These functions require the RecF ATPase (62). Intriguingly, the *recF* gene is located adjacent to *dnaN* in an operon otherwise devoted to replication, an evolutionary relationship that could be accidental but has never been rationalized (63,64).

Could a RecF interaction with the replisome explain the targeting of the RecFOR system to post-replication gaps? Post-replication gaps are created when a replisome encounters a lesion and disengages from the template. With a RecF-replisome interaction, proper placement of RecF for repair purposes could, in principle, be coupled to replisome disengagement to create the gap requiring repair. In this report, we explore the function that the RecF protein might have at the replication fork. We document an interaction between RecF and replisome components (particularly DnaN), investigate how RecF affects replisomes, and provide evidence suggesting that replisome destabilization is at the heart of the toxicity seen when RecF concentration is increased. When combined with studies already published, the observations may help explain how lesion-containing post-replication gaps are distinguished from other gaps, how RecF protein finds its way to the particular gap where it is needed, and how the potentially deleterious RecA-loading function of RecOR might be constrained.

## MATERIALS AND METHODS

### Strains and plasmids

All the strains and plasmids used in this study are listed in the Tables 1 and 2 below. Strain were constructed using λ_RED_ recombination (65) or P1 transduction as indicated. All constructions were confirmed by PCR and sequenced as required.

**Table 1.** Strains used in this study

Strain	Relevant genotype	Parent strain	Source/technique
MG1655	*recF-wt ssb-wt* (wild type - wt)	-	(66)
EAW629	Δ*recF::Kan*	MG1655	(52)
EAW779	*recF-mKate2::Kan*	MG1655	(52)
CJH0015	*recF-mKate2::FRT dnaX-YPet::Kan*	MG1655	(52)
EAW1276	Δ*recF::FRT dnaX-YPet::Kan*	EAW629	This study - P1 JJC5945
EAW1169	*ssb-mTur2::Kan*	MG1655	(67)
CJH0080	Δ*recF::FRT ssb-mTur2::Kan*	EAW629	This study - P1 EAW1169
EAW1130	P_BAD_*-recF::Kan*	MG1655	This study - λ_RED_
EAW1148	P_BAD_*-recF_K36R_::Kan*	MG1655	This study - λ_RED_
EAW114	Δ*recO::Kan*	MG1655	(52)
EAW1190	*recF_K36R_::Kan*	MG1655	This study - λ_RED_
ZJR04	Δ*uup::FRT* Δ*radD::Kan*	MG1655	(8)
EAW1063	Δ*uup::FRT* Δ*radD::FRT* Δ*recF::Kan*	ZJR04	(8)
EAW1064	Δ*uup::FRT* Δ*radD::FRT* Δ*recO::Kan*	ZJR04	(8)
CJH0115	Δ*uup::FRT* Δ*radD::FRT recF_K36R_::Kan*	ZJR04	This study - P1 EAW1190
EAW408	Δ*lacIZYA*	MG1655	(8)
EAW1400	Δ*lacIZYA* P_BAD_*-recF::Kan*	EAW408	This study - P1 EAW1130
EAW1401	Δ*lacIZYA* P_BAD_*-recF_K36R_::Kan*	EAW408	This study - P1 EAW1148
DH5α	cloning strain	-	(68)
CFy7	*Saccharomyces cerevisiae* -Yeast strain		(69)
STL2669	(*ΔrecA-srlR)306::Tn10 TetRxonA2 (exoI-)*		Susan Lovett gift
BL21 λDE3 ΔslyD	*F–ompT hsdSB (rB–, mB–) gal dcm (DE3) ΔslyD*		Cox Lab collection

**Table 2.** Plasmids used in this study

Plasmid name	Description	Source/technique
pBAD	pBAD NcoI/NdeI, Amp (pBAD/mycHisA Invitrogen derivatives)	Cox Lab collection
pRecF	(pEAW1187) pBAD*-recF*, Amp	(52)
pRecF_K36R_	(pEAW1188) pBAD*-recF_K36R_*, Amp	This study
pRecF-mKate2	(pEAW1128) pBAD-*recF-mKate2*, Amp	This study
pRecF_K36R_-mKate2	(pEAW1202) pBAD*-recF_K36R_-mKate2*, Amp	This study
pBR322	Amp Tet	(70)
pSTL78	101 bp with 7.1 kb, Amp Tet	(71)
pSTL74	101 bp with 1.4 kb, Amp Tet	(71)
pMB302	101 bp cruciform from a110 bp palindrome, Amp Tet	(72)
pRC7	pRC7-*lacZYA*, Amp	(73)
pEAW1232	pBR322-*lacIZYA*, Amp-Tet	This study
pPrecN-gfp	(pEAW903) *PrecN-gfp*, Amp	(52)
pGAD-C3	pGAD-C3, LEU2, Amp	(69)
pEAW1241	pGAD-RecF	This study
pEAW1243	pGAD-RecF_K36R_	This study
pEAW1247	pGAD-RecR	This study
pEAW1258	pGAD-RecO	This study
pEAW1157	pGAD-RecA	This study
pGAD-YejH	pGAD-RadD	(74)
pEAW1158	pGAD-RecG	(75)
pSW032	pGAD-DnaC	(76)
pEAW1115	pGAD-DnaE	This study
pEAW1101	pGAD-DnaN	This study
pEAW1249	pGAD-DnaG	This study
pEAW1251	pGAD-TopB	This study
pEAW1100	pGAD-RecQ	This study
pGBD-C3	pGBD-C3, TRP1, AmpR	(69)
pEAW1242	pGBD-RecF	This study
pEAW1244	pGBD-RecF_K36R_	This study
pEAW1248	pGBD-RecR	This study
pEAW1259	pGBD-RecO	This study
pEAW1155	pGBD-RecA	This study
pGBD-YejH	pGBD-RadD	(74)
pEAW1156	pGBD-RecG	(75)
pSW033	pGBD-DnaC	(76)
pEAW1116	pGBD-DnaE	This study
pEAW1104	pGBD-DnaN	This study
pEAW1250	pGBD-DnaG	This study
pEAW1252	pGBD-TopB	This study
pEAW1105	pGBD-RecQ	This study
pBLW20	pET21A-RecFwt (codon optimized), Amp	(43)
pBLW24	pET21A-RecF_K36R_ (codon optimized), Amp	(42)
pEAW1290	pET21A-RecF-mKate2, Amp	This study
pEAW1300	pET28A-His-mKate2, Amp	This study
pJOE-recR	(pCJH0006) pJOE-recR, Rhamnose inducible, Amp	(40)
pT7pol26	pT7pol26, Kan	Cox Lab collection

For the EAW1130 (P_BAD_*-recF*) and EAW1148 (P_BAD_*-recF_K36R_*) *E. coli* K-12 MG1655 derivative strains, the *araBAD* promoter was inserted in the front of the start codon (ATG) of *recF* or *recF_K36R_* to replace the native promoter. Briefly, a sequence containing a transcription terminator at the end *dnaN* followed by the *araBAD* promoter in the front of the *recF* or *recF_K36R_* genes was cloned into a plasmid and then amplified by PCR. For all constructs, PCR fragments were gel purified and integrated onto the chromosome using λ_RED_ recombination (65). For *recF-mKate2* mutants the promoter region of *gyrB* was duplicated to maintain the *gyrB* promoter region. This maintained normal *gyrB* expression (Figure 2B).

### Cloning

The *lacIZ* region of the pRC7 vector was amplified by PCR using the following primers, BsmI/lacIq up2: 5′-CGGATAGAATGCGCAATTCGGGACACCATCGAATGGTGCAAAAC and BsmI/lacZ rev2: 5′-CGGATAGAATGCGTGTTTTTTAAATAGTACATAATGGATTTCCTTA. The PCR product was DpnI digested for 1 h, gel purified, and digested with BsmI in order to be ligated into a pBR322 plasmid linearized by BsmI and dephosphorylated. The ligation product was transformed into DH5α competent cells (*lac^–^*). After one hour of growth, the transformed cells were spread on plates supplemented with ampicillin. The resulting vector is pEAW1232.

The open reading frame (start to stop codons) of the following genes: *recF*, *recF_K36R_*, *recR*, *recO*, *recG*, *dnaC*, *dnaE*, *dnaN*, *dnaG*, *topB* and *recQ* were amplified and subcloned in frame into pGAD-C3 and pGBD-C3 to generate N-terminal fusion with GAL4 either the activation domain (AD) or binding domain (BD). The resulting plasmid were attributed a pEAW number (see plasmid list) and identified as pGAD- protein of interest or pGBD- protein of interest.

The RecF-mKate2 was subcloned from pEAW1128 into pET21A vector by double digestion EcoRI and NdeI. Finally, the plasmid encoding His-mKate2 was generated as followed. The pEAW1290 (recF-mKate2 in pET21A), was digested with BglII and EcoRI and the small DNA band containing the linker-mKate2 region, was ligated to pET28A cut with BamHI and EcoRI. The resulting plasmid was directly sequenced to confirm it was linker-mKate in pET28A.

### Media and culture condition

Chemicals and media were purchased from Fisher, Sigma, Biolabs, AlphaAesar or Teknova. Cells were grown in rich media Luria Bertani (LB), or in EZ supplemented with 0.2% glycerol. l-Arabinose (Ara) was purchased from Fisher (Acros Organics), 20% stock was made by resuspending the Ara in ultrapure water and filter sterilization. When required, antibiotics were added at the following concentrations: ampicillin 100 μg/ml, kanamycin 40 μg/ml and tetracycline 15 μg/ml.

### Over-expression assay

The RecF_wt_ and its ATPase dead variant RecF_K36R_ unlabelled or labelled overexpression was carried out in different conditions to study new aspects of RecF function. For protein over-expression from plasmids, cell cultures were inoculated with 1:100 ratio of an overnight (ON) culture grown in the same condition, i.e. in LB (or EZ glycerol) supplemented with ampicillin. Cells were grown at 37°C to mid-log phase OD_600_: 0.2–0.4, then 0.2% of Ara was added to induce over-expression. For the microscopy experiments, all experiments were carried out in EZ 0.2% glycerol. When the chromosomal constructions were used for over-expression, culture were inoculated with a 1:1000 ratio from ON culture, then cells were grown for 16 h at 37°C with various concentrations of Ara (0 to 10%). In order to use high concentrations of Ara, a 2x LB was prepared and mixed with the adequate volume of 20% Ara and completed with ultrapure sterilized water to reach the final volume.

Survival was assayed after Ara addition at the indicated time by spotting assay. Briefly, cells were serial diluted in phosphate buffered saline (PBS 1x, pH 7.4) by a factors of ten and 10 μl of the indicated dilutions were spotted on LB plates (supplemented with ampicillin for cells carrying pBAD or pBR322 derivative vectors). Plates were incubated overnight at 37°C, then images were taken using a LAS (GE Healthcare) or iBright (ThermoFisher) imagers.

### Protein detection by coomassie staining or immunoblot

The production level in total cell extracts of a variety of proteins of interest from this study was determined by Coomassie stained SDS PAGE gel and/or immunoblot. Cells were harvested at the indicated time. Cell pellets were directly resuspended in adequate volume of cracking buffer (CB) composed of 10% glycerol, 125 mM TrisCl pH 6.8, 2% SDS, 5% 2-betamercaptoethanol and 0.5 mg/ml Bromophenol blue. The volume of CB added was adjusted to the OD_600_ (the volume use was calculated to be equivalent to 100 μl of CB for a pellet of 1 ml of cells at OD_600_:1). Whole cell extract samples were boiled at 95°C 10 min and loaded on 12% SDS PAGE. After electrophoresis, gels were either directly stained with Coomassie blue, or submitted to liquid electrophoresis to transfer proteins to a nitrocellulose membrane for immunoblot. Membranes were saturated 30 min at room temperature in 5% milk, 1× PBS supplemented by Tween 0.05% volume (PBS-T). A fresh milk solution was used to incubate membranes with the first antibody diluted at 1:3000 (Rabbit anti-RFP from Invitrogen), 1:5000 (Rabbit anti-RecF, Cox Lab), 1:200 (Rabbit anti GyrB from Creative Diagnostic or Rabbit anti-SSB from the Keck Lab). Incubation with first antibody was carried out for 1 h 30 min at room temperature or alternatively overnight at 4°C. Membranes were washed in PBS-T 4 times for 3 min, then incubated 1h in PBS-T with the second anti-body diluted 1:5000 (Goat anti Rabbit HRP coupled). After incubation, membranes were washed four times 3 min in 1xPBS in order to be revealed using the ECL kit (SuperSignal West Pico Plus, Thermofisher) into an iBright imager. Western Blot from independent membranes as cells extract needed to be more concentrated in order to properly detect GyrB were used. Representative biological replicates are presented.

### Growth curve and SOS induction

Growth curves and SOS induction assays were carried out on cells either deficient in or overexpressing RecF variants to evaluate the impact on growth and increase in DNA damage of the different conditions. Strains were transformed with plasmids pQBI63 (for empty vector control) or pEAW903 (*PrecN-sfgfp*). Cell cultures were started with an inoculum 1:1000 of a saturated culture into 100 μl of LB amp containing the indicated arabinose concentration and the mix was transferred into a costar black microplate with 96 wells. The microplate was loaded into a Synergy microplate reader (Biotek) set at 37°C with shaking. The OD_600_ and the sfGFP fluorescence signal was measured every 10 min for 16 h. Finally, the fluorescence signal was calculated for each condition at each time point as followed:


$$\begin{eqnarray*}({{\rm{GFP\, cells\, pEAW903}}}){\rm{/A600\, cells\, pEAW903 - }}\nonumber\\ \left( {{\rm{sfGFP\, cells\, pQBI63}}} \right){\rm{/A600\, cells\, pQBI63}}\end{eqnarray*}$$

In the case of the growth restart assay, we only followed the OD_600_. Strains were cultivated as previously described with increased concentration of arabinose for the first 16h, then a dilution 1 to 1000 was used to inoculate a fresh culture in LB amp only. Only the OD_600_ is reported in this case.

Values indicated on graph are the mean and standard error of biological triplicates.

### Imaging of live and dead cells

We used LIVE/DEAD BacLight (Molecular Probe) to assay the viability of cells following RecF overexpression. After 16h of culture in the presence of the indicated concentration of arabinose, cells were spun down, washed and resuspended in 0.85% NaCl in order to be incubated with the adequate solution allowing the differential staining of live and dead cells as described by the manufacturer (Molecular Probe). Following the incubation, imaging of cells was carried out with inverted microscope Nikon N-STORM (100x Objective in epifluorescence mode) equipped with an ORCA FLASH 4.0 camera (512 × 512 pixel, Hamamatsu). For each experiment, 2.5 μl of cells were dispersed on a cover-slide 24 × 50 mm, No. 1.5 (Azer scientific) under agar pad 1.5% agarose. For each condition a biological triplicate was imaged. Cells were imaged in the brightfield (100 ms, 4.5V), with a DsRed filter (640 nm, 100 ms, 4.5 V) to image dead cells and with a GFP filter for live cells (488 nm, 50 ms, 4.5 V). Cells were analysed with Fiji equipped with MicrobeJ. Only individual cells in focus were selected. The number of cells for each experiment was greater than 400 cells. The percentage of dead cells was calculated as followed:


$$\begin{equation*}{\rm{Cells\, dead / }}\left( {{\rm{Cells\, alive + Cells\, dead}}} \right){\rm{ * 100}}\end{equation*}$$

For easiest differentiation of live and dead cells on the images, the LUTs of DsRed and GFP channels were respectively changed to yellow and blue before merging the three channels.

### Single-cell fluorescence microscopy imaging

Single-cell fluorescence imaging was used to study the behavior of RecF variants upon overexpression alongside labelled replication proteins of interest: DnaX-YPet and SSB-mTur2 in living cells. All imaging experiments were realized in EZ defined medium (Teknova) supplemented with 0.2% glycerol and ampicillin (EZ glycerol amp) to minimize the auto-fluorescence observed with LB media. Cultures were inoculated from overnight culture with 1:100 ratio and grown at 37°C to reach mid-log phase as described earlier (52).

Single-molecule fluorescence imaging was performed on a custom wide-field inverted microscope Nikon Ti2-E (100× Objective) equipped with a heated stage insert as previously described (77). Wide-field fluorescence imaging was realized on a 512 × 512 pixels EM-CCD camera (C9100-13, Hamamatsu epifluorescence configuration). Excitation was provided using semi-diode lasers (Sapphire LP, Coherent) of wavelengths 458 nm (41.0 mW max. output), 514 nm (150.5 mW max. output) and 568 nm (200.8 mW max. output).

To carry out the experiments, cells were loaded into a home-built flow cell as described previously (52). Cells were briefly settled (2–5 min) to allow them to stick to a silanized coverslip (treated with 3-aminopropyl triethoxysilane APTES), then fresh medium was flowed in at the rate of 50 μl/min using a syringe pump (Adelab Scientific) to both dislodge loosely associated cells and provide nutrients. During the experiment time course, freshly oxygenated medium was continuously flowed into the chamber incubated at 37°C. Cell positions were randomly determined in the bright field during the cell settling time. Time zero of the experiment corresponds to the first image capture. Directly after the capture of the first frame of each position, the flow was briefly stopped, and the EZ glycerol amp medium was substituted for the EZ glycerol amp supplemented with 0.2% arabinose. For all fluorescence imaging, an initial brightfield image of the cells was recorded (34ms exposure).

Rapid acquisitions (movies of 300 × 50 ms frames, continuous excitation with 568 nm light and collected between 610–680 nm with an ET 645/75m filter, Chroma) were realized to characterize the motions of RecF-mKate2 and RecF_K36R_mKate2 molecules before arabinose addition.

Two-color time lapse movies were recorded to visualize mKate2 fusion along with the replisome marker (DnaX-YPet) or the fluorescent fusion of single strand binding protein (SSB-mTur2) during RecF over-expression. These movies were used to determine the number of foci and the colocalization pattern of the two fluorophores used in each of those experiments. RecF-mKate2 was imaged using yellow excitation light (*λ* = 568 nm) at high intensity (76.6 W cm^−2^) and collected between 610–680 nm (ET 645/ 75m filter, Chroma, at EM gain = 150, 100 ms exposure). DnaX-YPet was imaged using green excitation (*λ* = 514 nm) at higher laser power (287.1 Wcm^−2^) and collected between 525–555 nm (ET540, 30m filter, Chroma, at EM gain = 150, 200 ms exposure). Lastly, SSB-mTur2 was imaged using green excitation (*λ* = 458 nm) at lower laser power (15.64 W cm^−2^) and collected between 468 and 495 nm (ET 485/30m filter, Chromaat EM gain = 200, 100 ms exposure). For all experiments, even when the untagged RecF was used, images were recorded in the mKate2 channel.

### Image processing

Image analysis was performed in Fiji/ImageJ, using the single-molecule biophysics plug-in (78), the Grid/Collection stitching plug-in (79), custom macros, and MATLAB. In Fiji, raw ND2 images were converted to TIF format, prior to background correct and image flattening as previously described (52). MicrobeTracker 0.937 MATLAB plug-in was used to create cell outlines as regions of interest (ROIs). Manual outlines were used to ensure that only non-overlapping, in-focus cells were selected for analysis. Cell metrics such as cell length, area, and volume were also generated utilizing this plug-in. Cell outline ROIs were then imported into Fiji to aid in the extraction of additional cell metrics including mean cell intensity, cell lengths, and foci per cell. Note that cell outlines that are occasionally imported improperly from MicrobeTracker to ImageJ (<10 for each experiment) were excluded. For all time lapse experiments, individual analysis of each replicate was realized separately, then data from separate analyses were combined. For rapid acquisition, the analysis of two sets of equivalent number of frames from a biological triplicate were analyzed separately.

Colocalization analysis of RecF with SSB-Tur2 and DnaX-mKate2 was determined using a previously described colocalization protocol (80). Briefly, focus centroid positions were obtained using Peak Fitter plug-in in Fiji/ImageJ (discoidal averaging filter of 1–4 if not mentioned or 1–3 for DnaX-YPet), then corrected for drift between fluorescence channels. Only foci with centroid positions located within 2 pixels (218 nm) of each other were classified as colocalized. Colocalization frequencies were then estimated as the ratio of colocalized foci to the total number of foci present at each time point.

Fiji tools were used to generate ROIs around RecF-mKate2 and SSB-mTur2 features under over-expression conditions. A discoidal averaging filter was first applied to stitched fluorescence channel stacks to reduce signal associated unbound protein/ cellular auto-fluorescence. A binary mask was then generated using the Yen Thresholding algorithm with a set threshold matching that used with Peak fitter. The Analyze Particles tool was then used to generate ROIs around areas of positive intensity with areas ≥3 pixel^2^. ROIs were then applied to the original flattened stitched image stack to extract feature parameters such as area and mean intensity.

### Tet recombination assay

Tet recombination assays were used to study the effect of RecF overexpression and deletion on RecA-independent template switching events. Cells transformed with plasmids carrying 101 bp Tet repeats separated by various interspace lengths (pSTL74, pSTL78 or pMB302) were grown for 16 h in LB Amp media supplemented or not by the indicated concentration of arabinose. Cultures were serially diluted in PBS by factors of ten and the appropriate dilutions were spread on LB plates supplemented with Tet and/or Amp. After 16 h incubation at 37°C, colonies were counted to determine the number of Tet events (Tet/Amp) or the c.f.u (Amp). The percentage of Tet events was determined by the frequency of events relative to the c.f.u. and expressed in percentage. A minimum of 6 biological replicates were utlilized for each strain. The significance of the difference observed was tested by t-test for each sample relative to the wild type for the same condition.

### Plasmid DNA electrophoresis

The state of plasmid DNA (intact supercoiled or smearing) purified from cells overexpressing RecF or not was tested by electrophoresis. Two stains were utilized to determine whether in some conditions, the signal is increased using a DNA stained with stronger affinity to ssDNA compared with ethidium bromide. Cells carrying pBR322 or pSTL78 were grown in LB Amp supplemented or not with 10% arabinose for 16 h. Three to six mL of cells were harvested and resuspended in 600 μl of water. Plasmids DNA were extracted using the PureYield Plasmid Miniprep System from Promega. Purified plasmid DNA of each sample was resuspended in ultrapure nuclease free water. The DNA concentration was determined by the absorbance at 260 nm using a Nanodrop. For each sample, 250 ng of DNA was resuspended in 1x GED Buffer (glycerol, EDTA and bromophenol blue) and loaded onto a 0.8% TAE agarose gel. After electrophoresis, and staining with ethidium bromide or SYBR Gold, gels were imaged using a Typhoon-FLA imager (GE Healthcare).

### Electron microscopy

The proportion of ds- versus ssDNA of plasmid DNA purified from cells overexpressing RecF or not was determined by electron microscopy. Samples for electron microscopy (EM) were obtained by spreading the reaction mixtures with the cytochrome technique described previously (81). The technique allows the differentiation of ss versus ds DNA region based on the thickness of the DNA molecules observed. The plasmid DNA samples were first purified by minipreparation extraction followed by a phenol-chloroform extraction and ethanol precipitation to ensure the high purity of the sample. Samples were dialyzed against 20 mM NaCl and 4 mM EDTA for at least 16 h at 25°C on Millipore type VM filters (0.05 μm). Then samples were spread as described (81). Imaging and photography were carried out with a TECNAI G2 12 Twin Electron Microscope (FEI Co.) equipped with a 4k × 4k Gatan Ultrascan CCD camera. Digital images of the DNA molecules were taken at ×30 000 Magnification. DNA molecules were manually counted and sorted into groups as indicated in the figures.

### Plasmid loss assay

The effect of RecF overexpression on plasmid replication in living cells was tested by plasmid loss assay. Cells deleted of the *lac* operon (EAW408, EAW1400 and EAW1401) were transformed with the pEAW1232 or pRC7 plasmids and spread on LB plates supplemented with amp, 0.5 mM IPTG, 80 μg/ml X-Gal in order to select cells carrying the plasmids. Transformed cells were then grown overnight in presence of the appropriate antibiotics before starting the experiment. Cell cultures were started in LB supplemented or not by 10% arabinose with 1:1000 ratio of the saturated culture. The number of cells carrying the pRC7 or pEAW1232 plasmids were determined at time zero and after 16 h of culture in the absence of antibiotics. Cells were serially diluted in PBS by factors of ten. The adequate dilutions were spread on XGal IPTG plates and incubated overnight at 37°C. Finally, the blue and white colonies were counted to determine the plasmid loss for each strain. Photographs of the blue/white colonies plates were kindly taken by Robin Davies from the MediaLab of the Biochemistry department of UW Madison.

### Yeast two hybrid assay

Interaction between RecF and partners was tested by Yeast-two hybrid. First, yeast *CFy7* cells were transformed as described earlier (82) with appropriate plasmids to test the interaction between RecF fused to one domain (activator AD or binding BD) and the indicated partner fused to the other encoded to the complementary plasmid pGAD or pGBD. Yeast transformants were selected on Leu-/Trp- selective drop out medium plates at 30°C. Then 4 to 5 yeast transformants were grown overnight at 30°C in liquid selective drop out medium. The optical density of overnight yeast culture was measured, and 1 mL of cells was harvested. Yeast cells were broken down by 3 cycles of freeze/thaw consisting of 3 min in liquid nitrogen and 3 min at 42°C. Cells pellets were resuspended in 1 mL of Z buffer (Na_2_HPO_4_ 60 mM, NaH_2_PO_4_ 40 mM, KCl 10 mM and MgSO_4_ 1mM) and β-galactosidase assay was carried out as described (83,84). Biological replicates of 4 or 5 experiments were realized and significative difference relative to the negative control were tested by Mann-Whitney.

### Protein preparations

The *E. coli* RecF, RecF_K36R_ and RecFmKate2 were purified as previously described (42). The *E. coli* RecR was purified using a dual-tag purification system allowing the purification of a protein of interest by a first step of maltose binding protein affinity purification, followed by the cleavage by the SUMO protease Ulp1 between the MalE-6His-Smt3 and the protein (85). This left the cleaved RecR protein without any tag as described earlier (40). For use as a control, the His-mKate2 was purified from 6L of LB amp culture of BL21 λDE3 Δ*slyD* transformed with pEAW1300 (6His-mKate2) induced at OD_600_: 0.4 with 0.5 mM IPTG and overgrew at 33˙C for 3h. Dry cell pellet of ∼20 g was flash frozen until cell resuspension. The cell pellet was resuspended overnight at 4°C in lysis buffer (50 mM Tris-Cl pH 7.5, 100 mM NaCl, 20 mM imidazole, 10% glycerol). Lysis buffer was adjusted to 75 ml. Lyzozyme 1.25 mg/ml final concentration resuspended in fresh lysis buffer was added to the cell resuspension and the mixture was stirred gently for ∼1 h. Cell resuspension was sonicated 10 times with a cycle consisting of 1min sonication, set with 0.5 s on, 0.5 s off. The cell lysate was centrifuged for 60 min at 12 000 rpm, 4˙C using JLA.16.250 Beckman Coulter rotor. Cell soup supernatant was loaded on gravity column of ∼10 mL Nickel resin pre-equilibrated in Lysis buffer. Column was washed with 3 column volume of lysis buffer prior to elution with 3 × 5 mL of elution buffer (same as lysis buffer but 500 mM Imidazole). Elution fractions were pooled and dialyzed against B buffer (50 mM Tris–Cl pH 7.5, 50 mM NaCl, 0.1 mM EDTA, 10% glycerol, 1 mM DTT). Protein was loaded on a 5 ml SP HP column and eluted on linear gradient to buffer D (same as B but 1M NaCl). Pooled fractions were dialyzed back into B buffer and flowthrough a 5 mL Q FF. Purified protein was dialyzed against RecF storage buffer and stored at −80°C.

The *E. coli* SSB protein was purified as described earlier (74). The *E. coli* replication enzymes: the replicative DNA polymerase PolIII, the clamp loader, the β-clamp unlabelled and labelled, DnaB, the RNA primase DnaG and MBP-AF647, were generous gifts from Dr S Jergic, Dr S Chang, Dr Richard Spinks and Dr N Dixon. All protein preparations were tested and found free of endo- or exonuclease activities. Protein concentrations were determined by absorbance at 280 nm using extinction coefficient of the monomeric form of each protein (if not specified otherwise), ϵ_RecF_ = 3.87 × 10^4^ M^−1^ cm^−1^, ϵ_RecFmKate_ = 6.53 × 10^4^ M^−1^ cm^−1^, ϵ_RecR_ = 5.6 × 10^3^ M^−1^ cm^−1^, ϵ_SSB_ = 2.8 × 10^4^ M^−1^ cm^−1^, ϵ_DnaG_ = 3.33 × 10^4^ M^−1^ cm^−1^, ϵ_DnaB_ = 3.08 × 10^4^ M^−1^ cm^−1^, DnaN dimer ϵ_(β-clamp)_ = 1.6 × 10^4^ M^−1^ cm^−1^, ϵ_PolIII(αϵθ)_ = 1.3 × 10^5^ M^−1^ cm^−1^, ϵ_ClampLoader(DnaX+)_ = 3.0 × 10^5^ M^−1^ cm^−1^ and His-mKate2 ϵ_HismKate2_ = 2.74 × 10^4^ M^−1^ cm^−1^.

### Far western dot blot assay

Far western blot was used to test interaction between purified RecF and other purified proteins. Interaction between RecF and identified partners was confirmed by Far-western blot, using an adapted protocol described by Walsh *et al.* (86). Three microliters of two-fold serial dilution in RecR storage buffer of protein partners RecR, BSA, DnaN and DnaG were spotted in duplicate (one use for the dot blot the other as control) to get 54 to 1.7 pmol of each protein on nitrocellulose membranes and dried at room temperature for 15 min. Membranes were blocked with 5% milk in PBS-T for 45 min at room temperature. Blocking solution was discarded, one membrane was incubated with 5% milk PBS-T containing 0.2 μM of purified RecF overnight at 4°C while the other was incubated with same volume of fresh blocking solution only (No RecF). Membranes were washed 4 times 3 min with PBS-T in order to be incubated for at least 3h with the anti-RecF antibodies (1:1000) in blocking solution. Membranes were washed 4 times 3 min with PBS-T and then incubated with the Goat-anti-Rabbit HRP antibodies in PBS-T for at least 2h. Membranes were washed 4 times 3 min with PBS before being revealed simultanously using ECL Fanto. Far western blots were carried out in biological quadruplicate and the quantification was obtained by subtracting the background signal observed in the control membrane to the signal obtained in the far-western blot membrane.

### In vitro single-molecule interaction assay of labelled protein

This method was previously described and used to confirm the lack of exchange between labelled replicative proteins post PolIII complex formation (87). We used this method to validate the interaction of labelled proteins in a mixture. Purified labelled proteins were mixed (40nM of mKate2 derivatives with 80 nM of AlexaFluor647 labeled) in 1x replicative buffer as described below (in the description of the rolling circle assay) and incubated at 37°C for 20 min. The mixed sample was then diluted 500 to 1000x in 1x replicative buffer and 50 μl was directly spread on a slide cleaned with 5M KOH and imaged immediately following the spreading. Imaging was realized with a Nikon Ti2-E (100x Objective) equipped with EM-CCD camera (C9100-13, Hamamatsu) and a heated stage insert as previously described (77). Excitation was provided using semi-diode lasers of wavelengths 568 nm (Sapphire LP, Coherent, 27 mW max. output) and 647 nm (OBIS, Coherent, 38 mW max. output). Continuous imaging of 50ms images was first carried out in the 647 channel for 1min, followed by a continuous imaging of 50ms images in the 568 channel. In *vitro* single-molecule interaction experiments were carried out at three times for RecFmKate2 and DnaNAF647 and at least twice for the control. The analysis was carried using the ImageJ/Fiji softwares. Fields of views were first flattened, then average projection of the first 150 images (647 channel) or 50 images (568 channel) were generated. Individual foci in each channel were picked using the Peak fitter with constraints of 4 pixels fit radius, a minimum distance of 3 pixels between peaks and a discoidal averaging 1_3 was applied. Tables of peaks (foci) were corrected for the offset between channels and the corrected table were used to analyze the colocalization of the foci in both ways using a maximum distance between centroid of 3 pixels. Picked foci were then ordered as not colocalized if the distance was >218nm or colocalized is the distance the distance between centroid was ≤ 218 nm. The coincidental chance of colocalization (C) between the two-colour foci was calculated as using the formula:


$$\begin{equation*}{\rm{C = }}{{\rm{A}}_{\rm{R}}} \times {\rm{ n/}}\left( {{{\rm{A}}_{{\rm{FOV}}}}} \right)\end{equation*}$$

where *A*_R_ = area of the focus, *n* = number of foci (of both 647 and 568 channels), *A*_FOV_ = area of the field of view. The distance between colocalized foci was used to sorted them by colocalization shell area, as described earlier (80).

### Conservation structure and sequence analysis

Sequence conservation of *Escherichia coli* RecF was generated using the AlphaFold structure of RecF (AF_P0A7H0_F1) with Consurf server, conservation analysis was set to 300 sequences.

### Multimers prediction structure

AlphaFold (see text) was used to predict the structure of potential multimers. AlphaFold was set up to predict the five best models of the potential multimers RecF:DnaN (1:1, 1:2 and 2:2).

### Single-molecule rolling-circle assay

Single-molecule rolling circle assay was used to study in real time *in vitro* replication in presence of the RecF variants. The dye used in the assay allows labelling of the newly synthetized double strand DNA and leave the single strand DNA unlabeled. Flow cells were prepared as described previously (88). Briefly, replication reactions were carried out in microfluidic flow cells constructed from a PDMS flow chamber placed on top of a PEG-biotin-functionalized coverslip. Once, assembled with inlet and outlet tubing the flow cell was blocked against all non-specific binding by introducing blocking buffer (50 mM Tris–HCl pH 7.6, 50 mM KCl, 2% (V/V) Tween-20).

*In vitro* single-molecule microscopy was carried out on an Eclipse Ti-E inverted microscope (Nikon, Japan) with a CFI Apo TIRF 100× oil-immersion TIRF objective (NA 1.49, Nikon, Japan). The temperature was maintained at 33°C by an electronically heated flow-cell chamber coupled to an objective heating jacket (Okolab, USA). NIS-elements was used to operate the microscope and the focus was locked through Perfect Focus System (Nikon, Japan). Images were captured using an Evolve 512 Delta EMCCD camera (Photometics, USA) with an effective pixel size of 0.16 μm. DNA molecules stained with SYTOX Orange were imaged with a CW 568-nm Sapphire LP laser (200 mW max. output), and ET600/50 emission filter (Chroma, USA) at 0.76 W/cm^2^.

Reactions were carried out in replication buffer containing 25 mM Tris–HCl, pH 7.6, 10 mM magnesium acetate, 50 mM potassium glutamate, 40 μg/ml BSA, 0.1 mM EDTA, 5 mM dithiothreitol, and 0.0025% (V/V) Tween-20. Conditions for the pre-assembly replication reactions were adapted from published methods (89–91). Solution 1 was prepared as 30 nM DnaB_6_(DnaC)_6_, 1.5 nM biotinylated circular 2 kb dsDNA substrate and 1 mM ATP in replication buffer. This was incubated at 37°C for 3 min. Solution 2 contained 50 μM dCTP and dGTP, 6 nM τ_3_δδ’χψ, 20 nM Pol III core (αϵθ) and 40 nM β_2_ in replication buffer (without dATP and dTTP). Solution 2 was added to an equal volume of solution 1 and incubated for 5 min at 37°C. This was then loaded onto the flow cell at 100 μl/min for 1 min and then 10 μl/min for 10 min. The flow cell was washed with replication buffer containing 60 μM dCTP and dGTP. Replication was initiated by flowing in the replication buffer with addition of 1 mM ATP, 250 μM NTPs, 50 μM dNTPs, 40 nM β_2_, 75 nM DnaG, 100 nM SSB_4_, and 150 nM SYTOX Orange. Where indicated 20 nM RecR, 10 or 100 nM RecF and 10 nM RecF_K36R_ were used. All *in vitro* single-molecule experiments were carried out at least three times. Image analysis was performed in FIJI, using the Single Molecule Biophysics plugins (available at https://github.com/SingleMolecule/smb-plugins).

### Primer extension assays

Primer extension assay were used as second method to study the impact of RecF addition on *in vitro* replication. Primer extension experiments were carried out as described earlier (92), with the following modifications. Reactions were carried out in 40 mM Tris–HCl pH 7.2, 20 mM magnesium chloride buffer in which fresh dithiothreitol was added at the final concentration of 10 mM. When mentioned, RecF (or RecF_K36R_) and RecR proteins were respectively added last at 40 and 80 nM before starting the reaction. Otherwise, reactions were carried out by mixing 1 mM ATP, 500 μM dNTPs, 30 nM clamp loader (τ_3_δδ’χψ), 90nMPol III cores (αϵθ), 200 nM β_2_, and 750 nM SSB_4_. For the leading lagging replication reactions 75 nM DnaG, 250 μM NTPs were also added. Reaction mixtures were kept on ice before starting the reaction. The addition of 6 ng of primed DNA was used to start the replication reactions, which were then incubated at 30°C. Aliquots of 10 μl were harvested at 0, 5 and 40 min. The reaction was stopped by the addition of the equal volume of the SDS, EDTA loading buffer prewarmed at 42°C. Finally, samples were loaded onto 0.66% Agarose TAE gel, submitted to electrophoresis and SYBR Gold stained.

### Software

ImageJ/Fiji (Microscopy) and Adobe Photoshop (Plates and gel) were used to edit the images. Brightness and contrast were uniformly adjusted for all images compared. Cells were manually outlined, to select single cells in focus with the MicrobeTracker tool in MATLAB 2013. MicrobeJ was used to automatically outline and classify Live and Dead cells (93). Excel, Origin, PRISM and MATLAB software were used to edits and analyzed the data. Figures were prepared in Adobe Illustrator.

## RESULTS

RecF was recently identified in a screen for proteins that become highly toxic upon over-expression due to an increase in DNA damage (56). This effect does not extend to over-expression of RecO or RecR. In the case of RecF, the toxicity had been noted previously and depends on its ability to hydrolyze ATP (54). The observed toxicity of RecF when the protein is present at higher-than-normal levels is the jumping off point for the current study. However, we also further examine the effects of a *recF* deletion, the effects of physiological concentrations of RecF on replisome action *in vitro*, and the interaction of RecF protein with replisome proteins DnaN and DnaG. The experiments to follow were aimed at further defining RecF effects on replication forks and more broadly to explain the phenotypic distinctions between RecF and RecO.

### Toxicity of RecF over-expression constructs

**Figure 1.**
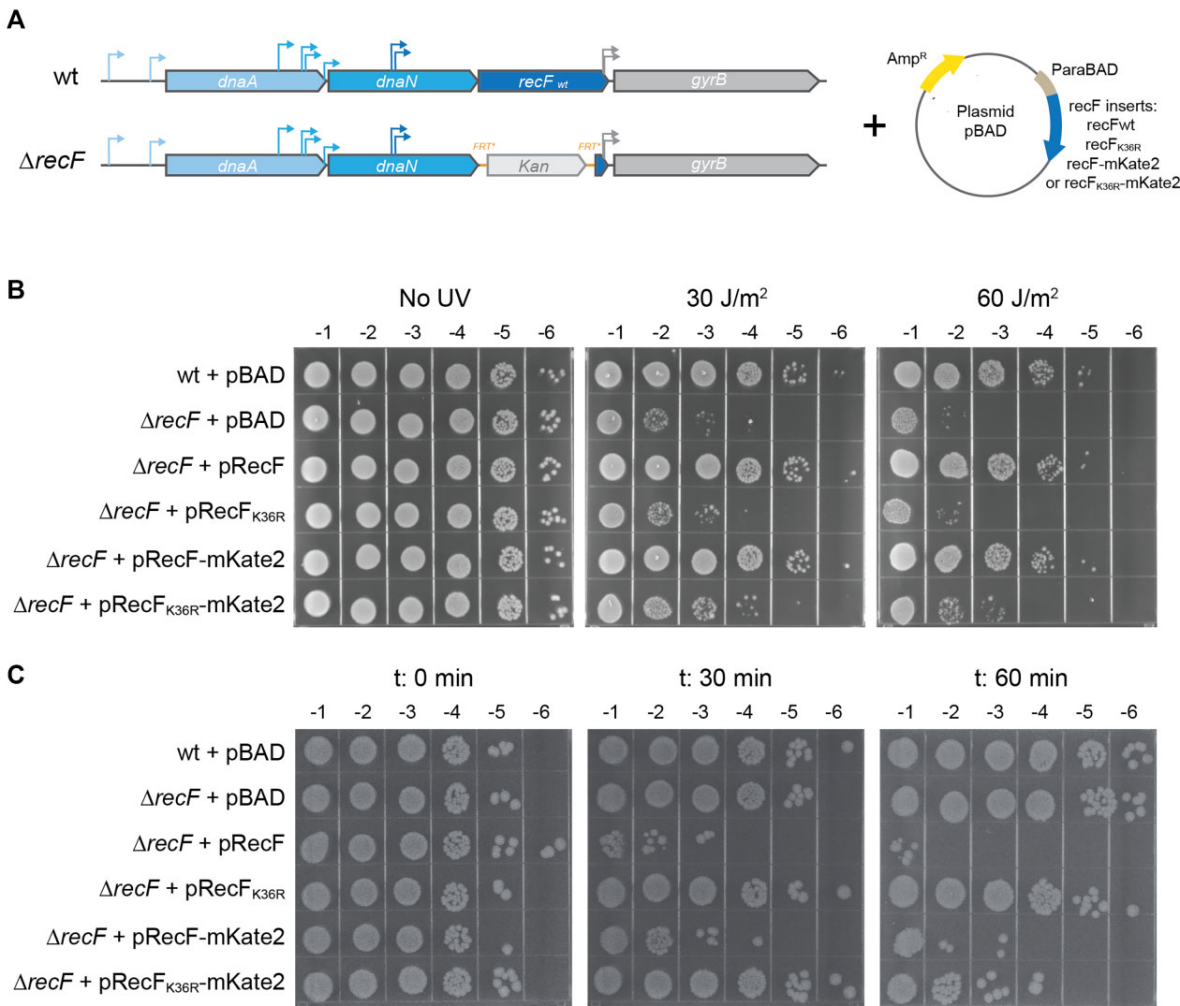
Over-expression toxicity of untagged and tagged RecF is ATPase dependent. A strain deleted of *recF* (EAW629) was transformed with plasmids encoding the different versions of RecF. The parental and EAW629 strains were also transformed with the empty vector and used as controls. (**A**) Description of the over-expression system. On the left, a representation of the *recF* locus. On the right, the representation of the pBAD vectors encoding the different RecF variants, untagged or mKate2 tagged RecF and RecF_K36R_. (**B**) The functionality of RecF-mKate2 was determined by complementation assay supported by the leaky expression of *recF* permitted in absence of arabinose. Cell cultures were serially diluted by a factor 10 down to 10^−6^ and serial dilutions were spotted in replicate on a LB amp plate. Replicates were then exposed to increased UV doses as indicated. Values on the top of the plates represent the order of the dilution. **C** RecF over-expression toxicity assay. RecF over-expression was initiated by the addition of 0.2% arabinose. Cells were serially diluted and spotted on LB amp plates at time 0, 30 and 60 min after induction.

The effects of RecF over-expression were studied at the single cell level using untagged and tagged versions of RecF. Normal functionality of a RecF-mKate2 fusion encoded at the *recF* chromosomal locus was demonstrated previously (52). Here, a pBAD vector system was used to up-regulate production of RecF wild type and mutant proteins plus tagged versions of all of these (Figure 1). To validate our over-expression tools, complementation and the toxicity of the different versions of RecF (RecF, RecF_K36R_, RecF-mKate2 and RecF_K36R_-mKate2) were tested under growth conditions adapted for single cell imaging (EZ medium containing glycerol as carbon source). Briefly, a *recF* deletion mutant strain was transformed with vectors encoding the different variants of RecF (Figure 1A). The functionality of the RecF-mKate2 construct was again validated by a UV sensitivity complementation assay, under conditions in which no arabinose was added for induction but in which leaky expression provided low levels of RecF protein (Figure 1B). The RecF-mKate2 construct was able to complement the *recF* null mutant at the same level as a similar construct expressing wild type RecF. As expected, plasmids expressing the untagged or tagged version of ATPase-dead RecF_K36R_ did not complement.

The levels of toxicity induced by over-expression of the various pBAD constructs were then analyzed after addition of 0.2% arabinose (ara) to the culture. RecF toxicity is observed 30 min after induction using an agar plate-based spot assay. The toxicity is similar for the untagged and tagged versions of the wild-type protein with a 4-log decline in survival (Figure 1C). As observed in earlier studies, the untagged RecF ATPase (K36R) mutant produced no toxicity. A partial toxicity is observed after 1h of induction for RecF_K36R_-mKate2. To ensure that the ATPase dependency is not a consequence of a difference in expression level, the expression was examined both by Coomassie gel and Western blot (Supplementary Figure S1). We noticed that the expression levels of the tagged versions are slightly lower than the untagged versions. In both cases the expression of the ATPase dead mutant (RecF_K36R_) is similar (or slightly higher) compared with the wtRecF. Altogether, these results confirm that the toxicity of the tagged RecF is comparable to that of the untagged protein upon over-expression. The results also confirm that the toxicity relies on the RecF ATPase activity and is not a nonspecific effect of the over-expression of this particular protein.

**Figure 2.**
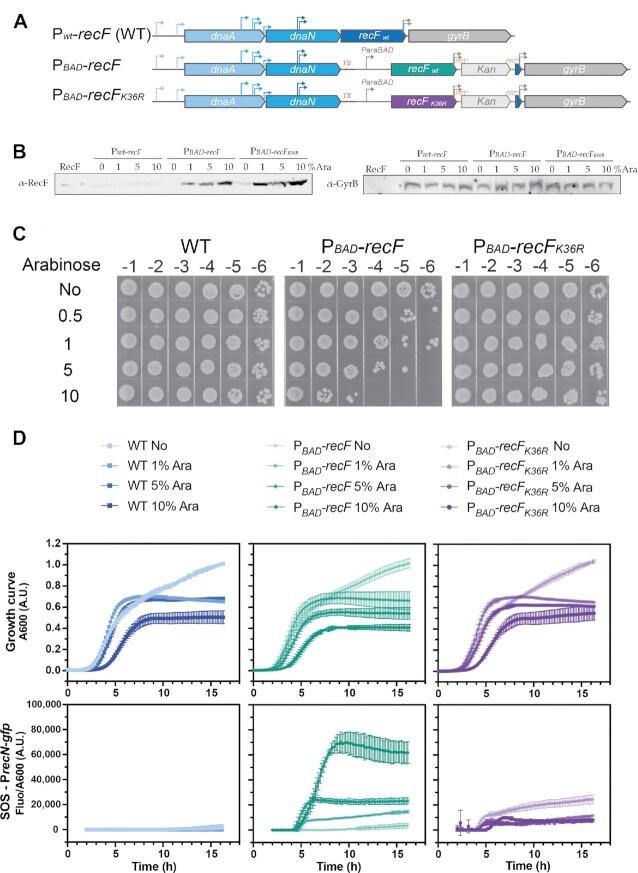
Toxicity of RecF over-expression from the chromosomal locus. Wild type RecF over-expression was realized from a chromosomal construct in which the wild type promoter at the normal *recF* locus was substituted by the *araBAD* promoter. (**A**) Schematic description of the chromosomal over-expression strains. The regulation of *recF* (EAW1130) or *recF_K36R_* (EAW1148) genes is placed under the control of the *araBAD* promoter as a termination sequence was added after *dnaN*, the 3′end of *recF* encoding *gyrB* promoter has been duplicated downstream of the Kan cassette. The parental strain carrying the native promoter was used as control. (**B**) Expression of RecF and GyrB following 6h incubation with increased concentration of arabinose was determined by Western-Blot anti-RecF and anti-GyrB. Similar samples were loaded but the immunoblots are from different membranes. Representative membranes of biological triplicate were used. (**C**) Determination of the arabinose concentration required to detect RecF over-expression toxicity. Cells were serially diluted and spotted on LB plates after culturing cells for 16 h in presence of the indicated concentration of arabinose. The addition of 10% arabinose caused a dramatic toxicity for those strains over-expressing RecF, whereas no toxicity was observed for the parental or the inducible RecF_K36R_. **(D**) SOS response was monitored over time after RecF over-expression using a P*recN-sfgfp* reporter. The fluorescence signal was plotted relative to absorbance at 600 nm under over-expression. Signal started to be recorded 2 h after the inoculation at the 1:1000 ratio in presence of absence of arabinose. RecF over-expression led to a significant increase in the SOS response. Values represented are the mean ± SD of biological triplicates.

To further investigate the effect of RecF over-expression, we constructed a series of strains in which over-expression was mediated from the chromosomal *recF* locus. The native *recF* gene is located in a complex operonic structure composed of *dnaA-dnaN-recF-gyrB* regulated by multiple promoters distributed throughout the operon (63,64) (Figure 2). The positioning of *recF* within an operon dominated by genes expressing proteins involved in replication has always been a curiosity. Notably, *recR* is also found in an operonic structure dominated by *dnaX-ybaB* (genes encoding respectively two subunits of the clamp loader and a small DNA binding protein) (94). Of course, operon placement is not determinative. The *gyrB* gene is predominantly expressed as a single gene utilizing a promoter located in the 3′ end of *recF* (64). Due to the complex regulation of this operon, we designed over-expression constructs for which a transcription termination sequence followed by the promoter of the *ara*BAD operon (P_BAD_) was inserted at the locus in front of the start codon (ATG) of the gene encoding *recF* (or *recF_K36R_*). After the *recF* stop, a Kan cassette followed by a duplication of the 3′end of *recF* carrying the *gyrB* promoter region was introduced (Figure 2A). This arrangement preserved normal expression of the *gyrB* gene (Figure 2B), unaffected by subsequent arabinose additions. A strain with the native *recF* gene, in its normal operon context, was used as control in all the experiments carried out with these over-expression constructs. Reasoning that the chromosomal construct would produce lower RecF protein levels, we carried out arabinose titration to determine the concentration exhibiting a toxicity similar to the plasmid construct in Figure 1 (Figure 2 and Supplementary Figure S2). To determine the toxicity of RecF over-expression, we followed population growth with both OD_600_ and colony forming unit (c.f.u.) measurements. Whereas almost no change in OD was observed, the c.f.u. decreased after arabinose addition. In the absence of arabinose, no toxicity was detected on plates (Figure 2 and Supplementary Figure S2). About 1 and 1.5 log loss of survival was observed at 0.5 and 1% ara but the toxicity drastically increased to ∼2 and 3.5 log loss of survival when 5 or 10% ara were added, respectively. The amount of RecF leading to toxicity (1 log loss of survival) corresponds to an increase of RecF ∼14× (Supplementary Figure S2) which is estimated to be ∼700 molecules per cell compared to the initial level of 50 molecules per cell. No toxicity was observed for RecF_K36R_ at the same concentrations of arabinose.

Western blots demonstrated that the production level of RecF was similar (or somewhat lower) to that of RecF_K36R_ under these over-expression conditions (Supplementary Figure S2). Moreover, western blot anti-RecF carried out at different times suggested that the maximum production of RecF level is reached at 6h with the higher dose of 10% ara (Supplementary Figure S2). We further used western-Blot anti-RecF to estimate the number of RecF per cell after 16h of culture (Supplementary Figure S2). To determine whether the difference observed between absorbance and c.f.u. was due to the effect of filamentation on absorbance readings or to the inability of cells to resume growth after RecF over-expression, we performed live and dead single-cell imaging and followed the growth restart of cells which previously over-expressed RecF (Supplementary Figures 3 and 4). Live and dead single cell imaging revealed an increase in cell length and cell death upon RecF over-expression (Supplementary Figure S3). The maximum cell death detected is about 30% after addition of 10% arabinose, which is expected to be higher based on the 0.01% survival (c.f.u.) of a culture which experienced almost no decrease in absorbance. However, the growth restart assay (Supplementary Figure S4) revealed a delay of about 4 h for cells which previously experienced RecF over-expression. Overall, the results of RecF over-expression from the chromosome replicate the previous observations of RecF over-expression toxicity from a plasmid and further suggest that RecF over-expression toxicity is due in large measure to a flaw in growth restart when RecF is over-expressed.

Two sets of published studies differ in the levels of SOS induction observed as a result of RecF over-expression (54–56). Sandler *et al.* (54,55) observed a decrease in SOS induction following UV or mitomycin C exposure, monitoring a short 2 h window following RecF over-expression (using a temperature inducible plasmid system). Conversely, Xia *et al.* (56) detected an increase in SOS induction after 24h of RecF over-expression (using an IPTG inducible plasmid system). In the present study, strains containing chromosomally expressed RecF or RecF variant transformed with a P*recN-sfgfp* fusion expressed on plasmid were used to assay SOS induction. We first analysed the SOS level upon RecF over-expression, both alone and combined with UV stress to address the apparent difference in SOS induction previously observed (Supplementary Figure S5). In the first 2h following the over-expression a mild delay in SOS induction was observed for the RecF over-expression strains. Consistent with Sandler's findings, the level of SOS induction was relatively low at early times (54,55). Later, the SOS response became prominent (Supplementary Figure S5), as seen by Xia *et al.* (56). Therefore, we propose that the difference between previous studies is likely primarily due to the timing of the SOS experiments and possibly also to a difference in the RecF over-expression induction system utilized.

We further analysed the effect of RecF over-expression using increased arabinose concentration (in the absence of UV) by monitoring both the fluorescence derived from the SOS-induced GFP and overall cell growth. The mean fluorescence observed for cells with the native *recF* promoter varies from 0 to a maximum of 3000 A.U. after 16h with arabinose (Figure 2D). In the absence of arabinose, the RecF over-expression construct with the P_BAD_ promoter, exhibited similar results. However, under increased over-expression conditions of 10% arabinose, the SOS-mediated GFP expression strongly increased >20 times after 9 h. For the ATPase dead RecF_K36R_ inducible construct, in the absence of inducer, a modest SOS signal is observed after 16h. This small induction of the ATPase dead mutant mimics the SOS level observed with the same fusion upon loss of the *recF* gene in absence of exogenous stress (52). In the presence of arabinose, this modest level of SOS induction decreased to background levels seen in experiments with the cells carrying the native *recF* promoter.

Finally, we tested the SOS induction in the first 16 h of RecF over-expression with 10% arabinose in homologous recombination deficient mutant strains, Δ*recA*, Δ*recB*, Δ*recO* or Δ*recR* (Supplementary Figure S6). Upon RecF over-expression, no change in SOS induction was observed in the Δ*recR* strain. Significant delays in SOS induction were observed for Δ*recO* and Δ*recB* while no induction was observed in the Δ*recA* negative control. Altogether, these results confirm the toxicity of RecF ATPase over-expression and reveals its correlation with both SOS induction and a defective cell capacity to return to growth. The results suggest an increase in DNA damage and generation of ssDNA that is dependent on RecF ATPase.

### RecF over-expression increases replisome dissociation

**Figure 3.**
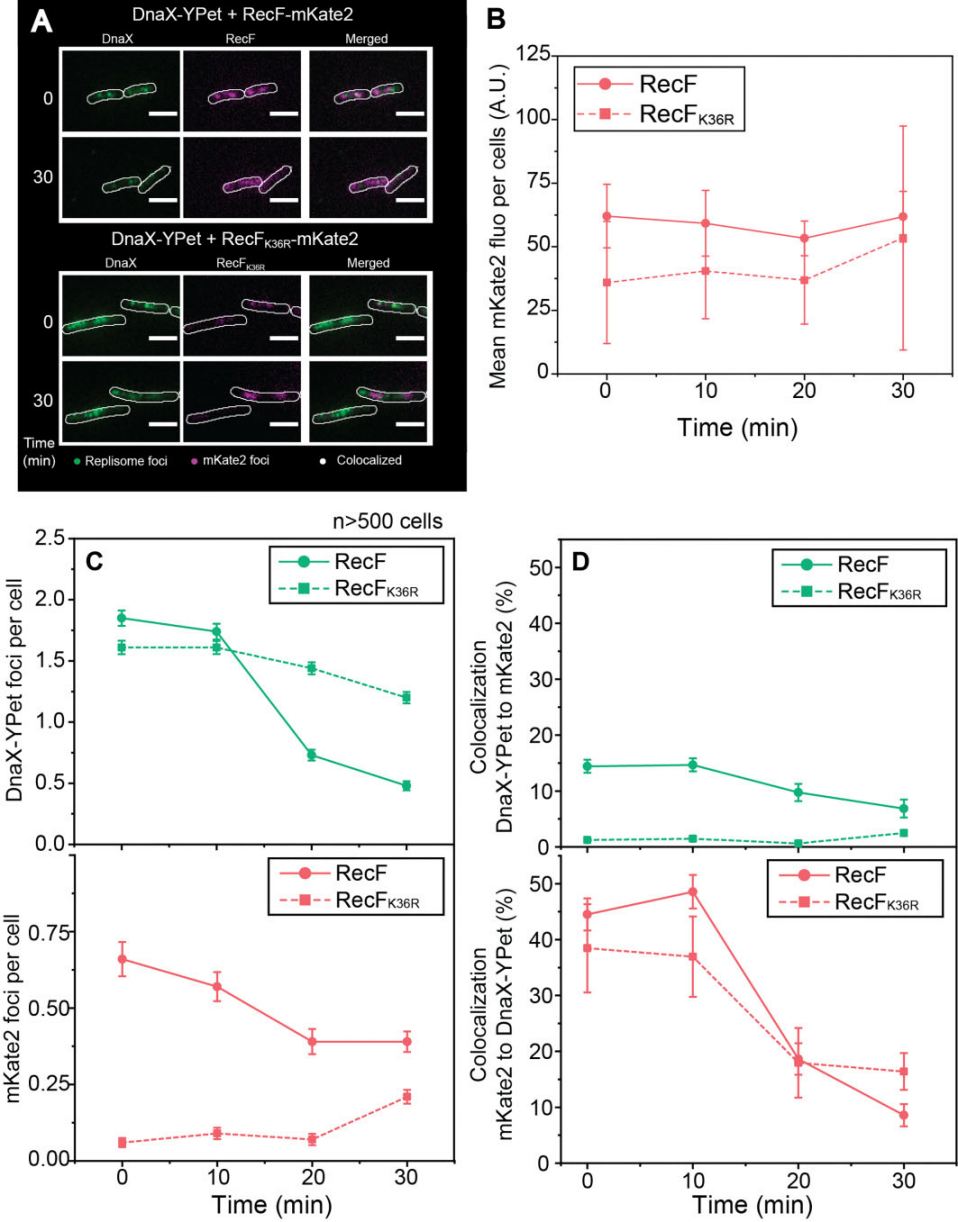
RecF-mKate2 over-expression increases replisome dissociation. The effect of RecF-mKate2 over-expression on replisome stability was determined by two-color single cell imaging. Strains used are deleted the *recF* gene, expressed a fluorescently tagged version of the clamp loader (DnaX-YPet), and carried a vector encoding the mKate2 tagged versions of RecF. Cells were loaded into home-built flow chamber incubated at 37 ºC and imaged as described in the method section. (**A**) Colocalization imaging between mKate2 (RecF or RecF_K36R_) and the replisome (DnaX-YPet). Images were obtained in the single channels 568 nm (mKate2) and 514 nm (DnaX-YPet), then merged. Imaging of single cells before and 30 min after arabinose addition. (**B**) Evolution of the mKate2 fluorescence signal per cell over the 30 min of over-expression. The values represented are the mean ± SEM, at time 0, 10, 20 and 30 min after induction, with *n* > 500 cells for each strain. (**C**) Number of replisome and mKate2 (RecF) foci detected during the 30 min following the over-expression. (**D**) Colocalization percentage during the 30 min of over-expression of one fluorophore foci relative to the other and vice versa. The values represented are the mean value ± SEM.

Reasoning that RecF acts in some manner near the replisome, we investigated the effect of RecF ATPase over-expression on replisome stability *in vivo* using a fluorescence-based imaging approach. We set up a two-color imaging strain carrying a replisome marker (DnaX-YPet) along with RecF-mKate2 expressed from the *araBAD* promoter with addition of 0.2% arabinose (Figure 3). DnaX-YPet and RecF-mKate2 signals were respectively characterized at 514 and 568 nm by imaging individual cells and foci therein. As high levels of mKate2 could be excited at 514 nm and therefore creates a possible channel overlap of signal, we first determined if any artifactual mKate2 signal (bleed through) could be observed on the YPet channel (Supplementary Figure S7). Control imaging was carried out with one-color strains expressing only RecF-mKate2. Images were recorded in both the YPet and mKate2 channels under over-expression conditions. At time 0, no artifactual signal was detected in the YPet channel. However, an artifactual signal in the YPet channel appeared after 60 min of RecF-mKate2 over-expression. Based on this result, the imaging time-lapse of DnaX-YPet in the two-color strains was limited to the first 30 min after induction of RecF-mKate2/RecF_K36R_-mKate2.

No significant increase of mKate2 cellular fluorescence was observed in two-color strains expressing either RecF-mKate2 or RecF_K36R_-mKate2 30 min after arabinose induction. Nonetheless, previous western blots revealed increased RecF-mKate2 production during that time-period. Though at first glance this observation appears contradictory, we attributed these observational differences to limitations of the mKate2 fluorophore. The fluorophore mKate2 was previously determined to have a half maturation time of roughly 20 min (95). Thus, during our observational window of 30 min, it is reasonable to assume that the maturation lag of mKate2 fluorophores could obscure the observation of newly created RecF-mKate2 protein. Therefore, most of the RecFmKate2 foci observed likely result from the basal leaky expression and limit us to track only a part of the RecFmKate2 pool. Importantly, RecFmKate2 overexpression results revealed a similar toxicity relative to RecF alone, suggesting that maturation of the mKate2 doesn’t affect the RecF portion of the fusion. Next, we determined the number of mKate2 and replisome foci per cell (Figure 3C and Supplementary Figure S8). Before induction, strains expressing either RecF-mKate2 or RecF_K36R_-mKate2 exhibited similar numbers of replisome foci per cell ∼1.7 (top panel). However, the number of mKate2 foci (RecF) was significantly smaller for the ATPase dead RecF_K36R_ mutant with 0.06 ± 0.02 (RecF_K36R_-mKate2) versus 0.66 ± 0.06 (RecF-mKate2) (bottom panel). This suggests that the ATPase function may be needed for RecF dimerization and DNA binding *in vivo*. A similar result was obtained when acquiring rapid-acquisition movies rather than time-lapse series (Supplementary Figure S8). After arabinose addition, the number of RecF-mKate2 foci slightly decreases after 10 min of induction whereas it increases to 0.21 ± 0.02 for RecF_K36R_-mKate2 (Figure 3C bottom panel and Supplementary Figure S9). The use of a replisome marker (DnaX-YPet) revealed that RecF over-expression correlates with a sharp decline in replisome foci, beginning at 10 min after induction and decreasing further from 20 to 30 min (Figure 3C top panel). Over 70% of the visible replisome foci disappear upon over-expression of the RecF-mKate2. A much more modest decline is observed upon over-expression of the ATPase deficient RecF_K36R_-mKate2.

The proximity of the RecF-mKate2 and RecF_K36R_-mKate2 foci to the replisome was further analyzed by examining histograms of pairwise-colocalization distances. To account for the fact that short distances are sampled less frequently in these types of radial-search measurements, the histograms of colocalized foci were binned by area shells, as opposed to linear distances (52). The number of mKate2/YPet colocalization counts in close proximity was higher for RecF-mKate2 at time 0 and remained high for the first 10 min (Supplementary Figure S9). During the first 10 min after arabinose addition, 14.43 ± 1.16% of replisome foci colocalized with RecF-mKate2) (Figure 3D). This colocalization declined within 30 min coinciding with a decline in the total number of replisome foci. The colocalization is more substantial if considered from the standpoint of the less common RecF foci. For both RecF variants, a significant level (∼40%) of the RecF foci colocalized with replisome foci (Figure 3D), although the numbers of RecF_K36R_-mKate2 foci were low. There were few mKate2 foci to follow, very few replisome foci included them <2% for the first 10 min and this number further declined within 30 min. Throughout the 30 min of the experiment, RecF_K36R_-mKate2 foci remained relatively rare. However, we noticed that when RecF_K36R_-mKate2 formed foci, the proximity to the replisome was not different from that seen for RecF-mKate2. Thus, RecF focus formation exhibits a strong dependence on the RecF ATPase activity, whereas the proximity of RecF foci to the replisome does not.

**Figure 4.**
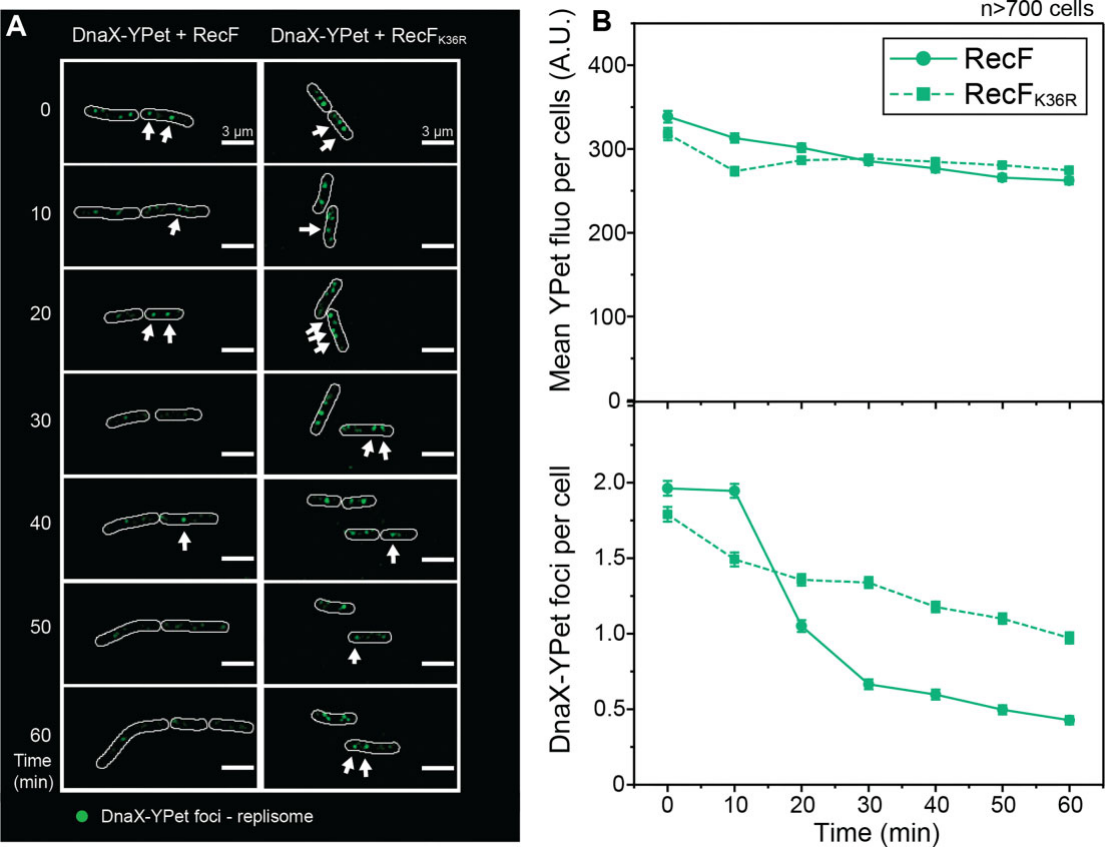
Over-expression of untagged (wild type) RecF also increases replisome dissociation The integrity of the replisome upon RecF over-expression was determined over a period of 60 min using single-color single cell imaging. Cells expressing the tagged clamp loader (DnaX-YPet), deleted of the chromosomal *recF*, and carrying the pBAD vector encoding untagged versions of RecF were imaged, *n* > 700 cells. (**A**) Single cell time-lapse image of the *E. coli* replisome upon RecF over-expression. A discoidal filter has been applied on the image with Fiji. (**B**) Analysis of the YPet fluorophore in the cell. The upper panel represents the mean YPet fluorescence per cell over the 60 min of over-expression, the mean fluorescence was similar for both strains. The lower panel represents the number of replisome foci over time. The number of replisome foci decreased over time with a stronger effect for RecF over-expression. The values represented are the mean ± SEM.

To confirm some of the key observations over a longer period of time, we next imaged the single-color *dnaX-YPet* strains, over-expressing the dark (untagged) versions of RecF and RecF_K36R_ (Figure 4). Overall patterns remained the same. The relative total YPet concentration per cell was similar for both strains during the 60 min observation window, suggesting similar concentrations of replisome proteins (at least DnaX). RecF over-expression again produced a significant decrease (>70%) in replisome foci (Figure 4 and Supplementary Figure S10). The number of replisome foci observed at 30 min was similar to that seen in the previous imaging of the two-color strains, for untagged RecF and RecF_K36R_ respectively. The decline continued from 30 to 60 min post-induction of the experiment for both constructs, reaching 0.43 ± 0.03 replisome foci for RecF and 0.97 ± 0.04 for RecF_K36R_. RecF protein over-expression thus leads to replisome uncoupling and transient destabilization in a reaction that is largely dependent on an intact RecF ATPase activity.

**Figure 5.**
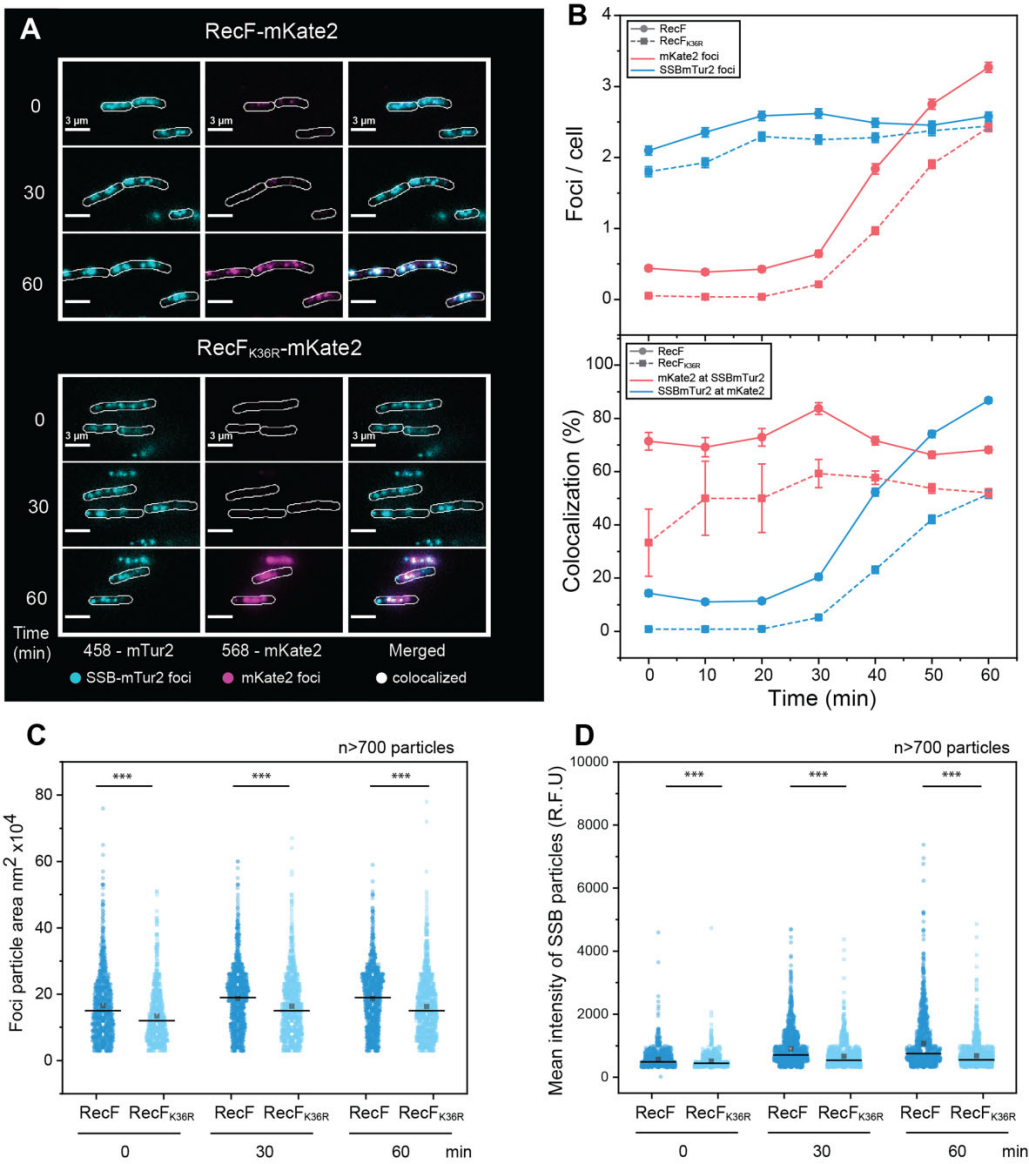
RecF-mKate2 over-expression increases the size and intensity of SSB-mtur2 features. Imaging of the single-stranded DNA regions in the cell carrying a labelled SSB-mTur2 were realized upon RecF-mKate2 over-expression. Cells deleted of wild type *recF*, expressing a chromosomal SSB-mTur2, and carrying the pBAD vector encoding the mKate2 versions of RecF were imaged. (**A**) Colocalization imaging of the ssDNA regions upon RecF-mKate2 over-expression. Images were taken in the 568 (mKate2) and 458 (mTur2) channels and then merged. (**B**) SSB foci are represented in blue and the mKate2 foci are represented in pink, strain expressing RecF-mKate2 is represented by circles on a continuous line and the strain expressing RecF_K36R_-mKate2 is represented by squares and a dashed line. The values represented are the mean ± SEM. Representations over time of the number of foci per cell (upper panel). Colocalization analysis of SSB-mTur2 and mKate2 (bottom panel). SSB-mTur2 was more often found colocalized with RecF-mKate2 than RecF_K36R_-mKate2. The colocalization increases after 30 min with a greater effect for RecF-mKate2. (**C**, **D**) Analysis of the SSB particles (particle = continuous region of individual or overlapping SSB foci). The area **(C)**, and the intensity **(D)**, of more than 700 particles were determined at time 0, 30 and 60 min and are represented as a scatter plot.

In principle, replisome dissociation could have several different effects on the local binding of SSB: (i) a reduction caused by RecA protein loading onto the ssDNA region mediated by RecOR on the abandoned fork, with coincident SSB removal (96,97); (ii) a static presence of SSB if the replication is resumed by the replication restart proteins without further DNA unwinding or (iii) an increase in the ssDNA SSB coated region, if the abandoned replication fork is further processed by helicases or if post-replication gaps are formed. To explore these possibilities and follow the fate of SSB, we used a new SSB-mTur2 visualization tool developed by Keck and coworkers (Figure 5) (67) to image the ssDNA regions (i.e. replisome and gap). Unlike other SSB fusions studied to date, *E. coli* cells grow normally when SSB-mTur2 is the only SSB expressed. Controlling for possible channel overlap with mKate2 (RecF) (Supplementary Figure S7), we detected no artifactual foci in the mTur2 channel (458 nm). Strains expressing chromosomal SSB-mTur2 alongside of RecF-mKate2 or RecF_K36R_-mKate2 were imaged for 60 min after arabinose addition (Figure 5). In agreement with the number of replisome foci observed under the same growth conditions, the number of SSB-mTur2 foci before induction was around 2 foci per cell for both strains. In contrast to the replisome, the number of SSB foci increased slightly after induction. As might be expected, this suggests that a region of ssDNA remained whether the replisome was present or not. When the ATPase dead RecF_K36R_-mKate2 was expressed, a similar small increase in SSB foci was observed to reach ∼2.5 after 60 min of induction. Prior to induction, low levels of RecF-mKate2 foci (about 0.5 per cell) and very few RecF_K36R_-mKate2 foci were present. Both RecF-mKate2 and RecF_K36R_-mKate2 foci increased upon induction, mainly after 30 min as the newly synthesized mKate2 fluorophores matured. RecF-mKate2 strongly colocalized to the SSB foci. Although RecF_K36R_-mKate2 exhibited many fewer foci after 30 min, these also colocalized well to the SSB foci. Even before induction, 71.4 ± 3.3% of RecF-mKate2 foci colocalized with SSB-mTur2 foci. The colocalization slightly increased at 30 min, and then returned to the initial colocalization level. Colocalization of the detectable RecF_K36R_-mKate2 foci with SSB foci was significant (33.3 ± 12.6%) but lower than with RecF-mKate2 protein. In contrast to the replisome colocalization analysis, this implies that RecF might bind more often near non-replisomal SSB foci than RecF_K36R_. Upon induction, as RecF_K36R_-mKate2 increased and formed more visible foci after the 30 min mark, its colocalization with SSB-mTur2 foci reached a plateau between 50 and 60%. In the reciprocal analysis, 14.3 ± 1.4% of the SSB-mTur2 foci contained RecF-mKate2 foci prior to induction and less than 1% of the SSB-mTur2 foci colocalized with the much smaller number of RecF_K36R_-mKate2 foci. After induction, the fraction of SSB foci colocalizing with either RecF variant increased substantially with maturation of mKate2 fluorophores after the 30 min mark. Over 89% of the SSB foci colocalized with RecF-mKate2 after 60 min and just under 50% in the strains expressing RecF_K36R_-mKate2, perhaps partially due to the presence of fewer RecF_K36R_-mKate2 foci.

Finally, the analysis of the SSB foci characteristics analyzed as particles in Fiji revealed that RecF over-expression increased the size and brightness of the particles in a manner that was much more pronounced for strains expressing the RecF-mKate2 protein (Figure 5 CD). The size and brightness of RecF foci (particles) also increased after 30 min, although this may simply reflect the slow maturation of the mKate2 fluorophore (Supplementary Figure S11). Altogether, these data show that RecF over-expression does not greatly affect colocalization with SSB foci but it does affect SSB feature size and brightness as well as RecF DNA binding. These results again indicate an increase in ssDNA generated by RecF over-expression, correlating with a decline in cellular replisome numbers.

### RecF over-expression stimulates repeat deletion events associated with post-replication gaps

**Figure 6.**
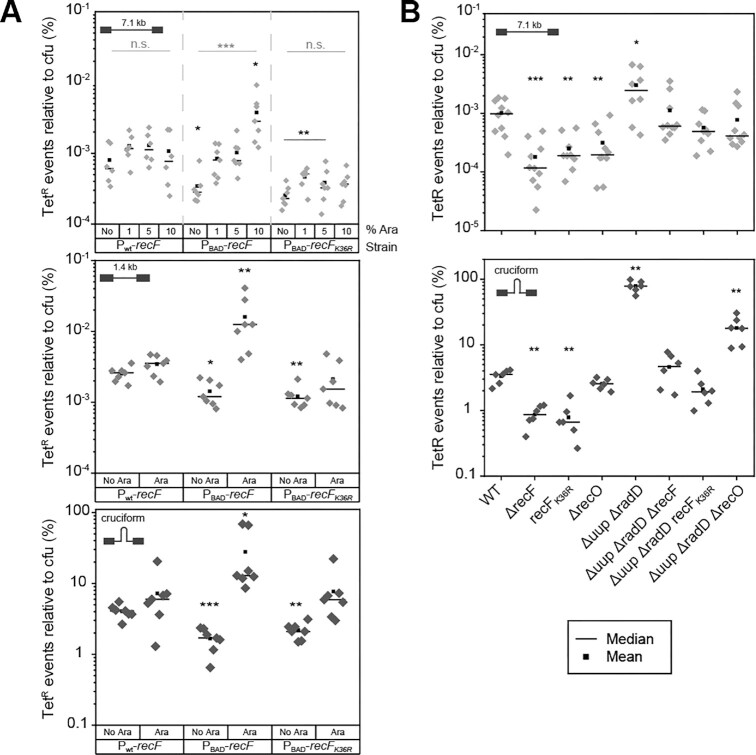
RecF over-expression stimulates Tet recombination events through its ATPase activity and *recF* deletion decreases their occurence. The effect of RecF ATPase over-expression on Tet repeat recombination events (Tet^R^) was examined using a plasmid-based assay. Strains carrying the indicated plasmid were grown 16 h in the presence of the indicated concentration of arabinose (or 10% if not specified) and plated on Amp and Tet/Amp plates to determine the recombination frequency. The percentage of Tet^R^ recombination events relative to the total c.f.u. of at least six biological replicates is represented as a dot plot, for each strain. The mean and the median are respectively represented by a square or a line, respectively. (**A**). Tet deletions events was tested upon increased concentration of arabinose. Wild type (P*_wt_-recF*), EAW1130 (P*_BAD_-recF*) and EAW1148 (P*_BAD_-recF_K36R_*) strains were transformed with the pSTL78 (upper panel), pSTL74 (medium panel) or pMB302 (lower panel). (**B**). The requirement for RecF ATPase for the Tet repeats recombination events was tested for deletion and point mutation strains using a plasmid-based assay. The wt, EAW629 (Δ*recF*), EAW1190 *(recF_K36R_)*, EAW114 (Δ*recO*), ZJR04 (Δ*radD* Δ*uup*), EAW1063 (Δ*radD* Δ*uup* Δ*recF*), CJH0115 (Δ*radD* Δ*uup recF_K36R_*), and EAW1064 (Δ*radD* Δ*uup* Δ*recO*), strains were transformed with the pSTL78 (upper panel) or pMB302 (lower panel). Significant difference compared to the parental strain (wt) was tested by Mann-Whitney and are indicated in black (* for *P* = 0.05, ** for *P* = 0.005 or *** for *P* = 0.0005), an additional Kruskal–Wallis test was realized to compare the effect of increased concentration of arabinose for each strains and significance is indicated in grey.

The colocalization behavior of RecF associates the protein with both replisomes and gaps. If RecF over-expression is leading to larger numbers of post-replication gaps, then it might also lead to an increase in recombination events linked to those gaps. This experiment utilized an assay developed by Lovett and collaborators (15,71,72), measuring deletion events between nearby short (101 bp) direct repeats on plasmids that are largely RecA-independent and strongly associated with post-replication gaps (71,72). The deletion events create tetracycline resistance and are readily selected for. We carried out experiments with three plasmids harbouring variously sized regions between the two tet repeats of 101bp homology (1.4, 7.1 kb and cruciform formed by a palindrome of 110 bp) (Figure 6A). Recombination events between repeats were detected by plating after 16h of culture following induction by arabinose addition. No protein tags were present on the RecF or RecF_K36R_ proteins. For all of the assayed plasmids, significant increases in deletion events were seen when the wild type RecF protein was induced. Increases were minimal or absent when the RecF_K36R_ protein was induced or when the wild type *recF* promoter (unresponsive to arabinose) was used.

To expand the correlation and examine conditions that did not involve RecF over-expression, the assay was then repeated in strains lacking RecF protein (Figure 6B). In agreement with the observations of Lovett and co-workers on intermolecular recombination events between tet repeats greater than 50 bp (14), a *recF* deletion in all cases decreased the frequency of the events. The same result was obtained for strains expressing the ATPase dead RecF_K36R_. When using a plasmid in which the repeats are separated by 7.1 kb (where the background rate of deletion is very low), a deletion of the *recO* gene also caused a measurable decline in deletion frequency. When a plasmid was used that had much less DNA (110 bp with a long palindrome) separating the repeats, the loss of RecF function again caused a decline in deletion frequency (Figure 6B). For this latter deletion substrate, where deletion frequencies are much higher, deletion of *recO* did not produce a decline in the manner of Δ*recF*. In an attempt to confirm the *recF* and *recO* patterns, a similar set of experiments was carried out in a background in which the functions of the Uup and RadD proteins are missing. Eliminating these two genes has the effect of enhancing the recombination signal, as deletion of those two genes produces a synergistic increase in these types of RecA-independent deletion events (8). The patterns seen with *recF* and *recO* were confirmed with these strains. As most of this recombination is RecA-independent, the effect of the *recF* deletion indicates that RecF is involved, at least in part, in a process that does not involve RecA protein loading into the gap. The more modest effects of the *recO* deletion are consistent with the role of RecO in RecA loading. In general, these experiments indicate that deletion events associated with post-replication gaps increase when RecF is over-expressed in an ATPase-dependent fashion and decline when RecF is not present. The RecF over-expression appears to be associated with an increase in gap formation and/or an increase in gap size that provides fertile ground for RecA-independent recombination.

### RecF over-expression increases damage and ssDNA formation on plasmid DNA

**Figure 7.**
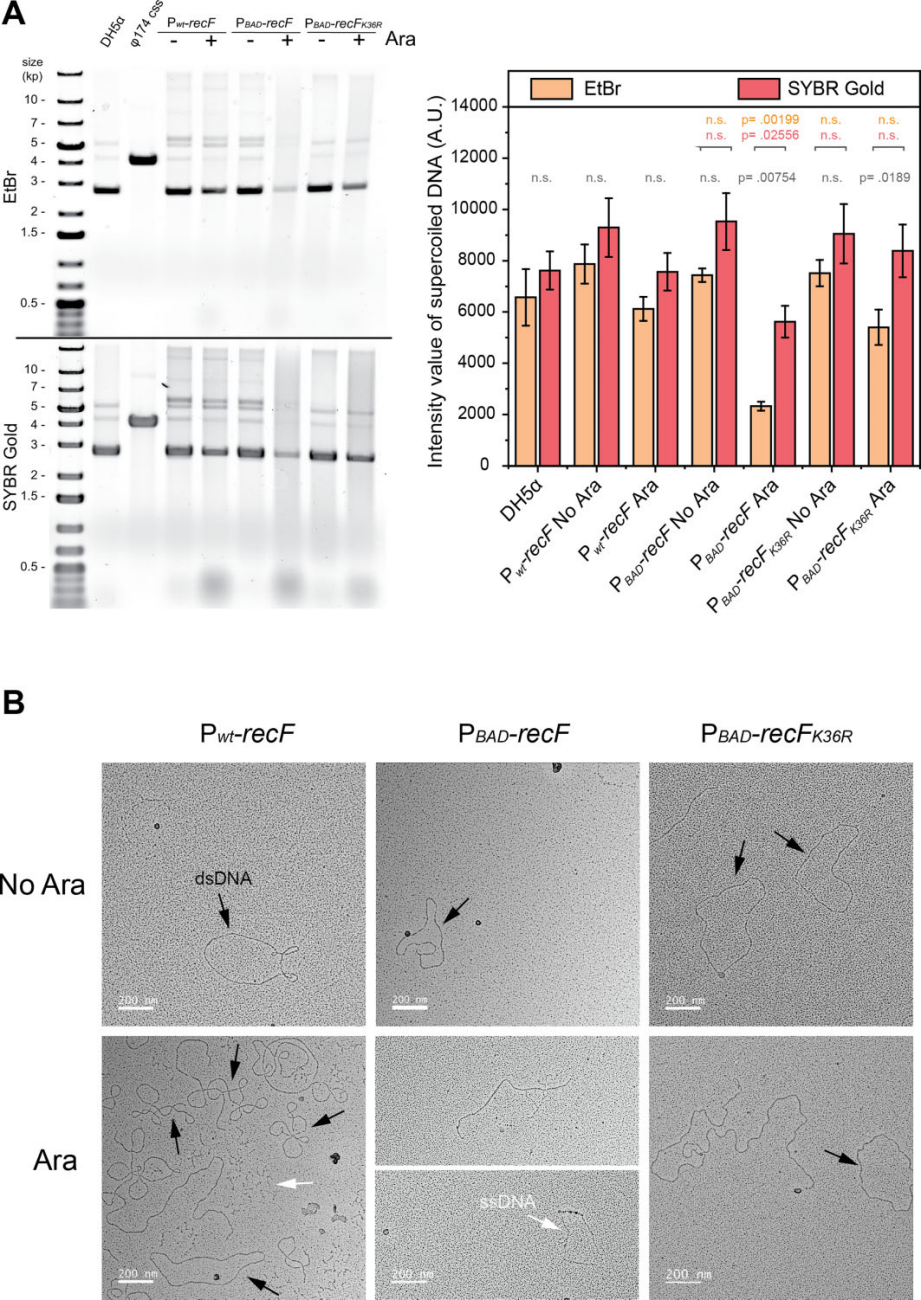
RecF over-expression increases damage and ssDNA formation on plasmid DNA. The effect of RecF ATPase over-expression on plasmid integrity was determined by quantitative electrophoresis and electron microscopy. Cells were grown for 16 h in presence or absence of 10% arabinose. (**A**) Plasmids isolated from cells as mentioned. Purified plasmid DNA (250 ng) was loaded onto two identical 1% agarose gels. After electrophoresis DNA was EtBr or SYBR Gold stained. The upper panel represent the gel images of representative experiments and lower panel represent the average of the raw intensity signal detected for the major band for a biological triplicate ±SD. The *P* values of significant differences between staining methods are indicated in grey and differences relative to the wt strain in the same conditions are indicated respectively in orange and pink for EtBr and SYBR Gold. (**B**) Electron microscopy images of pBR322 purified DNA using the cytochrome C spreading method. The cytochrome C spreading allowed the differentiation between the dsDNA (black arrow) and the ssDNA (white arrow). RecF over-expression led to an apparent increase in observed ssDNA, concomitant to an almost complete loss of the circular dsDNA in the plasmid preps.

We reasoned that an effect on replisome stability, along with an increase in gap formation, might be especially detrimental to small replicons (plasmids) and might be reflected in an increase in DNA damage and plasmid loss. We specifically examined the effect of RecF over-expression on the stability of the plasmid pBR322 (70). We first took strains expressing RecF from its wild type promoter on the chromosome, as well as RecF and RecF_K36R_ expressed from the pBAD promoter. Before and after addition of 10% arabinose to induce RecF or RecF_K36R_ expression for 16h, plasmid DNA was purified, visualized and analyzed. The toxicity of RecF over-expression for the strains carrying the plasmid was controlled using an agar plate-based spot assay (Supplementary Figure S12). After plasmid purification by standard mini-preparation carried out in the same manner for all strains, the DNA concentration was determined by the absorbance at 260 nm. In each case, 250 ng of DNA was loaded on two identical agarose gels (Figure 7). Following electrophoresis, identical gels were stained either by ethidium bromide or SYBR Gold. SYBR Gold is a more sensitive stain able to detect lower concentration DNA and single strand DNA. For most samples, the majority of the 250 ng of pBR322 DNA appeared to be supercoiled with both stains (Figure 7A). The exception was the DNA purified from cells over-expressing RecF. In that case, the majority of the DNA is spread into a smear. Unlike the other samples, a larger amount of DNA was detected by SYBR Gold staining, thus suggesting a potential increase in ssDNA. A similar smearing was observed for a larger plasmid (pSTL78; 7.1 kb) (Supplementary Figure S12). Much of the spontaneous DNA damage in cells is oxidative (98), an effect that is amplified when cells are grown in rich media with aeration (as is the case for most of the cell growth experiments in this study). We thus isolated plasmid pSTL78 from cells grown anaerobically. Over-expression of RecF again uniquely eliminated the duplex DNA circles from the plasmid prep (Supplementary Figure S12). All of these experiments were carried out at least 3 times with identical results. Together, these results suggest that RecF over-expression results in considerable damage to plasmid replicons with a potential increase in ssDNA. Unfortunately, the smear observed does not allow for differentiation between ss- and dsDNA formation.

In an attempt to bypass this limitation, the DNA present in the purified samples (pBR322) was further analyzed by electron microscopy using the cytochrome C method (81). This method allows for the differentiation between ss- and dsDNA regions of a DNA molecule that can be quantified (Figure 7B and Supplementary Figure S13). For all strains, in the absence of arabinose, the majority of the DNA observed was circular double stranded with a few molecules exhibiting small open regions of ssDNA (Figure 7B and Supplementary Figure S13). Little change was observed when arabinose was added to the control strain with a non-inducible wt promoter (native promoter). However, when RecF was over-expressed, the circular dsDNA essentially disappeared in the purified sample. The DNA molecules observed were largely either linear branched double stranded DNA with single-stranded regions or single-stranded DNA with small dsDNA patches. The DNA purified after RecF_K36R_ over-expression was prominently double stranded circles but with a slight increase in long linear molecules and a minority of single-stranded DNA with short dsDNA patches. The EM analysis is consistent with the idea that RecF over-expression results in an increase in plasmid damage that either precludes plasmid isolation by the standard preparation or increases ssDNA in the plasmids.

Reasoning that gap formation occurring on plasmid DNA can eventually lead to plasmid loss even with a relatively stable multi-copy plasmid like pBR322, we examined the effect of RecF over-expression on plasmid loss using a much different and independent assay (Supplementary Figures 12 and 14). A pBR322 vector in which *lacIZ* has been cloned (pEAW1232) was used for a blue/white color screen assay. To determine the number of cells losing the plasmid (indicated by colonies that are white), the plasmid was transformed into strains deleted of the lac operon so that all the lac genes are encoded by the plasmid (Supplementary Figure S12 C and Supplementary Figure S14). A modest but consistent increase in white colonies lacking plasmid was observed in cells exposed to RecF over-expression for 16 h compared to the control or the ATPase deficient RecF strain. About 10% of the cells entirely lost the multicopy pBR322 derivative plasmid, but only upon over-expression of RecF. A similar effect was observed when the assay was carried out with the pRC7 plasmid (also encoding the gene for β-galactosidase), which has a lower copy number and is much more easily lost (73) (Supplementary Figure S12D). The increase in plasmid loss confirmed that some kind of DNA damage that is deleterious to small replicons occurs upon RecF over-expression in an ATPase-dependent manner. Together, the visualization and the analysis of the plasmid DNA, along with the plasmid loss assay, implicates RecF over-expression with replisome instability and an increase in ssDNA gap formation.

### RecF interacts with the DnaN β-clamp and the DnaG primase

**Figure 8.**
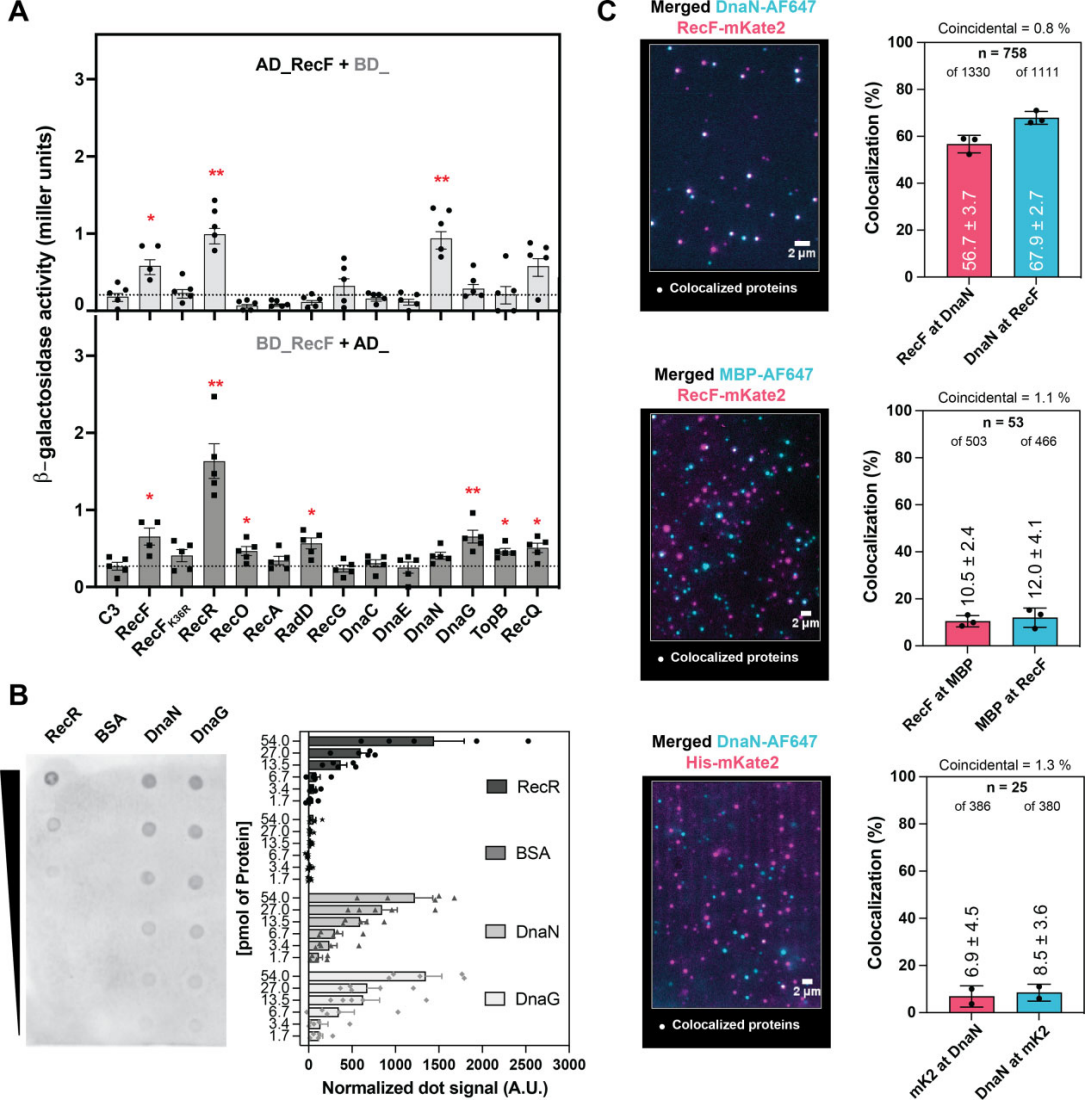
RecF interacts with β-clamp and DnaG. The interaction between RecF and partners was tested *in vivo* by yeast-two hybrid (**A**) and *in vitro* by far western blot (**B**) or by single-molecule interaction assay (**C**). (**A**) Combination of yeast transformed with RecF fused to either domain activator (AD) upper panel or binding domains (BD) lower panel and the control C3 or the indicated protein, involved either in DNA repair (RecF, RecF_K36R_, RecR, RecO, RecA, RadD, RecG, TopB, RecQ) or DNA replication (DnaC, DnaE, DnaN, DnaG), fused to the other domain were used. The strength of the interactions was tested by beta-galactosidase activity. A series of four or five biological replicates were carried out for each combination. (**B**) Far western blot using RecF as prey and RecR, BSA, DnaN and DnaG as baits. Increasing concentration from bottom to top of baits were spotted on the nitrocellulose membrane. Membrane was incubated with 0.2 μM of RecF before washing, incubation with primary and secondary antibodies to reveal the interaction partners. The interaction with partners was quantified as described in the method section. The average and s.e.m. of four biological replicates were plotted for each protein, dot represent individual result of each experiment. (**C**) Single-molecule interaction assay of fluorescently labelled proteins was used to analyze the colocalization of RecFmKate2 and DnaN_AF647 with partners. Merged channels of the dual color imaging of the protein and colocalization analysis histograms. The number of colocalized foci *n* and the total number of proteins are indicated for each condition. The coincidental colocalization (random) and the experimental percentage of colocalization of one protein to another are indicated in the histograms.

The data presented so far suggested a direct link between RecF and the replisome. We therefore sought more direct evidence for such an interaction. The interaction between RecF and putative replisome partners was investigated by the yeast-two hybrid assay *in vivo* (Figure 8). The interactions were probed by measuring β-galactosidase activity. First, a combination of yeast transformed with RecF fused to one domain (activator −AD or binding domains −BD) and the control C3 fused to the other domain were used as negative controls. A combination of RecF fused with either domain or RecF on one domain and RecR on the other were used as a positive control for RecF interaction. The β-galactosidase activities of the negative controls were respectively 0.21 (AD_RecF) and 0.27 units (BD_RecF) (Figure 8). The β-galactosidase activity increased to 0.655 for RecF/RecF interaction, and to 1.14 (AD_RecF) and 1.63 units (BD_RecF) for the RecF/RecR interaction. Yeast cells were further transformed with a combination of plasmids of RecF fused with one domain and another protein with the other, to test the interaction of RecF with proteins involved in DNA repair (RecA, RecF_K36R_, RecO, RadD, RecG, TopB, RecQ) and DNA replication (DnaC, DnaE, DnaN, DnaG). Among the different combinations tested, a significant increase was observed for AD_RecF and BD_DnaN harboring an activity of 1.05 units. A milder increase was also detected for RecF_BD and AD_DnaG showing an activity of 0.65 units. Much smaller signals were seen for RecF_BD with AD_ RecO, RadD, TopB and RecQ presenting activities of ∼0.5 units. Altogether, these yeast two hybrid assays revealed significant interactions between RecF and several potential partners, with the strongest interactions seen with the two clamp-like proteins, RecR and DnaN, followed by the interaction observed with the DNA primase DnaG.

The interactions between RecF and the two newly identified partners DnaN and DnaG were further corroborated by far-western blot (Figure 8 and Supplementary Figure S15), a method used previously to identify DnaN interaction partners (86). RecR and BSA were used as positive and negative controls respectively. RecR, BSA, DnaN, DnaG and SSB purified proteins were serial diluted and spotted 54 to 1.7 pmol on the membrane. After blocking, the RecF protein was added to a fresh blotting solution at the concentration of 0.2 μM, before further incubation with primary and secondary antibodies. This allowed detection of protein-protein interactions with RecF used as prey. As expected, after visualization no signal was detected for BSA and SSB (Supplementary Figure S15) while signal was detected for RecR. Signals of the same order of magnitude as RecR were detected for DnaN and DnaG.

The interaction of RecF and DnaN was further validated by single molecule microscopy. This method was previously used to demonstrate the absence of polymerase core exchange in solution when pre-assembled with the clamp loader (87). Purified RecF-mKate2 and DnaN-AF647 were mixed. As a control, RecF-mKate2 was mixed with MBP-AF647 and DnaN-AF647 was mixed with His-mKate2 (Figure 8) to make sure the results did not involve an anomalous interaction with the protein fusion components. Mixtures were incubated for 20 min before imaging. For the controls, colocalization was around 10%. In contrast, RecFmKate2 and DnaN_AF647 were found to be highly colocalized (∼60%). The analysis of the colocalization shell area is indicative indicative of a close interaction (Supplementary Figure S15). Together, these three protein–protein interaction assays confirm the interaction between RecF and DnaN and also indicate a potential interaction with DnaG.

The amino sequence analysis of RecF (Supplementary Figure S16) generated with Consurf (99–103) did not reveal a clear clamp binding motif ‘CBM’ or alternative motifs previously shown in partners to directly interact with DnaN (104–112). Nevertheless, AlphaFold (113,114) was successfully used to predict potential heteromultimers, including a heterodimer (Supplementary Figure S16). All of the predicted structures positioned RecF interacting with DnaN with at least one contact involving the region surrounding the loop consisting of residues 167–171 of RecF. However, they do not all map RecF interacting with the cleft of DnaN.

### *In vitro*, RecF triggers ssDNA gap formation during replication

**Figure 9.**
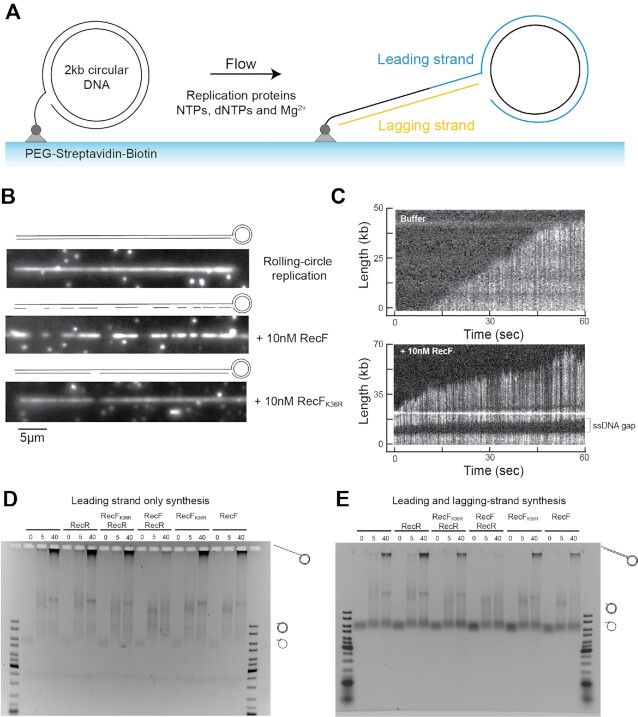
Gap formation during *in vitro* replication at physiological concentrations of RecF ATPase protein. The capacity of physiological concentrations of RecF ATPase protein to create DNA gaps during replication was tested *in vitro*. (**A**) Schematic representation of the experimental design. Circular 5′-biotinylated DNA is coupled to the functionalized surface of a microfluidic flow cell through a streptavidin linkage. Addition of *E. coli* replication proteins and nucleotides results in the initiation of DNA synthesis. Newly synthetized DNA products are extended by flow, labelled with DNA stain and visualized in real time using fluorescence microscopy. (**B**) Example of individual DNA molecules produced during pre-assembled rolling-circle replication in the absence of RecF, or in presence of 10 nM RecF or RecF_K36R_ proteins. The gray scale indicates the fluorescence intensity of stained DNA. (**C**) Kymographs showing the progression of DNA synthesis during rolling-circle replication without or with the addition of 10 nM RecF. A gap is identified by discontinuity of fluorescence intensity in a DNA molecule (**D**). Replication assay realized in batch on primed m13 circular DNA in the absence of the primase and the ribonucleotides, allowing only the replication of the leading strand. Samples of the ongoing replication were stopped at the indicated time. As mentioned, 20 nM of RecF or RecF_K36R_ or 40 nM RecR protein are added. (**E**). Replication assays realized in batch on primed M13 circular DNA including primase and the ribonucleotides, allowing the replication of both leading and lagging strands. Proteins are added at the same concentration as the batch replication of the leading strand only.

*E. coli* replication can be reconstituted and characterized *in vitro* using purified replisome proteins and coupled to a primed rolling-circle template, at both the ensemble and single-molecule level. The replication process can be monitored respectively either in bulk using an electrophoresis gel or in real time using single molecule fluorescence microscopy (92,115,116). To determine the effect of RecF and its ATPase activity on replisome stability and function, we set up replication assays in which purified RecF protein was added at both a physiologically relevant concentration (10 nM) (52,117) and also at a higher concentration to mimic RecF over-expression (100 nM). In some experiments, RecR was also added at a 2:1 ratio relative to RecF (Figure 9 and Supplementary Figure S17), with concentration as mentioned in the caption.

The single-molecule experiment is presented here first. The experimental design of this assay involves the replication of a rolling-circle DNA substrate tethered to the surface of a flow cell. The newly synthetized double-stranded DNA is stretched by a continuously applied laminar flow and visualized in real time using SYTOX Orange, a stain specific to double-stranded DNA (Figure 9A). Therefore, if single-stranded gaps are formed in the product strand, staining is discontinuous. In the experiments presented below, the replisome was pre-assembled onto the DNA template in solution. During replication a noticeable and concentration-dependent difference was observed in the frequency of gap formation when RecF was added to the reaction (Figure 9B and C). The basal frequency of visible gaps formed (in the absence of RecF) is on average 0.015 ± 0.002 gaps per μm DNA synthesized. This number increased modestly to an average of ∼0.027 gaps per μm DNA synthesized when either RecR or RecF_K36R_ proteins were added alone, respectively. However, this number was increased to more than 0.042 ± 0.003 gaps per μm DNA synthesized when 10 nM RecF was added. Gap formation increased to more than 0.059 ± 0.004 gaps per μm DNA synthesized when both RecF and RecR were added together (Supplementary Figure S17). These results suggest that RecR and RecF_K36R_ play a role in replisome impairment and uncoupling but the ATPase activity of RecF appears to be an important factor. Unlike the experiments using RecF over-expression *in vivo*, these experiments utilized mainly RecF concentrations consistent with normal *in vivo* RecF concentrations estimated to be in the range of 5–20 nM, equivalent to 18–68 RecF molecules per cell (52,117). When RecF was added alone at the higher concentration to mimic over-expression, the frequency substantially increased to 0.191 ± 0.008 gaps per μm DNA synthesized. This observation correlates well with the increase in replisome loss and uncoupling observed *in vivo* upon RecF over-expression. The analysis of the gap size also revealed that when RecR or RecF_K36R_ were added alone, the average size of the few gaps formed increased. Interestingly, the addition of wild type RecF has the opposite effect; gaps formed are smaller and a significant further decrease is observed for the combination of RecF and RecR. These observations suggest that the RecF ATPase activity might not only be involved in the gap frequency but also in the initiation of the downstream Okazaki fragment synthesis.

The gaps observed above must be occurring primarily on the lagging strand as the formation of leading-strand gaps would lead to termination of the rolling-circle replication reaction. To determine if the RecF ATPase effect on gap formation was lagging-strand specific, we set up ensemble primer-extension assays, on primed M13 DNA, allowing the replication of the leading strand alone or the leading and the lagging when the DnaG primase and the rNTPs were added (Figure 9DE). In both cases, the addition of RecR or RecF_K36R_ had no effect on replication; the intensity of the final product was similar to the intensity observed for the control (storage buffer). In contrast, a reproducible decrease in the final replication product was observed when 20 nM RecF was added. This effect was greater when RecF and RecR were added together. As the effect of RecF was seen in both replication assays, these results suggest an involvement of RecF ATPase in gap formation during the ongoing replication in both strands of the DNA, with perhaps a stronger effect on the lagging strand.

## DISCUSSION

The mechanism by which the RecFOR system is targeted to lesion-containing post-replication gaps is not understood. The most prominent targeting mechanism proposed to date involves specific RecF binding to the ends of gaps. However, as detailed in the accompanying paper (40), neither RecF nor the RecFR complex have the needed specificity for binding to gap ends. If binding to the most notable structural feature of a ssDNA gap—the gap ends—cannot explain targeting, then a protein-protein interaction becomes the most likely alternative. Here, we demonstrate an interaction between RecF and replisome components that opens an experimental path to solving the targeting conundrum and more. In principle, by interacting directly with the replisome that first senses the template lesion, replisome disengagement to create a post-replication gap could leave RecF behind, properly positioned to direct repair of that particular gap. The overall scheme has some elements that mirror the quite detailed speculation put forward by Kuzminov 25 years ago (60).

The case we present for RecF interaction with replisomes and RecF effects on those replisomes has multiple and varied components. In brief, (1) the toxicity associated with RecF over-expression is here associated with replisome destabilization; (2) there is a direct interaction of RecF with DnaN and possibly DnaG; and (3) RecF triggers gap formation *in vitro* at concentrations found *in vivo*. All of this work complements a growing literature associating RecF with the replisome (44,52,57–60,62). We expand on these three conclusions below.

Over-expression of the RecF protein is highly toxic to a bacterial cell (54,56). To date, the molecular basis of that toxicity has not been understood. We conclude that over-expression of wtRecF, with its ATPase intact, directly and negatively impacts replisome stability, limiting the capacity of cells to resume normal growth following RecF over-expression. RecF over-expression leads to dramatic cellular replisome loss, increased SSB particle number and size suggesting an increase in gap formation, increased recombination associated with post-replication gaps, and significant loss of small replicons (plasmids) even when they exist as multiple copies in the cell. The increase in gap-associated recombination, a large induction of the SOS response that is *recO-* and *recB-*dependent, and the loss of circular duplex plasmid circles all associate the RecF over-expression with replisome dissociation and an accompanying increase in ssDNA that would be expected if gaps were being formed. These observations support our proposal that the destabilizing effects on replisomes underlie the toxicity associated with RecF over-expression. A direct interaction between RecF and the replisome provides a better explanation for the observed replisome destabilizing effects of RecF over-expression than replisome collisions with randomly bound RecFR complexes. RecF_K36R_, which binds to dsDNA as well or better than RecFR, has no toxic effects when over-expressed at the same or higher levels. Unlike RecF, over-expression of RecO has no toxic effects. We thus propose that the effects of RecF over-expression reflect a RecF-replisome interaction and resulting replisome destabilization that explains the toxicity associated with RecF over-expression. We of course acknowledge that over-expression of any protein can create anomalies and that the results must be considered in that context.

The interaction between RecF and the replisome is readily demonstrated. A previous screen using a global pull-down assay to study new protein-protein interactions identified a potential interaction between RecF and DnaN (118). Here, we document a direct interaction between RecF and DnaN with three distinct methods. Yeast two hybrid experiments and far western blots documented the interaction between RecF and DnaN, along with a possible but much weaker interaction with DnaG. A strong co-localization of RecF and DnaN can also be observed by microscopic examination of single molecules, providing a third result confirming the interaction. These observations strongly suggest a direct interaction between RecF and the replisome that can explain the co-localization observed *in vivo* under normal growth conditions (52).

Finally, we demonstrate that addition of RecF protein to active replisomes in a single molecule experiment *in vitro*, at concentrations similar to those found *in vivo*, leads to replisome destabilization and significant lagging strand gap formation. Effects of RecF on leading strand replisomes can be demonstrated in bulk experiments. Although much remains to be done, these results, along with the replisome instability seen *in vivo* when RecF is over-expressed, suggest that RecF could be involved not only in targeting repair to post-replication gaps but also in the replisome disengagement that results in gap creation.

When combined with the extensive literature linking RecF in some manner to RecA filament formation in gaps, the results suggest an important link between gap formation at the replication fork via lesion-skipping and the subsequent processing of those gaps by RecA protein. That link appears to be organic to RecF function, as RecF co-localization with replisomes is frequent *in vivo* when RecF is present at physiological concentrations (52).

How do we tie all of this to the well-established function of RecF in loading RecA protein into gaps? RecF can clearly influence RecA filament assembly if it is properly positioned (46,47,50). However, neither RecF nor RecFR binds at gap ends with anything like the specificity required to direct these proteins to gaps *in vivo*. As already noted, specific RecFR binding to gap ends could be detrimental, as gaps are a feature of the replication process (119) and those arising from lagging strand DNA synthesis and mismatch repair are presumably much more abundant than lesion-containing post-replication gaps. So how could RecFR get to the location where it is needed to affect RecA filament formation at lesion-containing gaps without interfering with other aspects of replication? We suggest that RecF interacts in some manner with replisomes via DnaN (and possibly DnaG) and is placed at gap ends by the replisome in the process of gap generation via lesion-skipping. In this scheme, the replisome is the lesion sensor and the RecF-replisome interaction provides an avenue for placing RecF at the end of the lesion-containing post-replication gap.

**Figure 10.**
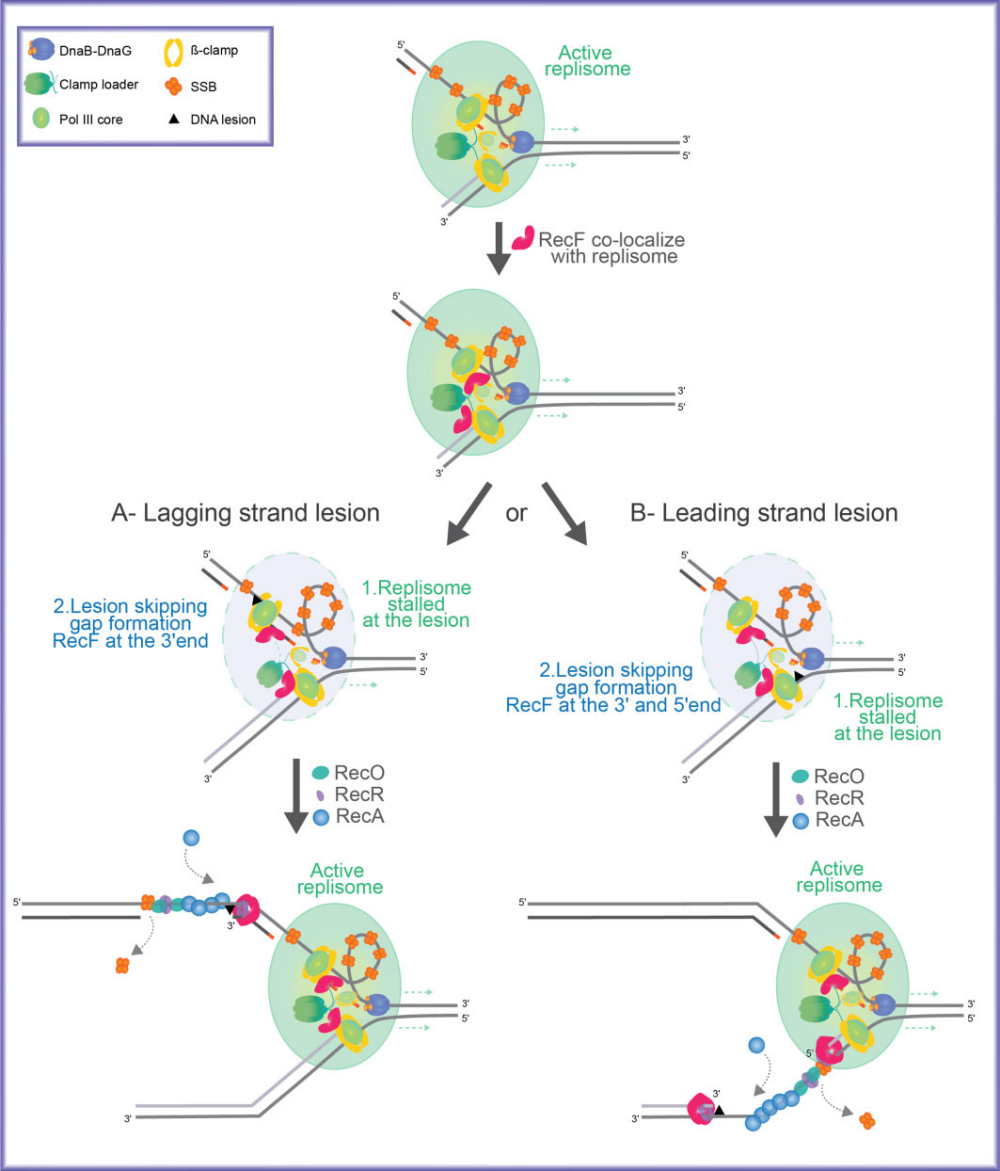
Model of RecF ATPase function near the replisome. Schematic representation of RecF ATPase activity triggering the localization of RecF near the replisome. The light green circle represents the stable replisome. In the front of the replication fork the DnaB helicase (dark blue) unwinds the dsDNA and interacts with DnaG (dark orange). DnaG promotes RNA priming on the lagging strand. On the lagging strand, the ssDNA region intermittently formed during replication is coated by the SSB protein (light orange tetramer). The clamp loader (dark green) interacts with the two polymerase cores of the leading and lagging strand, the clamp loader also interacts with a third PolIII core that would be loaded on the next RNA primed site. Those interactions allow the integrity of the replisome. The β-clamp (yellow) increases the processivity of PolIII is represented in yellow. Gap formation occurs upon encounter with a lesion. RecF (pink), initially associated with the replisome, is deposited at the 3′ end of the interrupted DNA strand. Stability of the bound RecF is increased by binding to RecR (purple). Gaps formed on the lagging strand have RecFR at one end of the gap. Gaps formed in the leading strand may have RecFR positioned at one or both ends of the gap. Finally, RecA is loaded onto the SSB coated DNA by the RecO (marine green) and RecR proteins at a site within the gap, potentially facilitated by RecR handoff from RecFR. The authors encourage readers to compare the elements of this model with speculation offered by Kuzminov in 1999 (60).

These ideas are outlined in the model in Figure 10. Although much of the scheme is speculative, it builds on the growing evidence that RecF interacts with the replisome and represents a first attempt to address the questions posed in the Introduction. This is also an attempt to accommodate many observations accumulated over decades as well as the current work. In particular, the model incorporates the proposed targeting role of the RecFR complex and the loading function of RecOR (21,45,120,121). The model also incorporates a handoff between RecF and RecR that is implied but not discussed in some earlier studies (50).

In the model of Figure 10, RecF first interacts with an active replisome through DnaN. When that fork encounters a lesion that triggers lesion-skipping (possibly with a disengagement mechanism that utilizes RecF), the replisome-associated RecF is left behind at the gap near the 3′ terminus where replication was interrupted. If the replisome undergoes some conformational change that leads to disengagement and lesion-skipping, one that does not occur during normal lagging strand DNA synthesis, this could provide a molecular signal for specific RecF positioning at the end of what then becomes a lesion-containing post-replication gap. Gaps generated during lagging strand DNA synthesis or mismatch repair would not be affected.

RecF dimerization and interaction with RecR strengthens the binding. The gap is likely expanded by the action of RecJ (122). RecA filaments, once nucleated, grow primarily in the 5′ to 3′ direction. If the RecFR is near the 3′ gap terminus, this would position RecFR away from RecA nucleation sites within the gap, at the end where it could limit RecA filament extension beyond the gap (44,120). However, via looping of the intervening DNA, RecF could presumably transfer RecR to a RecO monomer interacting with SSB within the gap. A handoff of this kind could be part of a mechanism to constrain RecOR function to gaps where repair was needed. An interaction between RecF and DnaG might also facilitate a positioning of RecF at the 5′ gap terminus as DNA synthesis is re-initiated following gap creation.

The best way to control RecO action is to control its access to RecR and SSB, both of which are essential to its RecA-loading function. The cellular concentrations of the RecF, RecO and RecR proteins are normally quite low. In addition, there are many cellular proteins that bind to the SSB C-terminus (123,124). Unlike the situation *in vitro* where purified proteins are used, the many SSB-binding proteins *in vivo* could limit RecO access to ssDNA gaps. A handoff scheme where RecFR marked the gaps requiring repair intervention and then recruited RecO could be part of a broader regulatory process controlling RecA filament formation and its capacity to block replication forks and induce SOS.

The interactions between RecF and the replisome may not result in significant replisome instability under normal cellular conditions where RecF is present at low levels. However, over-expression of RecF might lead to replisome impediment. If RecF is directly involved in gap creation, it might facilitate the intrinsic capacity of the replisome for lesion-skipping (7), using its ATPase function. RecF over-expression could thus trigger more frequent replisome disengagement with the template. Alternatively, the increased replisome instability noted with RecF-over-expression could be a deleterious byproduct of RecF-replisome interactions that are normally inconsequential.

In the scheme of Figure 10, there are multiple possible functions for the still-enigmatic RecF ATPase. The RecF ATPase may somehow facilitate replisome disengagement and/or replication restart during gap formation. Dissociation of RecF from the replisome and placement at a gap end could require a conformation change involving ATP. Another possible role could involve a RecR hand-off to RecO. The failure to see RecO and RecF co-localization *in vivo* could simply reflect the very transient nature of these hand-offs. The present work provides a starting point for a broader exploration of RecF function at the replisome.

There is a growing literature indicating that lesion-skipping and the repair of the resulting post-replication gaps occurs multiple times each replication cycle under normal and unstressed growth conditions (8,9,122,125). Most Holliday junction formation under these same conditions arises due to repair of post-replication gaps (126). The replisome destabilization effects of RecF over-expression suggest a functional link to the replication fork that correlates well with the frequent co-localization of RecF with replisomes under normal conditions where RecF is not over-expressed ((52); this work). These observations are readily explained by the RecF-DnaN interaction documented in this work. The RecF-DnaN interaction may even help rationalize the evolutionary positioning of the *recF* gene in the *E. coli* genome, immediately adjacent to *dnaN*, in an operon otherwise dedicated to replication. A possible role for RecF in the formation of post-replication gaps and/or a replisome-mediated deposition of RecF near post-replication gaps, along with its implications for regulating the activity of the RecFOR system, provides both a new way to think about the RecFOR epistasis group and a path for future investigation. Combined with results in the accompanying paper, we can now refocus efforts to explain specific RecFOR targeting to lesion-containing post-replication gaps. The targeting mechanism is likely embedded in a RecF interaction with key replisome proteins rather than a specific interaction of RecF with DNA gap ends.

## DATA AVAILABILITY

The data underlying this article are available in the article and in its online supplementary material.

## Supplementary Material

gkad310_Supplemental_File

## Notes

Present address: Sarah S. Henrikus, Macromolecular Machines Laboratory, The Francis Crick Institute, London, UK.
