# Supplementary material for: RecF protein targeting to post-replication (daughter strand) gaps II: RecF interaction with replisomes
Source: Nucleic Acids Res. 2023 May 1;51(11):5714–42. doi: 10.1093/nar/gkad310 (PMC10287930; doi:10.1093/nar/gkad310)
Supplement: gkad310_Supplemental_File [file gkad310_supplemental_file.docx]

**Supplementary figures and captions.**

**
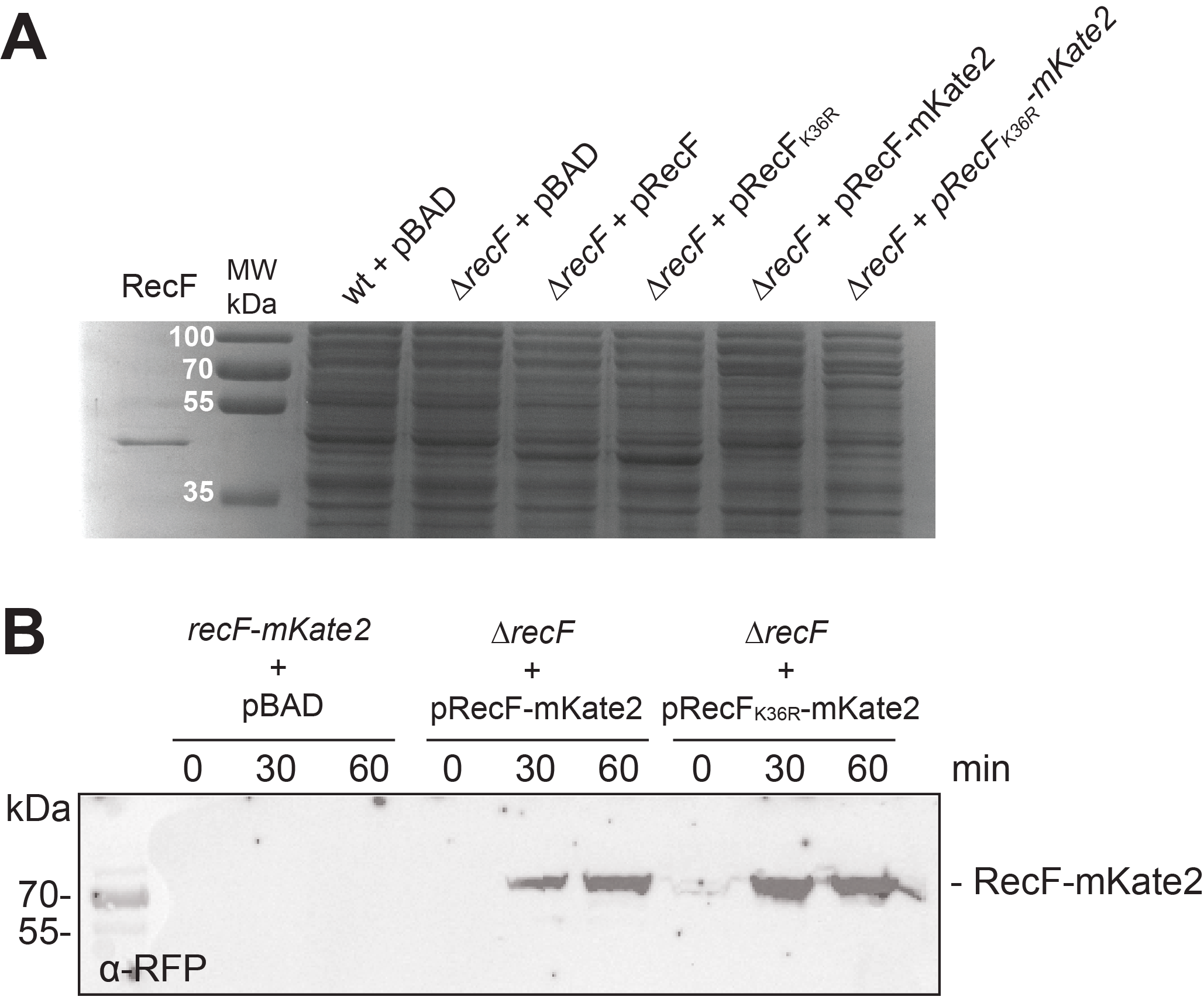
**

**Supplementary Figure 1: RecF expression level from plasmid.**

**A and B** Cells carrying pBAD derivatives vectors were grown in EZ-glycerol amp at 37ºC to mid log phase (OD_600_ = 0.2-0.5). **A** RecF expression levels 1 h hour after arabinose addition was evaluated by SDS-PAGE Coomassie stained. **B** The expression level of the mKate2 versions of RecF was assayed at time 0, 30 and 60 min of the over-expression by western-blot anti-RFP (mKate2).

**
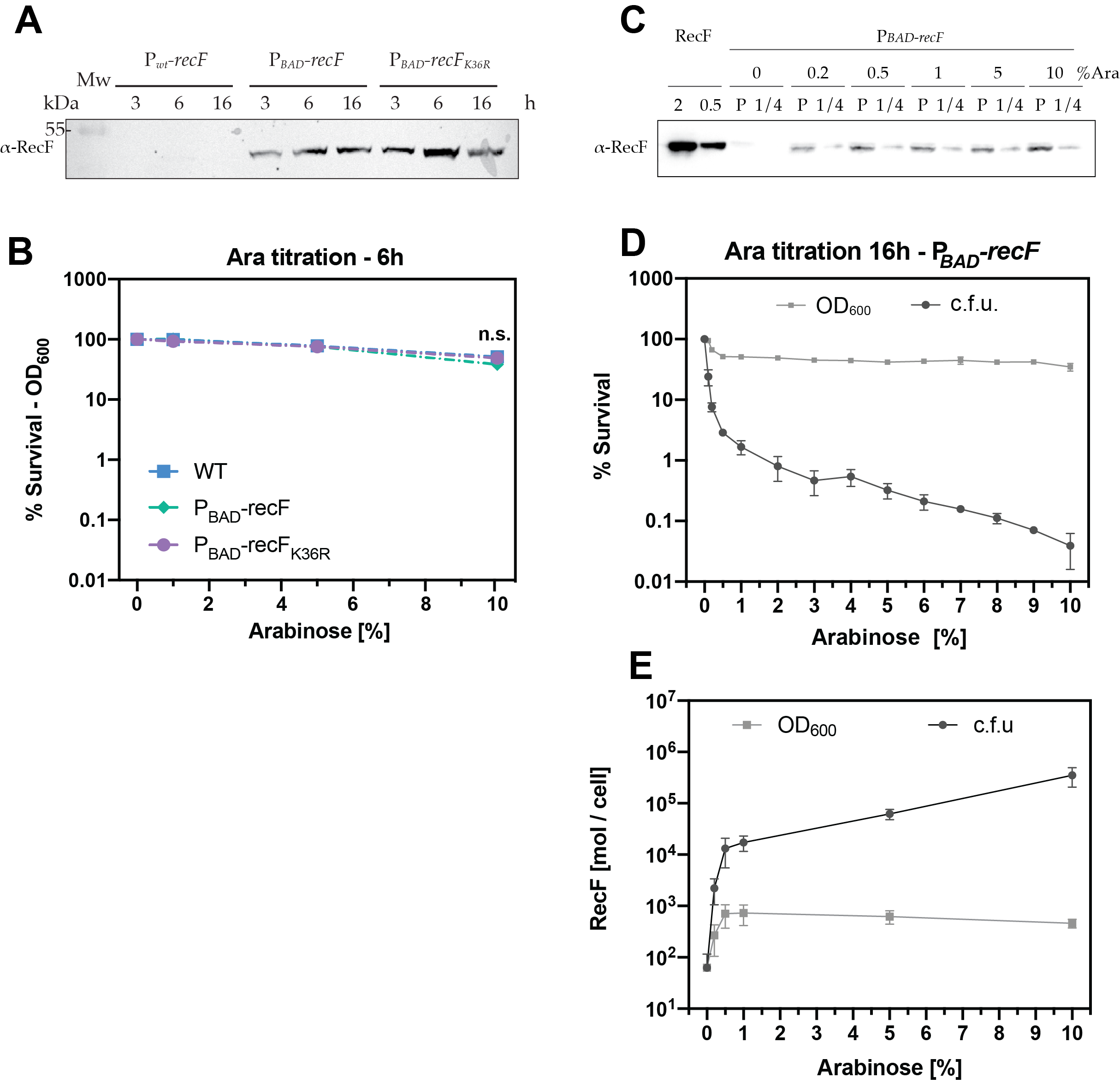
**

**Supplementary Figure 2: Chromosomal construct validation and arabinose titration.**

RecF expression from the chromosomal construct upon increased concentration of arabinose, its expression level and its toxicity were tested. **A** RecF expression in the parental (WT) and chromosomal over-expression strains (EAW1130 and EAW1148) was determined by Western-blot with anti-RecF at 3, 6 and 16 h after arabinose addition. **B** The mean optical density of strains incubated for 6h with the increased concentration of arabinose previously used for the RecF and GyrB immunoblot is reported. **C, D and E** The RecF concentration in EAW1130 expressed in molecules per cell was estimated by immunoblot 16h after addition of the indicated concentration of arabinose. **C** Immunoblot anti-RecF**,** pure diluted culture**s** were loaded alongside known concentrations of purified RecF to evaluate RecF concentation. **D** Cell survival was evaluated by both optical density and c.f.u. obtained by spreading 100 µL of the adequate dilution on LB plates incubated 16h at 37˚C. **E** The concentration of the number of RecF molecules per cell was estimated using either c.f.u. or OD_600_. For each experiment, a biological triplicate was carried out, a representative membrane was selected for the immunoblot.


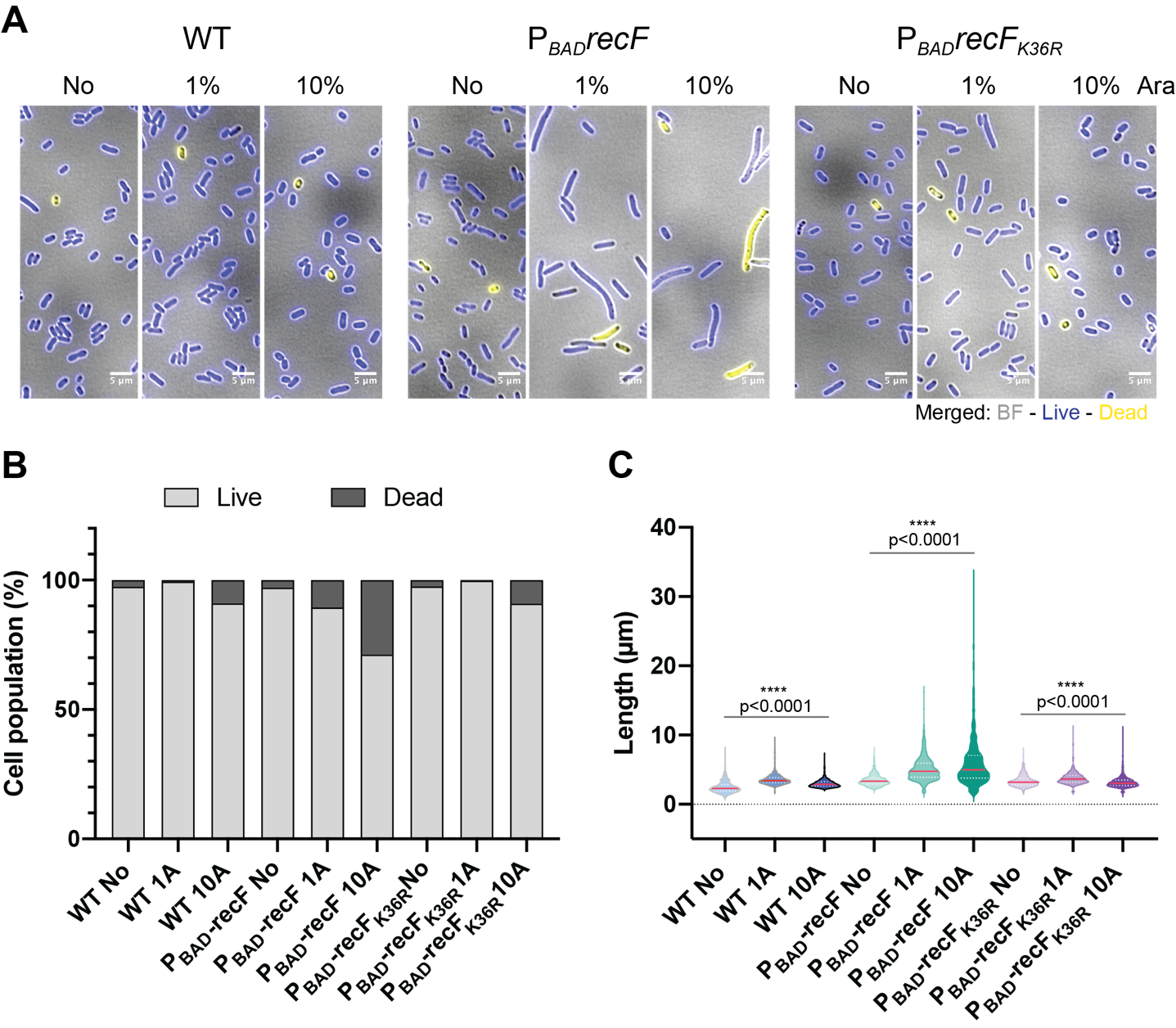


**Supplementary Figure 3: Imaging of the chromosomal construct live and dead single cell.**

Live and dead cell probe was used to determine the survival of cells and morphology after 16h of incubation with 0, 1 or 10% arabinose for the wt and the chromosomal constructs (EAW1130 and EAW1148). For each condition, more than 400 cells were analyzed. **A** Merged images of the brightfield, alive (GFP channel with blue LUT) and dead (DsRed channel with yellow LUT). **B** Stacked histogram representing the proportion of live and dead cells. **C** Violin plot of the cell length.


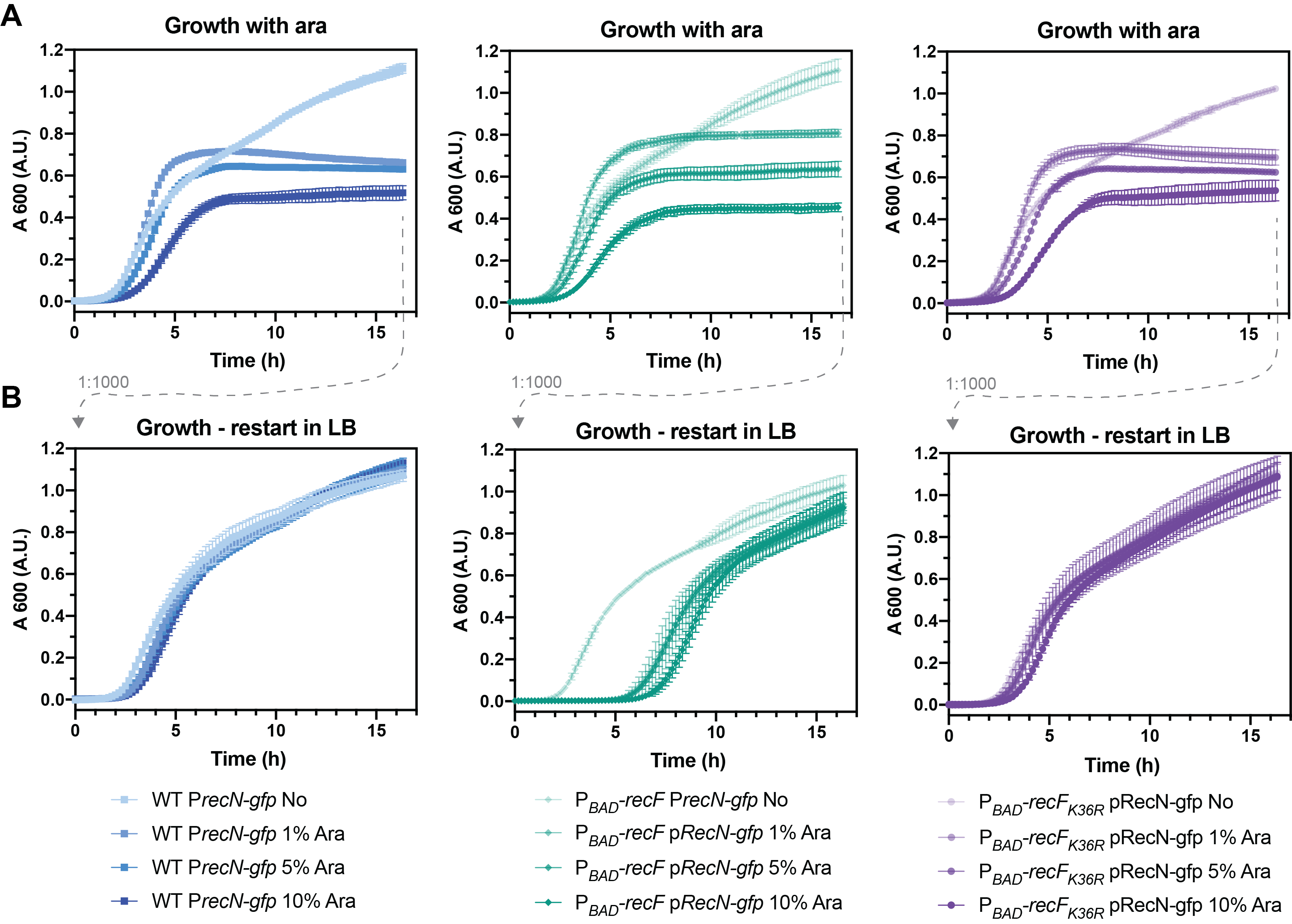


**Supplementary Figure 4: Growth restart of the chromosomal construct following arabinose addition.**

The ability of chromosomal constructs cell to resume growth following 16 h of arabinose exposure was determined by optical density. Transformed strains wt EAW1130 or EAW1148 carrying the *P-recN-gfp* plasmid were used in LB amp culture, only the absorbance at 600 nM is reported. **A** The cells were first cultivated in LB amp supplemented with the indicated concentration of arabinose (0, 1, 5 or 10%) for 16h. **B** Fresh cultures carried out in LB amp only were inoculated with cells of the previous culture (A.) and growth was recorded for 16 more hours.


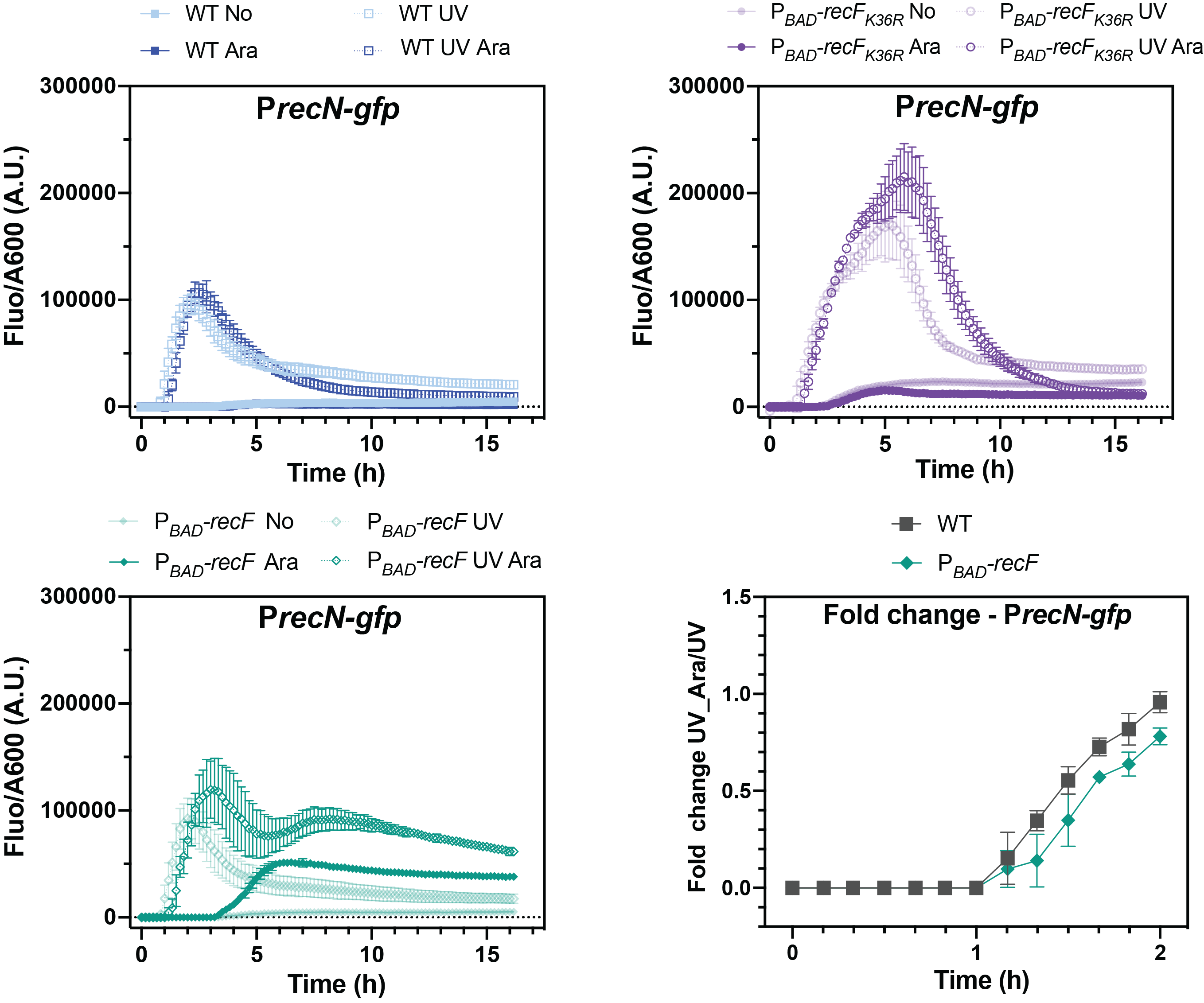


**Supplementary Figure 5: SOS induction upon RecF over-expression combined or not with UV exposure.**

The effect on SOS induction of RecF over-expression and UV exposure alone or combined was assayed using chromosomal constructs (wt, EAW1130 and EAW1148) transformed with the *P-recN-gfp* plasmid. In this case 3 mL of LB amp were first inoculated with a saturated culture 1:100 and gown until OD600 ~0.2. 1 mL of cell wash with 1 mL 1x PBS. 400 µL of washed cells were transferred to a 24 microwells plate and exposed to 100 J/m^2^. Unexposed and exposed cells were then diluted 10 times in fresh LB amp supplemented or not with 10% arabinose. Cells were grown at 37ºC, the absorbance at 600 nM and the fluorescent signal of the gfp were recorded every 10 min for 16h. The fluorescence divided by the absorbance is reported for each strain. The fold change observed for cells which experienced arabinose addition combined with UV relative to cell that only experienced UV is reported for the first 2h for the wt and EAW1130 strains.

**
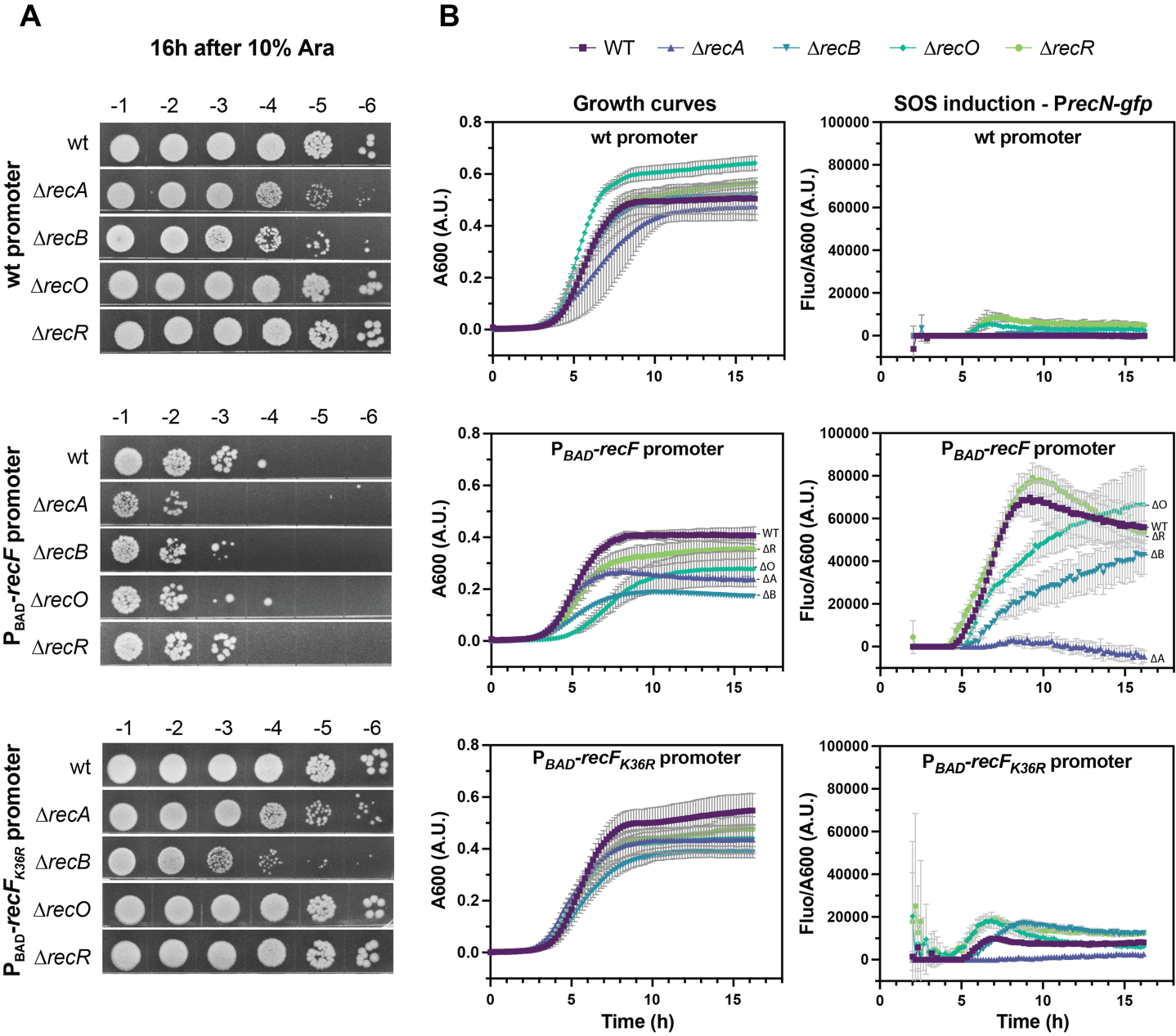
**

**Supplementary Figure 6: Toxicity, growth and SOS induction of chromosomal construct combined with homologous recombination deletion mutant.**

The effect of arabinose addition on the chromosomal constructs (wt, EAW1130 and EAW1148) combined with single deletion mutants involved in the homologous recombination (∆*recA*, ∆*recB*, ∆*recO* or ∆*recR*) was tested on **A** toxicity, and on **B** growth and SOS induction for strains transformed with the *P-recN-gfp* plasmid. Arabinose was introduced at 0 time at a level of 10%. Data obtained prior to 1.5 hours of growth exhibited high degrees of error due to very low cell densities and was not included.


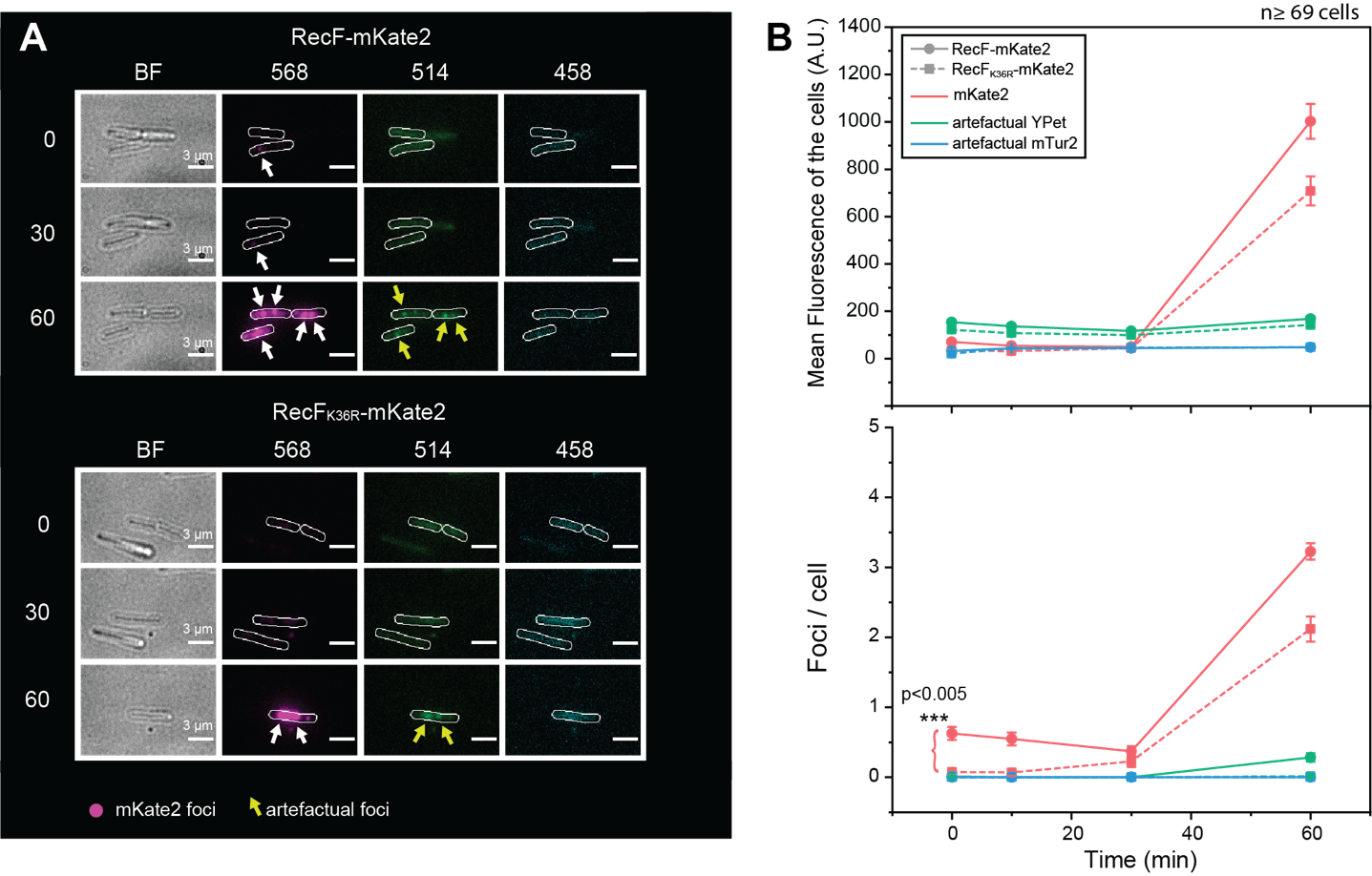


**Supplementary Figure 7: Control of mKate2 interference signal under over-expression.**

Fluorescence channel interference assay during RecF-mKate2 or RecF_K36R_-mKate2 over-expression, using strains harboring the mKate2 constructions as sole tagged protein. **A** Single cell imaging of EAW629 (∆*recF*) transformed by pEAW1128 (RecF-mKate2) or pEAW1202 (RecF_K36R_-mKate2). Cells were loaded in a flow chamber incubated at 37ºC and a continuous flow of EZ-glycerol amp media supplemented with 0.2% arabinose was applied to allow cells to grow in the presence of inducer. Captures were realized in the brightfield, 568 nm (mKate2), 514 nm (YPet) and 458 nm (mTur2) channels at the indicated time. White arrows indicate mKate2 foci, yellow arrows indicate the artifactual foci created by the mKate2 signal through the 514 nm channel. **B** Representation of the mean fluorescence per cell (upper panel) and the number of foci detected (mKate2 or artifactual) in each channel.

**
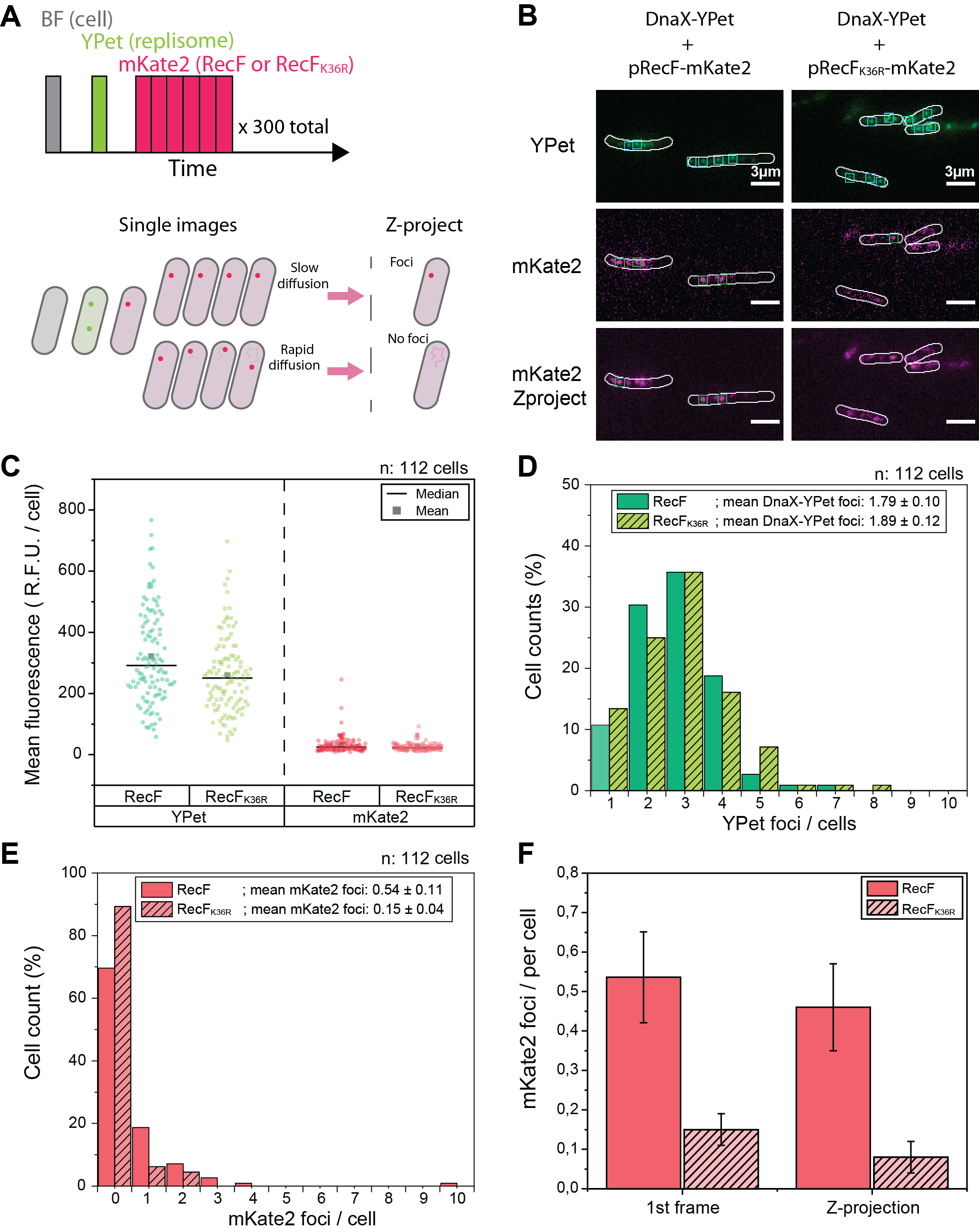
**

**Supplementary Figure 8: RecF ATPase activity is involved in foci formation.**

The requirement of RecF ATPase activity on the focus behavior was determined before over-expression, by fluorescence microscopy. Cells were loaded on a home-built flow chamber for imaging. **A** Description of the rapid acquisition experimental design. Two-color imaging of cells carrying a replisome marker along with RecF-mKate2 or RecF_K36R_-mKate2. A single capture of the brightfield, followed by a single capture in the YPet channel allowed to determine replisome (DnaX-YPet) foci position in the cells. Then a succession of 300 captures in the mKate2 channel allowed the study the behavior of the mKate2 (RecF) foci. The average Z-projection of the 10 first images of the 568 nm channel was used to determine the behavior of the mKate2 foci for the first 500 ms. **B** Captures of single cells with replisomes labelled (DnaX-YPet) and RecF-mKate2 or RecF_K36R_-mKate2. **C** Dot plot representing the mean fluorescence signal of the YPet or mKate2 fluorophore for each cell. **D** Histogram representing the distribution of the DnaYPet foci number per cell relative to the total cell population. The values represent the mean ± SEM for n: 197 cells. **E.** Histogram representing the distribution of the number of mKate2 foci per cell relative to the total cell population observed in the 1^st^ frame. **F** Histogram representation of the average number of mKate2 foci per cell detected on the initial frame still detected on the Z-projection of the first 10 frames.


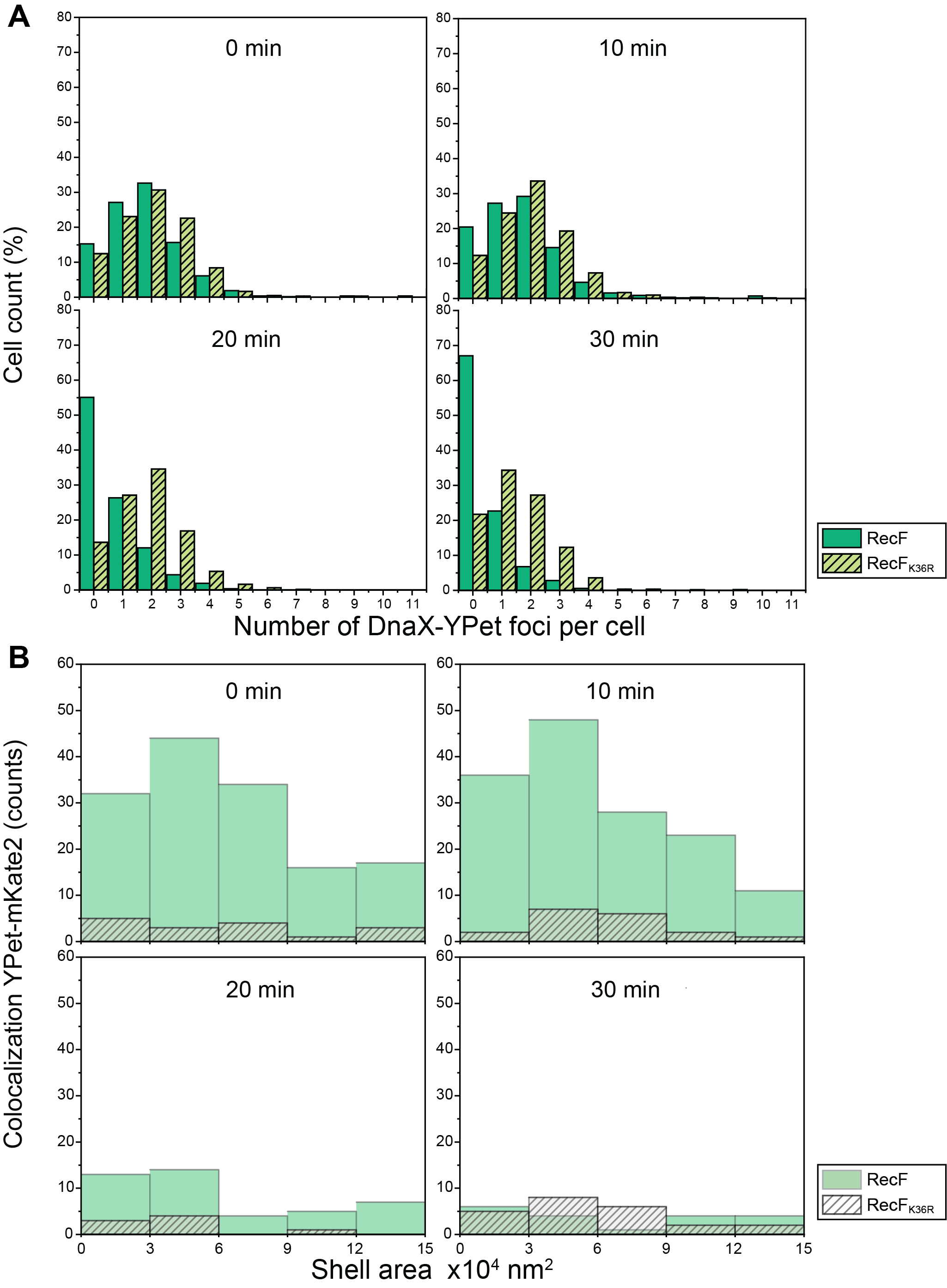


**Supplementary Figure 9: Distribution of the replisome foci per cells in the two-color strains.**

**A.** Distribution of the number replisomes (DnaX-YPet) per cell relative to the total cell count. Histogram representation of the cell distribution at time 0, 10, 20 and 30 min after arabinose addition. Distribution of replisome foci of cells expressing RecF-mKate2 or RecF_K36R_-mKate2 are respectively represented in dark green and light green dashed bars. **B.** Distribution of the total colocalization counts between the YPet-mKate2 in function of the colocalization shell area, cells expressing RecF-mKate2 or RecF_K36R_-mKate2 are respectively represented in green and or white dashed bars. A shell area value close to zero was obtained for foci in close proximity whereas higher values was obtained for more distant colocalized foci.


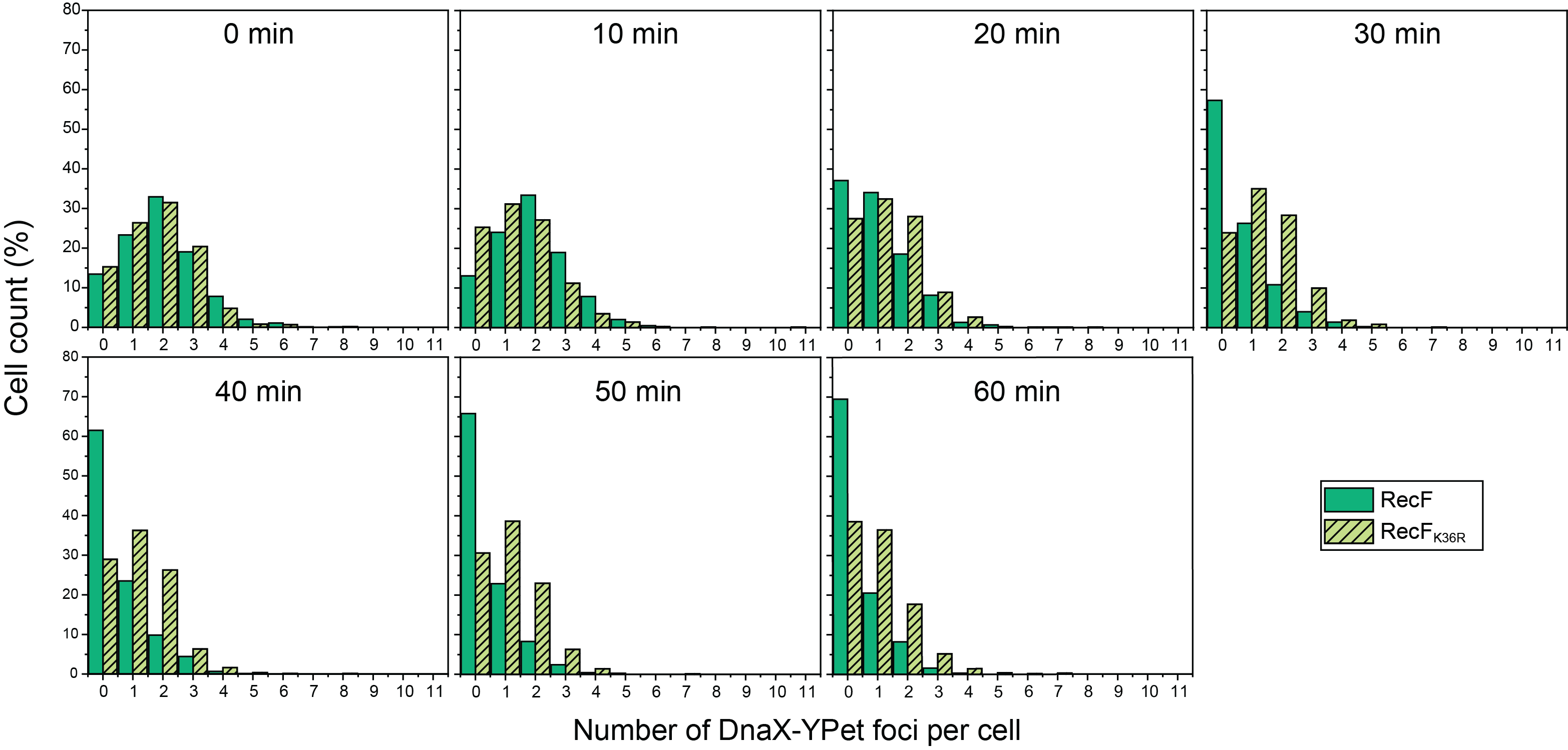


**Supplementary Figure 10: Distribution of the number of replisome foci per cells in the single-color strains.**

Distribution of the number of replisomes (DnaX-YPet) per cell expressed as percentage of the total cell count. Histogram representation of the cell distribution at time 0, 10, 20, 30, 40, 50 and 60 min after arabinose addition. Distribution of the replisome numbers per cell. RecF or RecF_K36R_ were respectively represented by dark green and light green dashed bars.


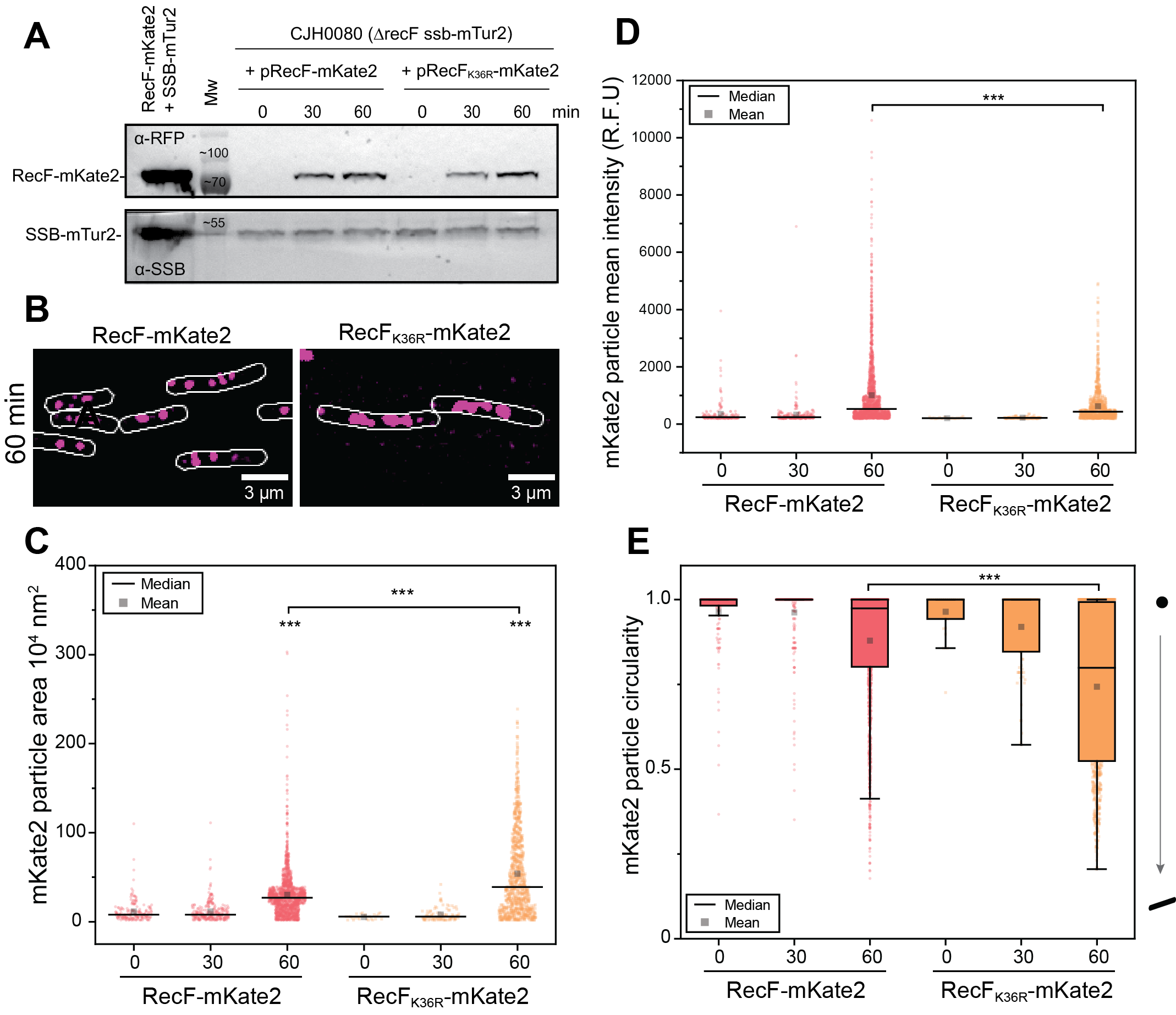


**Supplementary Figure 11: Immunoblots and characteristics of RecF-mKate2 and RecF_K36R_-mKate2 particles.**

**A** The expression level of RecFmKate2 and SSB-mTur2 at t0, 30 and 60 min following RecF-mKate2 variants overexpression was evaluated by immunoblots anti-RFP and anti-SSB respectively. **B** to **E** The particles formed by RecF-mKate2 or RecF_K36R_-mKate2 during over-expression were analyzed from the two-color imaging with SSB-mTur2 as indicated in the Method section. **B** Images of RecF-mKate2 or RecF_K36R_-mKate2 in single cells 60 min after arabinose addition. A discoidal filter (1-4) has been applied with Fiji. **C** Dot plot representation of the area of the mKate2 particles at time 0, 30 and 60 min for RecF-mKate2 (red) and RecF_K36R_-mKate2 (orange). **D** Dot plot representation of the mean particle fluorescence signal at time 0, 30 and 60 min for RecF-mKate2 (red) and RecF_K36R_-mKate2 (orange). **E** Particle circularity analysis observed with Fiji and plotted as dot plot on which a box of 95% confidence interval is represented at time 0, 30 and 60 min for RecF-mKate2 (red) and RecF_K36R_-mKate2 (orange). **C, D** and **E** For RecF-mKate2 the number of particles were respectively n:187, 278 and 1841 for 0, 30 and 60 min, and n:25, 99 and 993 for RecF_K36R_-mKate2. The median value is represented with a bar and the mean by a grey square. Significant differences were analyzed by t-test, significant difference relative to time 0 of the same strain and is represented under the parenthesis and significant difference between strain for the same time is represented on the top of the parenthesis (***: p≦ 0.005).


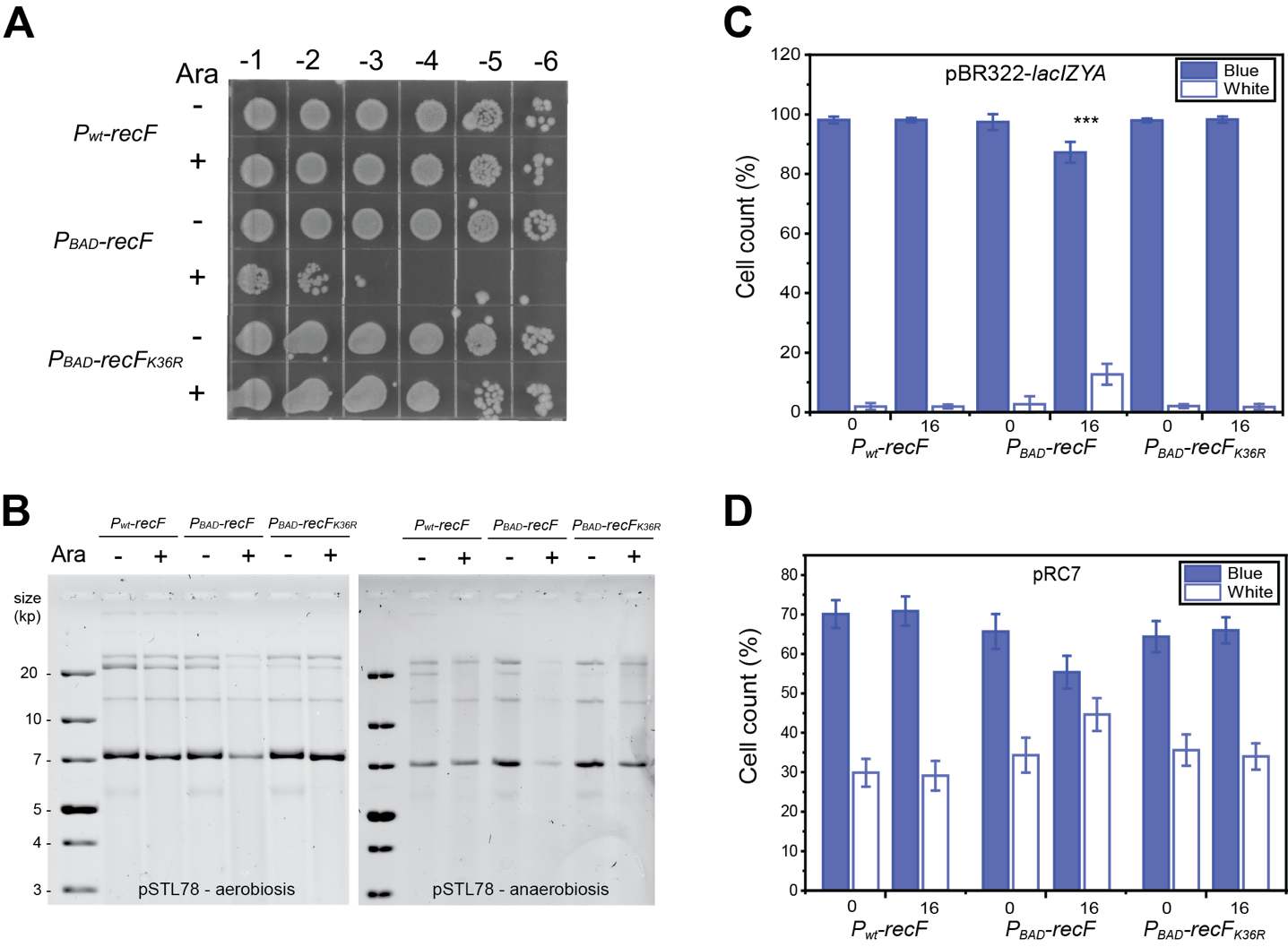


**Supplementary Figure 12: Toxicity, DNA damage and plasmid loss caused by RecF over-expression on pBR322 derivatives and pRC7 replicons.**

Cells were grown for 16 h in LB amp with or without addition of 10% arabinose. **A** Cells carrying the pBR322 plasmid were serially diluted and spotted on LB amp plates to assay survival after over-expression. **B** Electrophoresis of 250 ng of pSTL78 plasmid DNA purified from strains grown in the presence (aerobiosis) or absence of oxygen (anaerobiosis). **C** The effect of the replisome dissociation on a small replicon was determined *in vivo* using a pBR322 plasmid loss assay. Strains deleted of *lacIZYA* were transformed by the pEAW1232 (pBR322 carrying the *lacIZYA*). The ability of cells to maintain the plasmid was determined at time 0 and after 16 h of culture without Amp or Tet in presence of 10% arabinose. Adequate dilutions of the cultures were spread on Xgal-IPTG plates. Cells able to retain the plasmid formed blue colonies. The histogram represents the percentage of white and blue colonies for a biological triplicate at the indicated time. The significance relative to the wt strain in the same condition was determined by t-test (***: p≦ 0.005). **D** pRC7 plasmid loss assay under RecF over-expression. The Δ*lacIZYA* strains encoding the wild type chromosomal *recF* gene or the chromosomal over-expression constructs were transformed with the pRC7 vector. The ability of each strain to maintain the plasmid was determined by blue/white screen at time 0 and 16 h after the concomitant addition of arabinose and the omission of the amp.


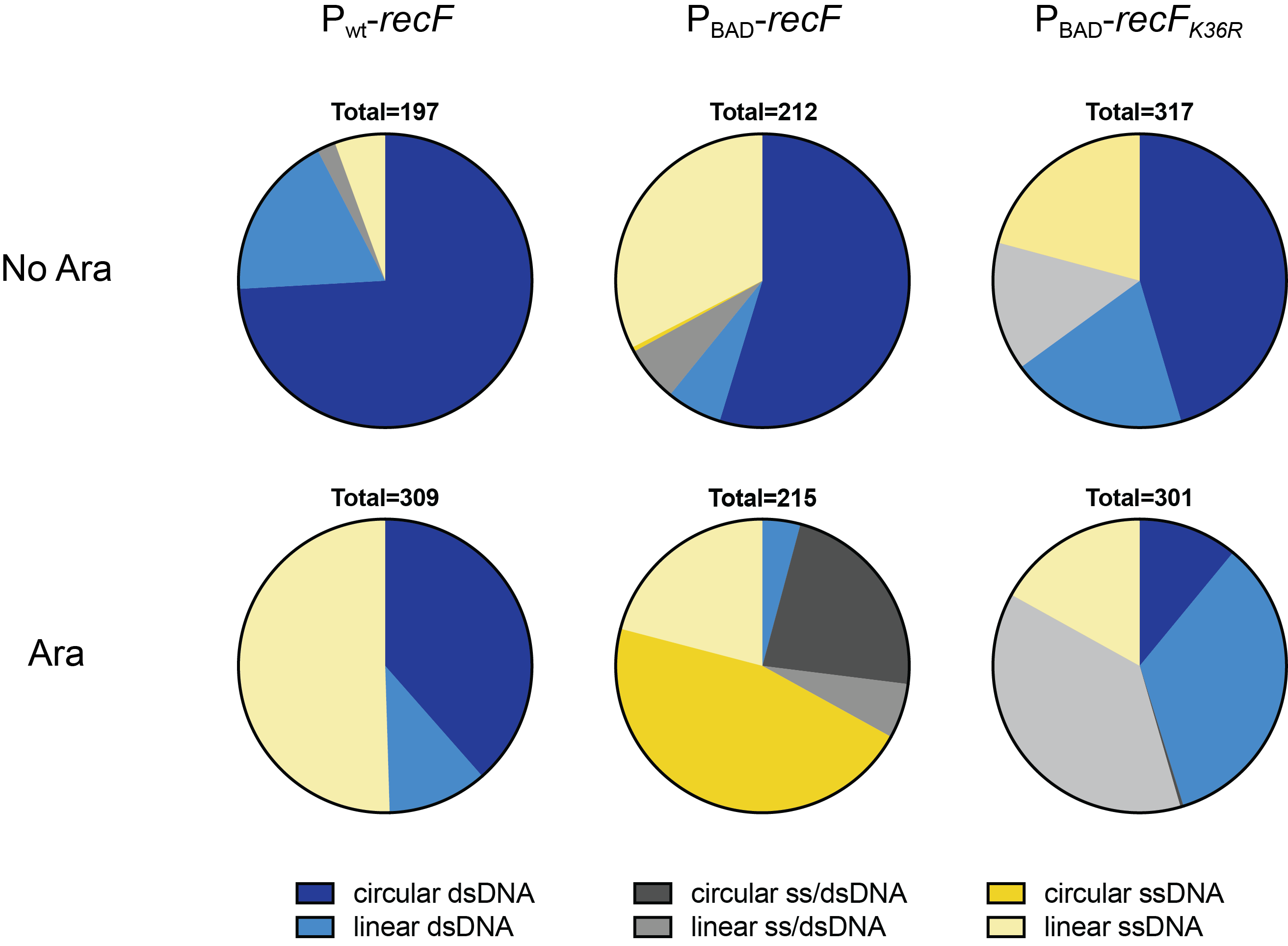


**Supplementary Figure 13: Quantification of pBR322 ss and dsDNA molecules from the cytochrome C electron microscopy imaging.**

The indicated number of pBR322 molecules were counted from the wt, EAW1130 or EAW1148 grown 16h in no or 10% arabinose prior DNA purification. The molecules were classified as circular or linear dsDNA, ds and ssDNA mixed or ssDNA. The repartition of molecules in those 6 categories are represented as a pie chart.


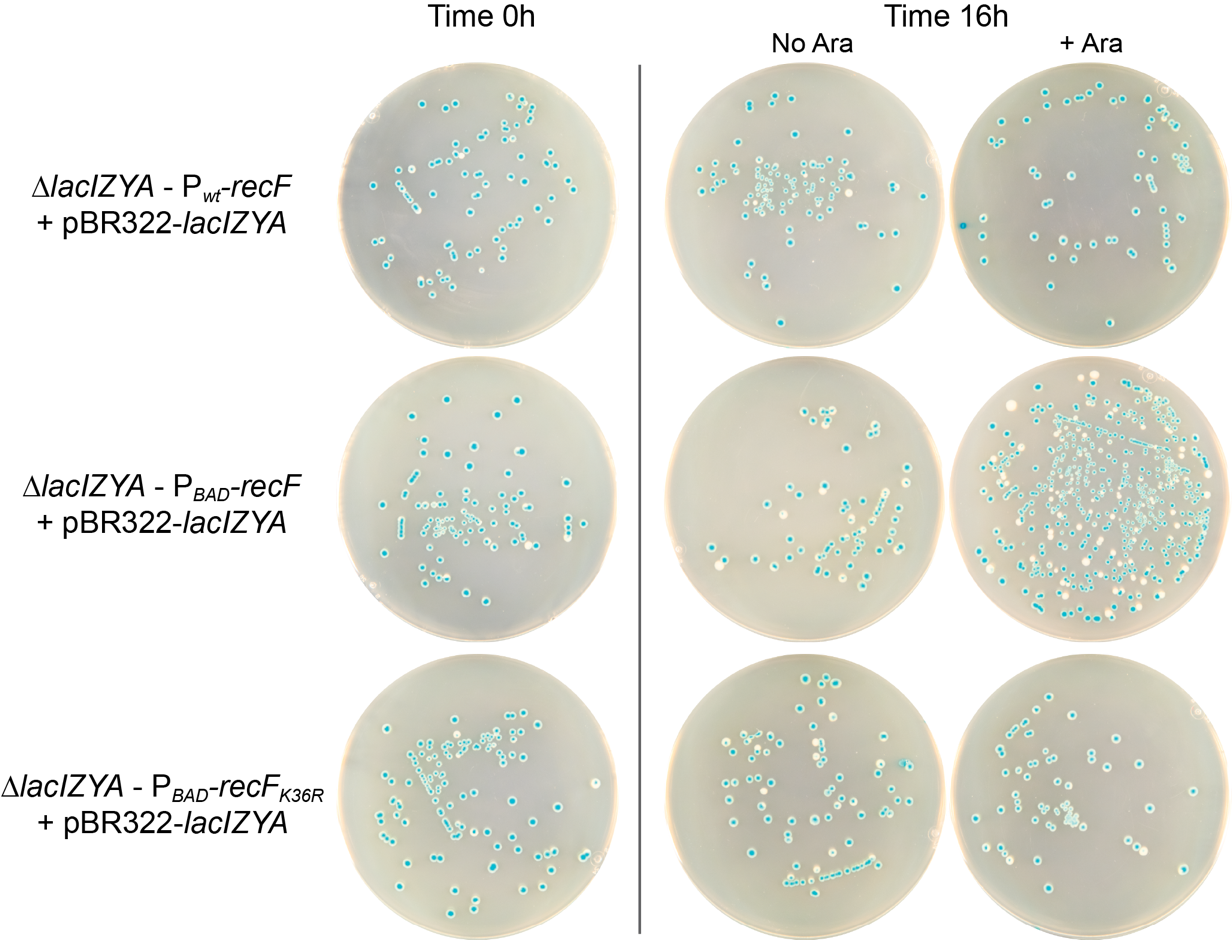


**Supplementary Figure 14: Plasmid loss assay with pBR322 derivatives under RecF over-expression.**

The ability of strains to maintain a pBR322 derivatives plasmid was assayed by blue/white screen. Strains Δ*lacIZYA* encoding either the wild type chromosomal *recF* gene or the constructs P*araBAD* (*recF* or r*ecF_K36R_)* were transformed with the pEAW1232 plasmid. Cells were grown in LB in absence of antibiotic for 16 h in presence or in absence of 10% arabinose. For time 0 and 16 h without arabinose, 50 µL of the dilution 10^-6^ was spread, and for time 16 h + arabinose, 100 µL of the 10^-5^ or 10^-3^ were spread on IPTG-Xgal plates. Plates were incubated in the dark overnight before imaging.

**
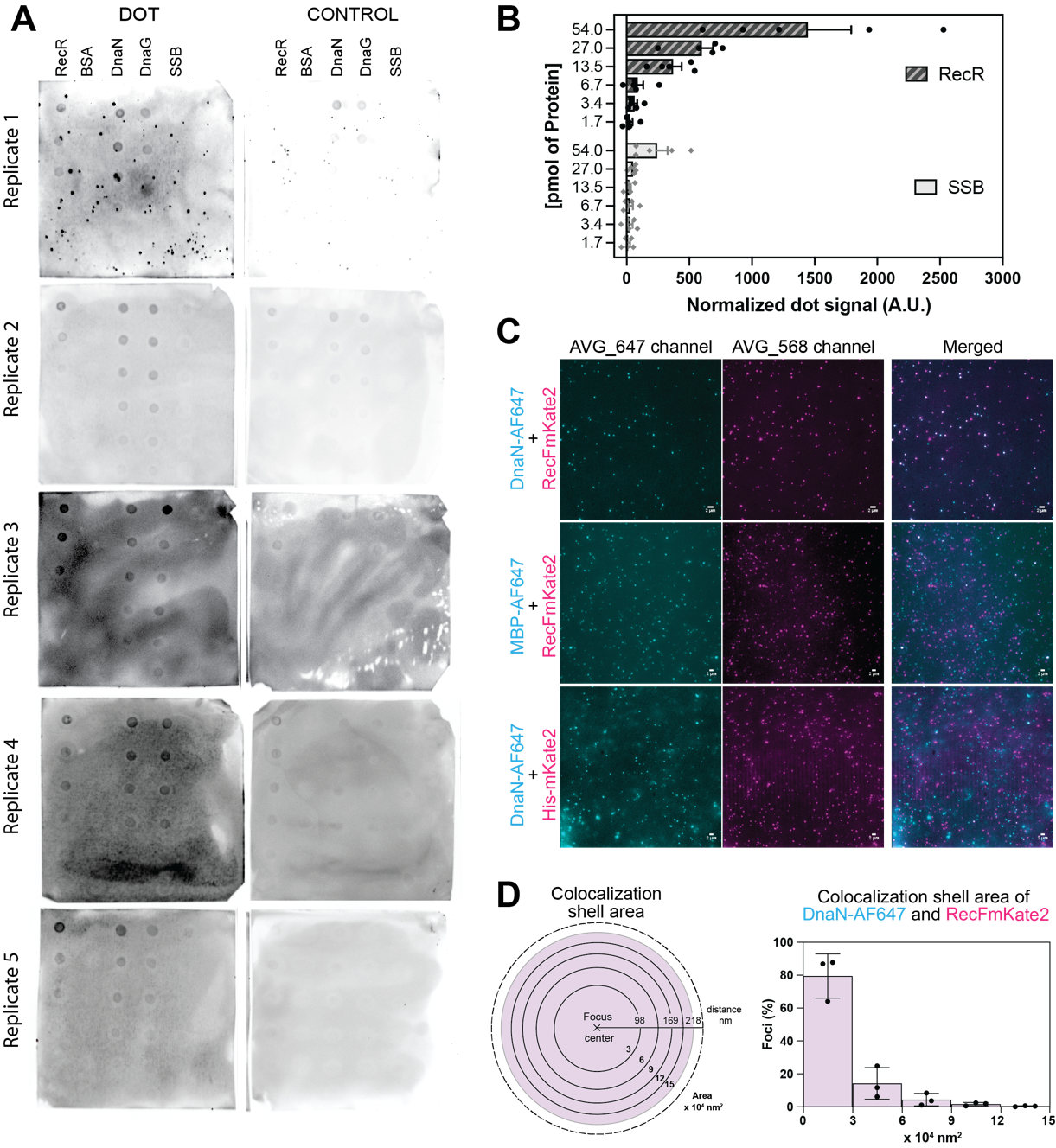
**

**Supplementary Figure 15: Interactions assay of RecF with partners.**

The interaction between RecF and partners was tested *in vitro* by Far western blot (**A** and **B**) and single molecule imaging (**C** and **D**). **A** The fives replicates membranes used for the Far western blot quantification provided in Figure 8. **B** Quantification of the SSB RecF interaction signal compared to the signal of RecR provided in Figure 8. **C** Full field of views of the independent channels 647 (average projection of 150 first images) and 568 (average projection of the 50 first images) and their merges of the three conditions tested. **D** Colocalization shell area schematic and histogram of the colocalization shell area analysis of the 758 RecFmKate2 and DnaN-AF647 found colocalized in Figure 8.


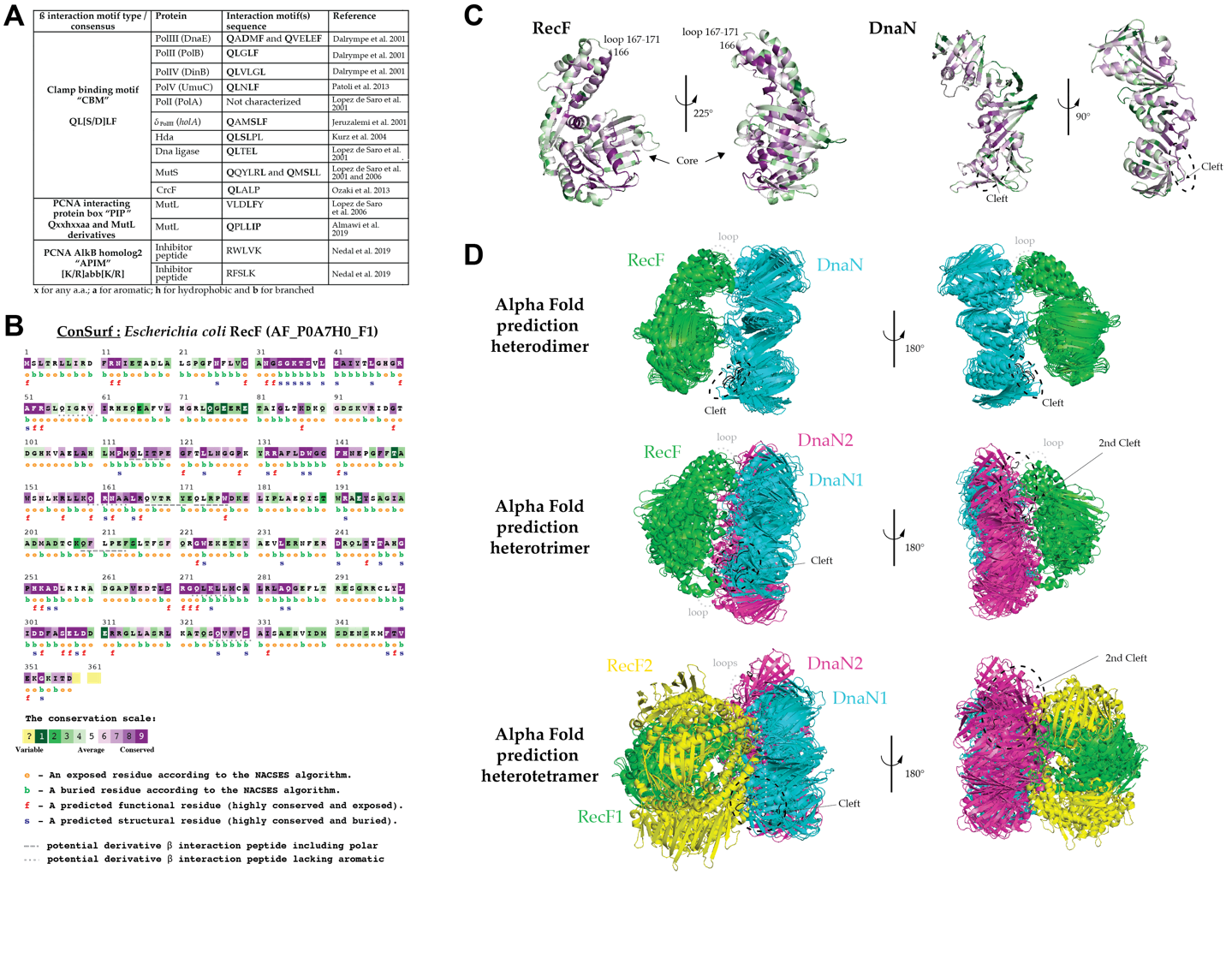


**Supplementary Figure 16: Structural prediction of RecF-DnaN interactions**

Sequence analysis and structural prediction of RecF and DnaN interaction complex. **A** Table presenting known DnaN binding motifs in previously identified partners and consensus of each motif. **B** Conservation analysis of the aminoacids sequences of E. coli RecF generated with ConSurf using the AlphaFold structure prediction of RecF (AF_P0A7H0_F1). Potential derivative (altered) CBM motif in RecF are underlined in grey. **C** ConSurf cartoon representation of the monomeric structure RecF (AF_P0A7H0_F1) and DnaN (2POL – Chain A). RecF loop 167-171 and DnaN cleft residues 362-366 are indicated in the structure. **D** Cartoon representations of the AlphaFold structure overlay of the 5 best models prediction of the heterodimer RecF:DnaN, heterotrimer (RecF:DnaN2) and heterotetramer (RecF2:DnaN2).


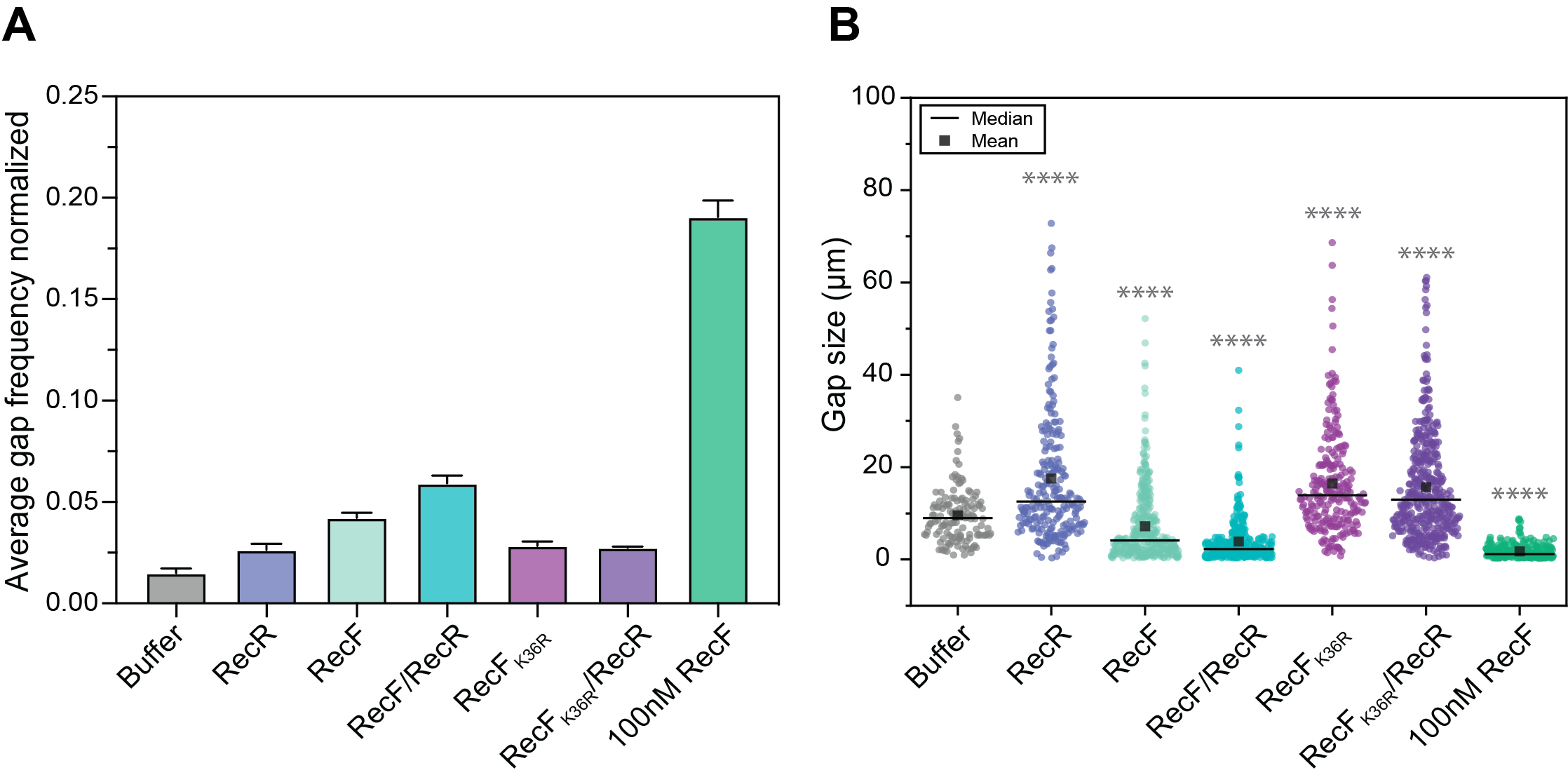


**Supplementary Figure 17: Analysis of gaps formed during pre-assembled rolling-circle assays at the single-molecule level.**

Analysis of the gaps created during pre-assembled rolling-circle replication assays upon addition of RecR (20 nM), RecF (10 or 100 nM) or RecF_K36R_ (10 nM) proteins. When RecR and RecF were combined, 10 nM RecF was used. **A** Histogram representing the average frequency of gap per µm of synthetized DNA ±s.e.m for each condition tested. The number of molecules analyzed is n=260 (Buffer), n=212 (RecR), n= 212 (RecF), n= 157 (RecF and RecR), n=229 (RecF_K36R_) and n= 300 (RecF_K36R_ and RecR). **B** Dot plot representing the size of each gap observed for the indicated condition. Number of gaps n is respectively: n=135 (Buffer), n=230 (RecR), n= 373 (RecF), n= 318 (RecF and RecR), n=231 (RecF_K36R_), n= 410 (RecF_K36R_ and RecR) and n=255**.** Significant differences compare to the buffer condition were evaluated by Mann-Whitney test, ****: p< 0.0001.
